# Supplementary material for: Network Pharmacology of Ginseng (Part II): The Differential Effects of Red Ginseng and Ginsenoside Rg5 in Cancer and Heart Diseases as Determined by Transcriptomics
Source: Pharmaceuticals (Basel). 2021 Sep 30;14(10):1010. doi: 10.3390/ph14101010 (PMC8540751; doi:10.3390/ph14101010)
Supplement: Supplementary file 1 [file pharmaceuticals-14-01010-s001.zip › Supplement 11 WG and HRG HPLC and Supplemental table S4 .pdf]

Supplement 11.

The composition (%) of powdered herbal preparations (HRG80™ and WG™) and their extracts (HRG and WG) used *in vitro* experiments:

	Extract of red ginseng (HRG)	Extract of white ginseng (WG)	Red ginseng Dry powder (HRG80™)	White ginseng Dry powder (WG™)
Rg1	0.093	0.138	0.023	0.035
Re	0.455	2.695	0.114	0.678
Rf	0.322	0.322	0.081	0.081
Rb1	0.183	4.250	0.046	1.069
Rg2	0.000	0.141	0.00	0.035
Rc	1.886	3.402	0.473	0.856
Rh1	0.282	0,00	0.071	0.00
Rb2	0.517	4.309	0.130	1.084
F1	0.019	0.035	0.005	0.009
Rd	1.904	3.810	0.477	0.958
Rg6	0.868	0.058	0.218	0.015
F2	0.524	1.413	0.131	0.355
Rh4	4.356	0.722	1.092	0.182
Rg3-(S-R)	4.307	0.309	1.080	0.078
PPT (20-R)	0.087	0.014	0.022	0.004
Rk1	4.027	0.112	1.009	0.028
C(k)	0,000	0,000	0.00	0.00
**Rg5**	**7.534**	**0.031**	**1.888**	0.008
Rh2	0.924	0.117	0.232	0.029
Rh3	1.817	0.015	0.455	0.004
20S-PPT	0.061	0.187	0.015	0.047
PPD	0.150	0.073	0.038	0.018
**Total**	**30.32**	**22.15**	**7.599**	**5.572**

100 g HRG80 powder contains 25.06 g Extract (DER 4 : 1) including 7.6 g of total ginsenosides
100 g WG powder contains 25.16 g Extract (DER 4:1) including 5.57 g of total ginsenosides.

Abbreviations of test samples used in vitro experiments:

Sample name	Concentration ng/ml
HRG1	10000
HRG2	1000
HRG3	100
HRG4	10
HRG5	0.01

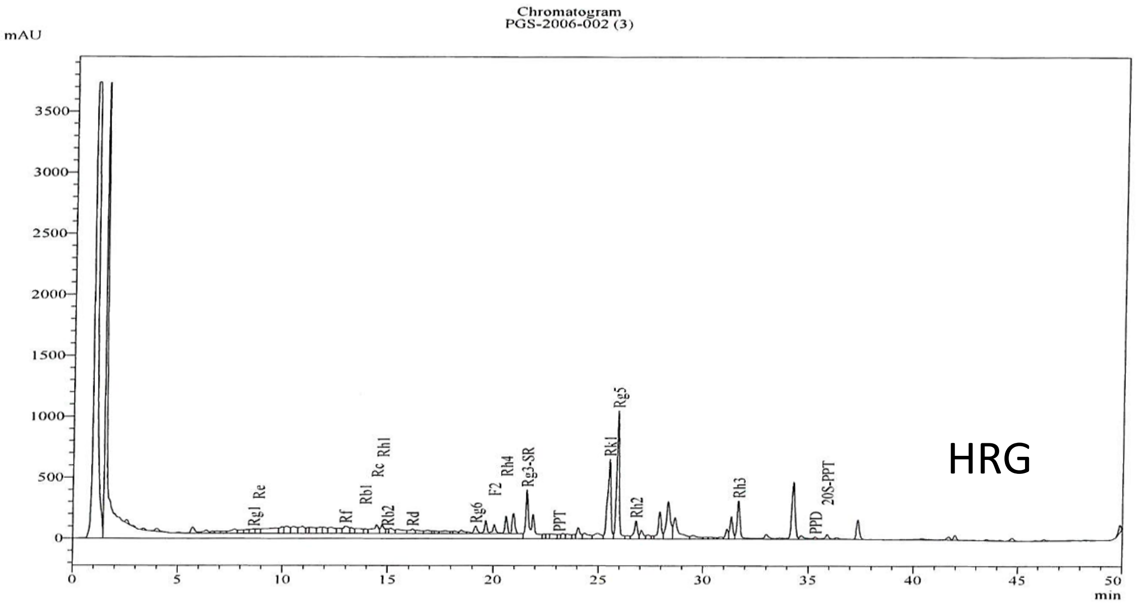

Figure S1. HPLC fingerprint.
