# Supplementary material for: Network Pharmacology of Ginseng (Part II): The Differential Effects of Red Ginseng and Ginsenoside Rg5 in Cancer and Heart Diseases as Determined by Transcriptomics
Source: Pharmaceuticals (Basel). 2021 Sep 30;14(10):1010. doi: 10.3390/ph14101010 (PMC8540751; doi:10.3390/ph14101010)
Supplement: Supplementary file 1 [file pharmaceuticals-14-01010-s001.zip › Supplement 2 Venn diagrams Tables S2 and S3.pdf]

Supplemental table S2

Sample names	number of genes
HRG2	332
HRG4	323

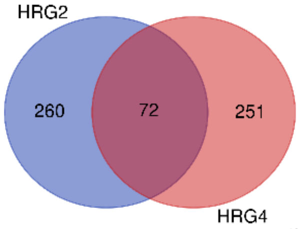

samples	Total genes	genes
HRG2 HRG4	72	*MLX CD34 SESN3 CLCNKA PIR CHID1 SRPK1 NRP1 EMC2 FASN SDSL UGDH ZNF212 RIN3 PDCD4 CAPRIN1 NQO1 CMC2 CCNE1 SERPINB1 UBR3 PIGQ RTN3 KRTAP10-7 LRRC75A TBL1XR1 THBS1 CLP1 PCMTD1 ITSN2 ZCCHC9 TAPBP ZEB2 CHRM2 SH2D2A SMIM8 RPL23 ATP6AP2 CCDC6 FAF1 LMBRD2 SARDH TAB2 CD300LF B4GALT2 SPP1 ORAI1 INTS10 TM2D1 ZC3HAV1L FASTK NCSTN IMPACT CCND1 CTH COL6A1 GRHL3 DMXL1 PRUNE2 C18orf54 EPHA8 HNRNPL SLC35E2B GIGYF1 POC1B FOSL1 SMAD6 RNF165 PCSK1N DNAJC19 METTL1 DEPDC1*
HRG2	260	*PODNL1 NSUN2 DALRD3 DPY30 DLG1 SLC25A40 DIAPH3 VEZF1 DNAJC14 OGT BBS2 GRB10 TPX2 ME2 ERI1 ARVCF NUBP2 GPSM2 FAM126A SETD2 TBX10 TM9SF1 PAIP1 PFAS DHX35 EXT1 PHLDA3 EIF5B SLC35F5 NT5C3A EXOC5 KRTAP1-3 SOX4 NIFK PLD2 SERTAD3 DNAJB1 C8orf58 OGN PCOLCE ANGPTL4 BBS9 ARHGAP17 DAD1 CDC25B MYO9A MFGE8 MGAT2 GLT8D2 LAMB1 TLR1 G3BP2 CHTF8 ANAPC2 JMJD6 RHOC MED17 DYNC1LI1 WDR45 KATNB1 PCSK4 ATP6V0B TFDP1 FBLIM1 DUSP4 ITPRID2 RBPJ SFR1 TTC28 THOC2 DACT3 CBR3 TPGS2 PURA GASK1B DCUN1D4 ABI1 SLCO2A1 DUS3L NEO1 C1orf216 PLA2G15 GMCL1 XPO1 WDR43 ITPKB GMPPA MYH9 ARL6IP5 TTYH3 CAPN11 TMEM59 SIL1 NECAB1 UNC93A CAPRIN2 TMEM104 EFNB2 TAS1R2 FRA10AC1 IRF3 TTI1 SLC30A1 CCDC186 TUBG1 RAD54L CDC45 TMEM164 ABI2 HLA-G IL6ST FAM32A TIMMDC1 PRAF2 TAS1R1 ETS1 TBX18 FUT4 VAMP3 ATP5PD ZNF616 SAC3D1 PUS1 SLC41A2 IST1 TMEM170B CDC73 MINDY2 PTPRA PSMD4 LAS1L ZNF606 LIN9 E4F1 ZCCHC8 PARP11 OSBPL11 FNDC7 MOGS BICDL1 BRMS1L PANK2 TM9SF3 PRMT9 STAC2 SMPD1 COL6A2 EXOG PURB ASAP1 RPS27L GTPBP6 UPRT CACNA1H FN3KRP SMAD7 RNF7 TANC2 RNASET2 RPS7 UNC13C NYNRIN PABPC1 SERF2 MBD4 MED25 CD47 SMCO2 IL34 ADAM9 ZSCAN29 KCNC3 EFNA3 KCTD16 ASPSCR1 AGK ARMC7 B2M KIF2C HCFC1R1 PSMD7 INSM2 RAD51C DMRTA1 BCL7A MYO5A RNF130 CAV2 ICE1 CDKL5 HIRIP3 TIMM29 NOTCH2 COLGALT1 ERICH1 TOR2A CRTC2 MADD BLOC1S6 RPN2 STRN3 CLEC11A SFT2D1 ALMS1 PFKFB4 DCN DCTD SCFD1 TNS1 KLC3 BSDC1 IFNAR2 WRAP73 CRAT PMCH C2orf69 DNAJB6 VPS53 CHST2 MAGED1 KAT6A RFC4 C19orf44 TPST2 SPNS1 MFSD14B ADGRA3 NEMP1 TMF1 TNFAIP3 RPS6KC1 HNRNPA1L2 AGA AMD1 GATC ITGB5 FAM149B1 PTTG1 GPAT3 USP1 PCNX1 COMT CHST3 SLC2A1 EEF1G SLC25A43 METAP1 C15orf40 TMEM94 MDM4 BCKDHB TEPSIN FAM171A2 CDX1 ISYNA1 ZNF780A TMEM89 GZF1 RBKS RIN1*
HRG4	251	*CHPT1 PTPRCAP TMBIM4 SOS1 CITED2 MATR3 MFAP4 CGNL1 MOV10 LGR6 HNRNPD ZNF131 SOD2 HMG20B CCDC97 NSMCE2 MRPL32 ASL RALGAPB ALOX15B USP20 SLC22A23 CEP135 CDYL2 TBC1D31 LHFPL2 SLC39A7 CSTF2 RIOK1 SPPL2A AIDA ITM2B METTL9 ZNF202 GINS1 CLCC1 CCDC66 CSGALNACT2 RHOU SERTAD4 CCDC191 KREMEN1 NKD1 POLL FBXO9 APPL2 UBE2I MRPL13 GTF2F1 MINK1 REEP5 USF3 UQCC3 IGFBP6 ARNT TCAF2 SRD5A1 CDK1 POLR2B THAP12 ZNF226 FABP5 ACBD5 MFSD11 CARMIL1 BLCAP SEC14L1 QTRT1 SH3GLB1 SPRTN AP3M1 CD6 FEZ1 MT-ND3 METRNL IPP STIM1 DEPDC5 PCDH18 FLNC ABHD12 RGL2 C15orf65 C2CD2L PMPCB TBXA2R NDUFC1 CIITA TRRAP FAM98C KCNQ5 GGCX STK40 IPO13 MCC CYP51A1 FADS2 IL1RL1 SLC9A3R1 RB1 FAM43A FKBP4 TMEM161A MTA1 VASN SMC6 CSRP3 TADA3 CHMP2B KIF13A RPL13 ADIPOR1 NR3C1 HAT1 NIPA1 SPATS1 WNT3A FAM181B TRMT10A FAM71F1 FAM149A SLC30A5 DIPK1A CLDN9 YJU2 CCDC15 SOX9 PRPF38A FTH1 COPB1 TM4SF1 SRSF9 GPR150 GINM1 SMG6 LLPH TTN SGF29 GPR137 RAD51D SRA1 SELENOF SOHLH1 FKBP10 NIT2 CUL2 TCF7L2 OGA SNX13 KCTD13 ZNF830 COX6A2 HLA-A NCOA2 S100PBP DAP CCDC71L DPP7 UBIAD1 CRCP RAD54B SLC6A9 KLHL20 SDR16C5 SRM APAF1 STXBP5 ETFB XRCC5 RBM39 KLC2 SLPI NAP1L4 BOLA3 NUP205 GDF1 KDM2B TLCD1 RNF181 POLD1 E2F4 ZBTB7A PMS1 STAC3 GSK3A KRT6B NUDCD2 EXOC7 ECHDC3 RNF115 PTGS1 MUC20 GPX2 RAMP3 C11orf65 SELENOS EPB41L3 TXNL4A MAP6 EPS15L1 EEF1B2 TBCCD1 CASP4 FMNL2 HMGA1 NFIC PDHB JUN SHKBP1 ASB9 UNKL PGS1 ACTG1 DDX3X WNT10B PEX6 CPQ RPL24 PXK LZTS3 ATL3 CP LRRC14 POLA2 ARL5A PROCR ATAD3A KRT40 CCDC28A SLC17A5 GLTPD2 NUDT21 EPHB4 PAPOLB NRTN EVX2 MEDAG RETREG1 CAMK2N2 RBMS1 SMURF2 KLHDC1 BBX RFK AVP TCTN3 NDUFC2 TRIM24 HELQ SLC38A6 LIPT2*

Supplemental table S3

Sample name	number of grnes
HRG2	461
WG	344

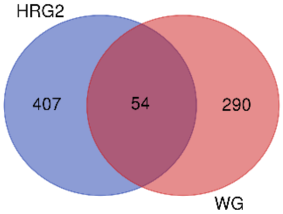

Names	total	elements
HRG2 WG	54	CNPY2 DNAJC14 GRB10 ERI1 4930519F16Rik AP3S2 NT5C3A G3BP2 PDCD4 CCL19 Luzp4 (includes others) CMC2 RPS15A SFR1 DACT3 PIGQ KRTAP10-7 LRRC75A Nnt ARL6IP5 RSRC1 TAS1R2 IL6ST TP53INP2 AI987944 (includes others) STAC2 ASAP1 RPS27L C8orf76 RNASET2 ORAI1 ADAM9 INTS10 BCL7A RNF130 ICE1 RSC1A1 BLOC1S6 YIPF5 Clasrp SFT2D1 KAT6A TPST2 HNRNPL RPS6KC1 FAM149B1 Ezhip RAP2B PCNX1 FAM171A2 TMEM89 DEPDC1 RBKS RIN1
HRG2	407	PODNL1 MLX NSUN2 DALRD3 C3orf14 THAP2 DPY30 DLG1 SMARCD3 CD34 Foxp1 H2AZ2 SLC25A40 DIAPH3 IER2 VEZF1 OGT BBS2 TPX2 ME2 ARVCF Scd2 NUBP2 GPSM2 CHCHD3 SETD2 FAM126A TBX10 TM9SF1 PAIP1 PFAS DHX35 EXT1 CRACDL Smim10l2a SESN3 MSRB3 PHLDA3 EIF5B SLC35F5 CLCNKA GOT1 EXOC5 TSPAN12 KRTAP1-3 SOX4 NIFK SERTAD3 PLD2 PIR DNAJB1 CHID1 SRPK1 Prh1/Prp2 C8orf58 OGN PCOLCE NRP1 NAT8B ANGPTL4 AKR1E2 EMC2 BBS9 ZNF597 FASN SDSL ARHGAP17 RAB3GAP1 DAD1 CDC25B INHBA CLN8 MYO9A ACTL6A MFGE8 UGDH RNF167 MGAT2 GLT8D2 LAMB1 H2BC13 2610001J05Rik TLR1 Rbmy (includes others) Ccl7 CHTF8 ANAPC2 JMJD6 ZNF212 RIN3 ZNF691 RHOC MED17 CAPRIN1 NPHP1 DYNC1LI1 WDR45 KATNB1 FGF7 PCSK4 SPOP ATP6V0B NQO1 SNAP23 TFDP1 AMN1 Slurp2 FBLIM1 CCNE1 DUSP4 SSU72 RBPJ ITPRID2 Zfp108/Zfp93 TTC28 Gm14418 THOC2 CBR3 TPGS2 PURA GASK1B DCUN1D4 ABI1 SLCO2A1 SERPINB1 Casp12 DUS3L NEO1 DDIT3 C1orf216 PLA2G15 CFL2 UBR3 GMCL1 CHMP1B XPO1 RTN3 WDR43 ITPKB GMPPA ATP6V0C MYH9 TTYH3 Gm11564 CAPN11 Gk CA10 TBL1XR1 CHST11 TMEM59 SIL1 NECAB1 UNC93A CAPRIN2 TMEM104 EFNB2 THBS1 FRA10AC1 LDHB IRF3 TTI1 CLP1 Ly6a (includes others) SLC30A1 CCDC186 TUBG1 RAD54L CDC45 TMEM164 ABI2 EZR HLA-G FAM32A UXS1 TIMMDC1 TAS1R1 PRAF2 ARF1 ETS1 PCMTD1 RPL18 ITSN2 TBX18 FUT4 VAMP3 ATP5PD ZNF616 ZCCHC9 SAC3D1 PUS1 SLC41A2 TAPBP IST1 TMEM170B GMFB CDC73 MINDY2 PTPRA PSMD4 LAS1L H3-3A/H3-3B Gm16434 (includes others) ZEB2 ZNF606 LIN9 RHOJ UNC50 CPNE2 CHRM2 E4F1 PSMF1 GLIPR1L2 ZCCHC8 SH2D2A Gm5643 Gm16253 PARP11 Tmem115 SMIM8 EMP1 H2al2a (includes others) C030037D09Rik RPL23 YIPF6 OSBPL11 FNDC7 MOGS ATP6AP2 CCDC6 BICDL1 BRMS1L PANK2 PRMT9 TM9SF3 FAF1 TMEM242 SMPD1 COL6A2 EXOG PURB POLR1G GTPBP6 CACNA1H UPRT LMBRD2 H1f1 SARDH FN3KRP SMAD7 ST3GAL3 TAB2 CD300LF RNF7 TANC2 Brd4 RPS7 MAD2L1BP TMEM120A UNC13C BNIP3L NYNRIN B4GALT2 Hmga2 PABPC1 TMEM50A MBD4 MED25 SERF2 SPP1 CD47 H3C6 IL34 SMCO2 DDX52 ZSCAN29 KCNC3 EFNA3 KCTD16 ASPSCR1 AGK ARMC7 Mup1 (includes others) B2M KIF2C RND2 HCFC1R1 PSMD7 INSM2 DMRTA1 RAD51C TM2D1 MYO5A ZC3HAV1L CDC42EP2 P2RY2 FASTK LDB1 Commd6 CAV2 NCSTN IMPACT CDKL5 HIRIP3 CCND1 TIMM29 NOTCH2 Norad CTH COLGALT1 COL6A1 ERICH1 TOR2A CRTC2 MADD RPN2 STRN3 CLEC11A Mia2 GRHL3 ALMS1 CARD19 PFKFB4 DMXL1 DCN DCTD SCFD1 TNS1 Zfp871 KLC3 IFNAR2 BSDC1 F8A1 (includes others) PRUNE2 PMP22 Abcb1b WRAP73 CRAT C18orf54 PMCH C2orf69 Snrpg DNAJB6 VPS53 EPHA8 Gpr137b-ps CHST2 MAGED1 METTL17 RPS4Y1 RFC4 C19orf44 SPNS1 PLEKHF2 SLC35E2B MFSD14B ADGRA3 NEMP1 PARS2 Raet1b Srek1ip1 MSANTD4 TMF1 TNFAIP3 GIGYF1 POC1B HNRNPA1L2 FOSL1 AMD1 AGA GATC ITGB5 EVL PTTG1 GPAT3 ZNF260 MED30 ALKBH4 USP1 WDR73 2310001K24Rik COMT SMAD6 CHST3 SLC2A1 EEF1G SLC25A43 ADPRS METAP1 C15orf40 TMEM94 MDM4 BCKDHB ACKR3 TEPSIN CDX1 RNF165 ISYNA1 UBE2D1 ZNF780A PCSK1N 2510002D24Rik METTL1 DNAJC19 Fxyd2 B230307C23Rik COX6A1 GZF1
WG	290	TRIP6 ABCD3 PTPRCAP PDHA1 DHCR24 FOXP4 H3C12 CSPG4 EIF2AK2 DUSP19 LCORL FOSL2 PGAM5 CDRT4 APIP MOXD1 MYLIP Rpl23a MRPS35 MFAP4 SVBP INSIG2 Rhox4b (includes others) SMARCC1 HOMER3 STK16 Gtf2ird2 Eif1 Pcmt1 LONP1 RALGAPB CDK9 ZNF365 DUSP22 OAZ2 CAPZA1 VSTM2L S100a2 EIF1B CHSY1 ZNF219 RABGGTA ATP5MG LIAS RPL35 LARP7 PYGB RHOU PTPN11 GDPD5 DHX8 C19orf71 YIPF4 OXSM SPTY2D1 PREB BCLAF1 Lbhd2 IKBKB

		GSPT2 FBXO9 SEPTIN6 MRPL13 CLASP1 CAPZA2 PCYT1A LRRC8D SLC49A4 RNF146 SNX9 DLK2 BNIP1 CFAP73 STX12 RPL15 PRC1 UBA7 POLR2B CALU THAP12 GAS2L1 FAM98B MASP2 ZNF175 TEAD3 CCNB1IP1 ACOX1 CCT2 ID1 PABPC4 NEU4 CLK2 AUNIP COG1 KLF5 SLC25A11 Ces2g NCBP2 UBE2E1 EIF3J ESS2 ZNHIT2 POGZ MRPL34 FUCA2 Pwwp4b (includes others) FRAT1 Gm5801 KIF16B ELOF1 N4BP2 SRD5A3 NEXN TMEM109 ACADL CCDC136 AU016765 IL6R FER TMEM234 PAN2 TRRAP SMC1A CDH8 FBXL17 DRAM2 LRRC59 MCC SELENOI MYL6 FADS2 HSPA4L CHMP1A C2orf81 MGST1 BZW2 ADAR EGR1 KCTD5 WIPF3 TMEM161A MAP3K4 HIF1AN TMEM165 RAB21 ACTR5 SCAND1 CCDC173 ARL3 S100A10 EIF2AK4 BATF3 Miat FTL SKAP2 CCL27 MMP23B ASPH DYRK4 NSMCE3 TMEM163 CKS1B Gm10563 STUB1 Arxes1/Arxes2 DOK4 LEPROT RASA4 ZNF324 MTHFD2 TTC13 TGIF2LX SNORC EIF1AY CTBP2 FZD7 CNTF SGF29 NAA20 C1orf198 COA8 BAG5 TTC23L DYRK1A Zfp740 MPC2 GSTZ1 ARFGEF1 CDK19 MYCBP ANGEL1 AVPI1 KIAA1328 RSPO1 DCAF11 Sprr2b ASPHD2 PLEKHA4 LYSMD1 SPATA6 ZNF442 OTULIN ZNF593 TEDC2 KALRN H1-0 FDXR PAX3 RBM39 GFM1 MAP1A Gm3839 PROX2 PLD5 PARP4 HEXB MAP7D3 ZNF644 H2-T10 PRKDC PPP1R14C ARHGEF25 C1orf127 PSMD12 PMS1 MVD Rplp1 (includes others) TOP2B SPSB2 EXOC7 RABL2A RBM3 H2BC14 NFATC3 Gm13031 WDCP CYB5A CRYGN PRMT6 CLEC4F WDR13 OSGEPL1 MAGEB1 TINAGL1 PPP1R12C TOE1 TPR EEF2K MRPL11 EPC1 Bex1 Defb10/Defb11 NAGK 3110021A11Rik BIRC2 RP2 Gm3383 (includes others) 5730460C07Rik MCAT USP39 RPL24 GIT2 NUDCD1 VPS13D ABHD14B SUOX ALAS1 ZNF267 A930004D18Rik RELL2 SF3B3 SNX29 PALS2 WWP1 TMEM62 C920021L13Rik PRODH2 PSME4 EPHB1 EVX2 CAMK2N2 HLX Olfr49 SNX17 B3GNT2 S1PR3 RERE GPR89A/GPR89B LIPT2 ARHGDIB
