# Supplementary material for: Network Pharmacology of Ginseng (Part II): The Differential Effects of Red Ginseng and Ginsenoside Rg5 in Cancer and Heart Diseases as Determined by Transcriptomics
Source: Pharmaceuticals (Basel). 2021 Sep 30;14(10):1010. doi: 10.3390/ph14101010 (PMC8540751; doi:10.3390/ph14101010)
Supplement: Supplementary file 1 [file pharmaceuticals-14-01010-s001.zip › Supplement 3 Neuroinflammation signaling.pdf]

Neuroinflammation involves numerous cell types, acts to clear neuronal damage, and plays a key role in maintaining the homeostasis of CNS. Homeostasis can be lost through various regulatory failures, or when humoral immune components cross the blood-brain barrier, causing chronic inflammation with excessive cell and tissue damage, which is associated with neurodegenerative diseases.

WG 10,000 ng/ml

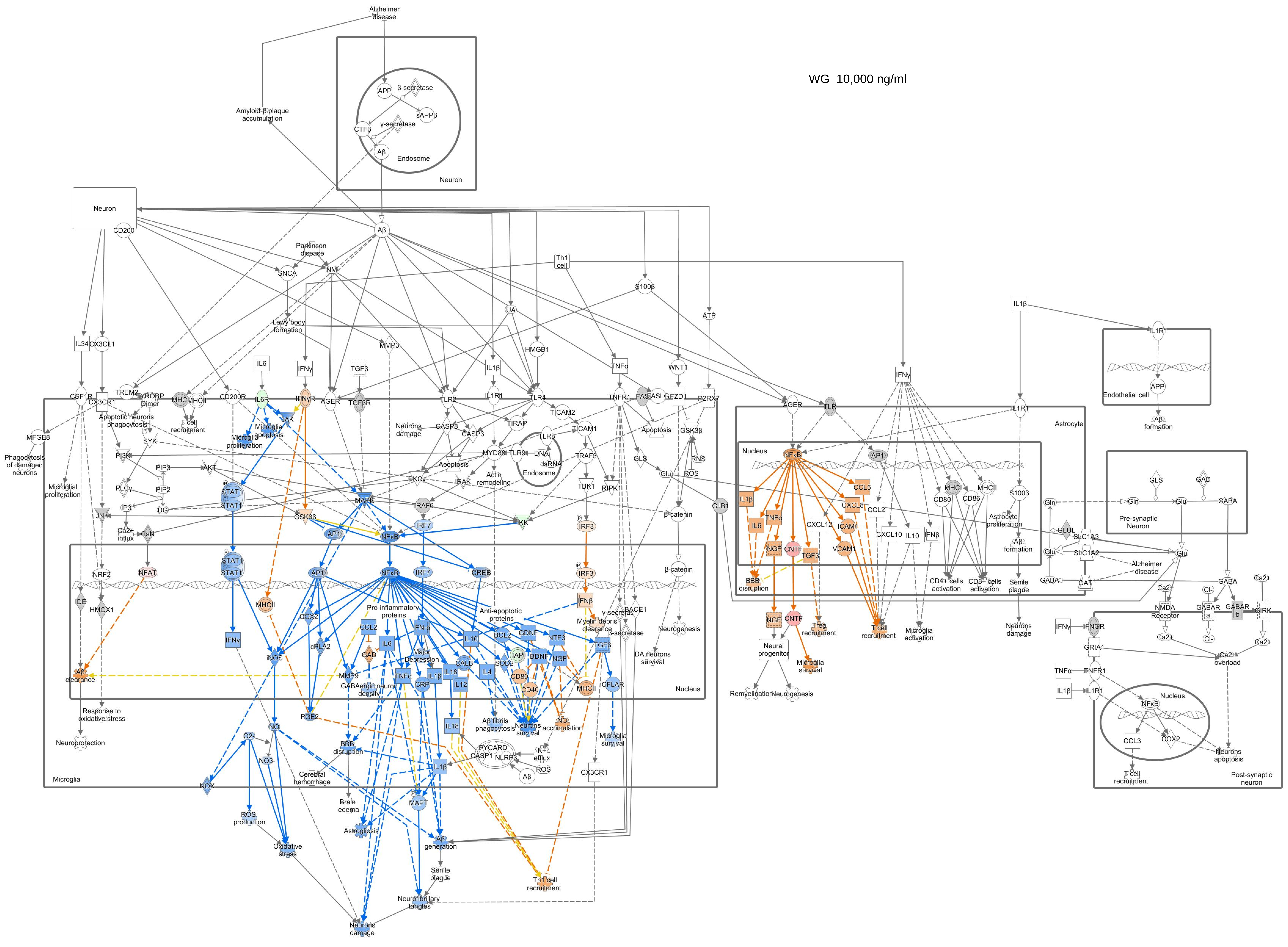

© 2000-2021 QIAGEN. All rights reserved.

Neuroinflammation involves numerous cell types, acts to clear neuronal damage, and plays a key role in maintaining the homeostasis of CNS. Homeostasis can be lost through various regulatory failures, or when humoral immune components cross the blood-brain barrier, causing chronic inflammation with excessive cell and tissue damage, which is associated with neurodegenerative diseases.

HRG80 10,000 ng/ml

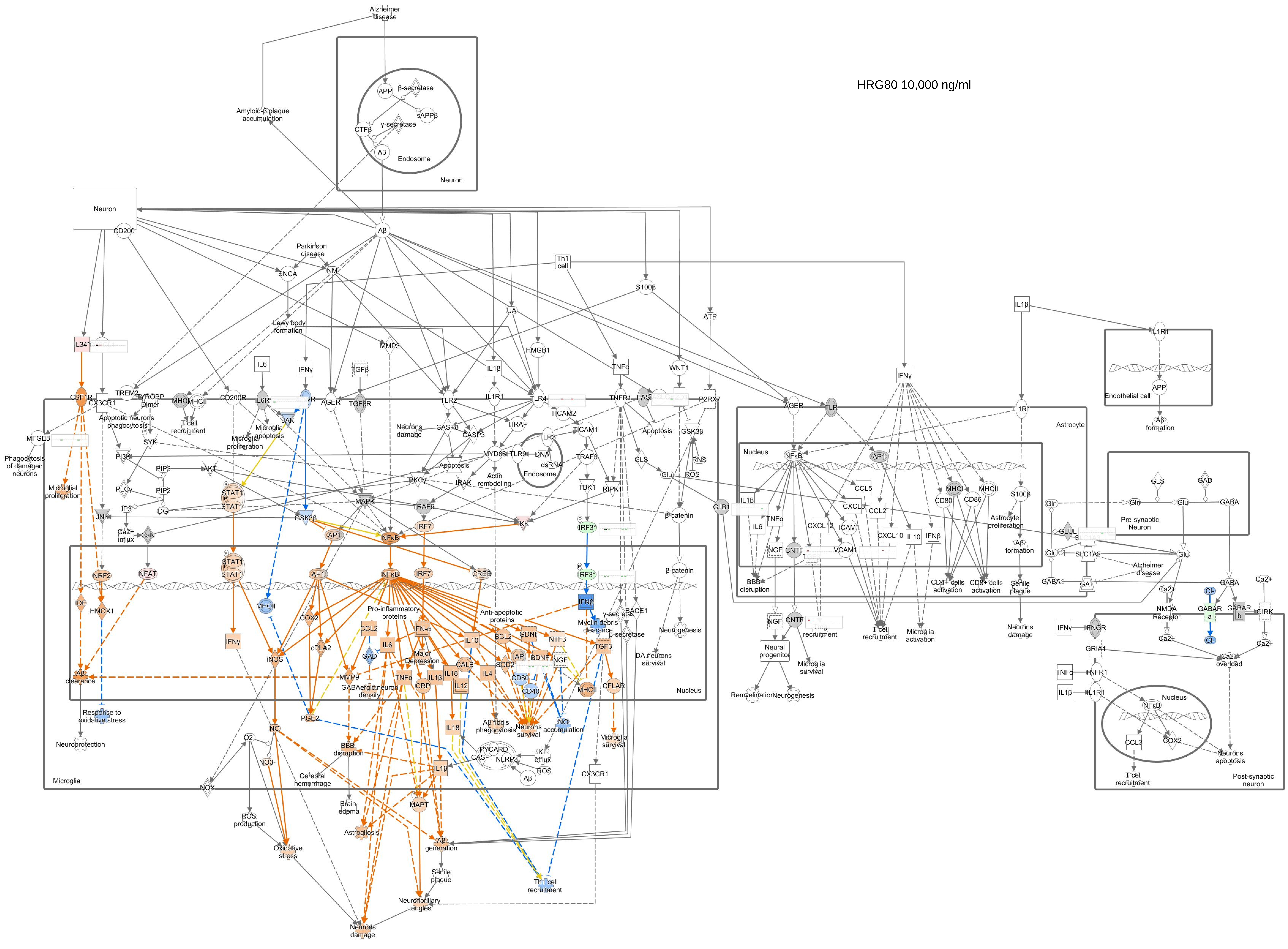

© 2000-2021 QIAGEN. All rights reserved.

Neuroinflammation involves numerous cell types, acts to clear neuronal damage, and plays a key role in maintaining the homeostasis of CNS. Homeostasis can be lost through various regulatory failures, or when humoral immune components cross the blood-brain barrier, causing chronic inflammation with excessive cell and tissue damage, which is associated with neurodegenerative diseases.

HRG80 1,000 ng/ml

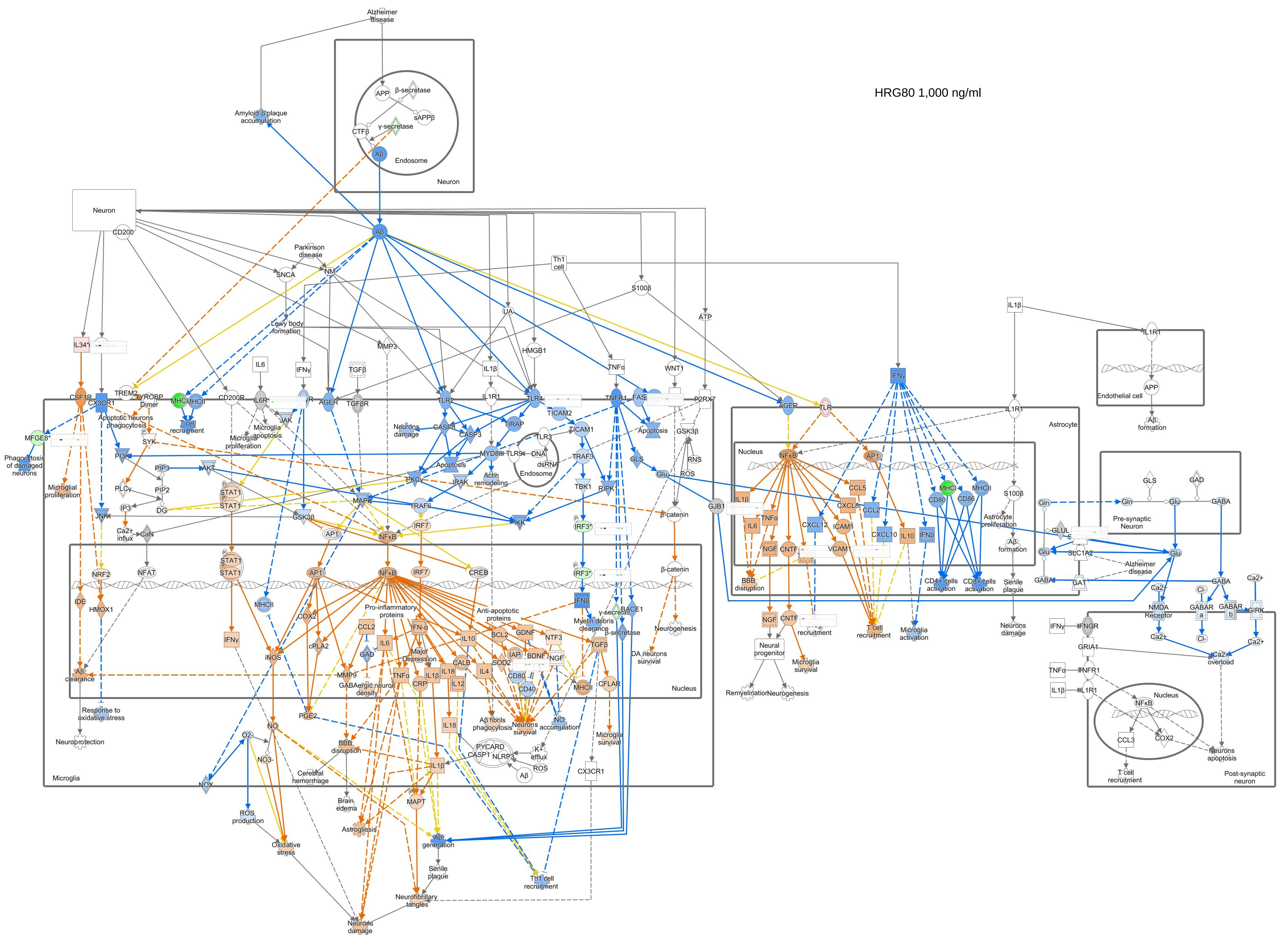

© 2000-2021 QIAGEN. All rights reserved.

Neuroinflammation involves numerous cell types, acts to clear neuronal damage, and plays a key role in maintaining the homeostasis of CNS. Homeostasis can be lost through various regulatory failures, or when humoral immune components cross the blood-brain barrier, causing chronic inflammation with excessive cell and tissue damage, which is associated with neurodegenerative diseases.

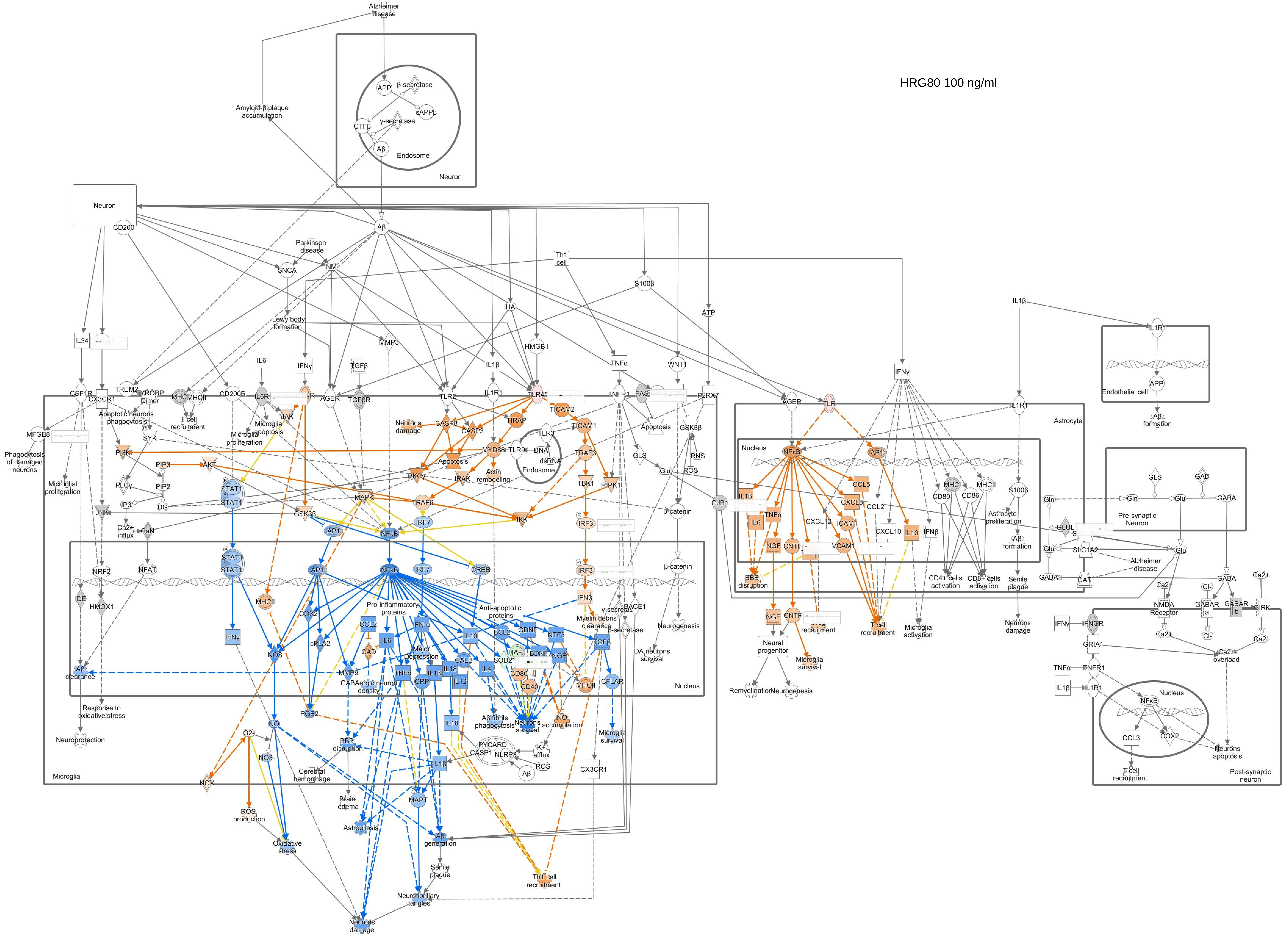

© 2000-2021 QIAGEN. All rights reserved.

Neuroinflammation involves numerous cell types, acts to clear neuronal damage, and plays a key role in maintaining the homeostasis of CNS. Homeostasis can be lost through various regulatory failures, or when humoral immune components cross the blood-brain barrier, causing chronic inflammation with excessive cell and tissue damage, which is associated with neurodegenerative diseases.

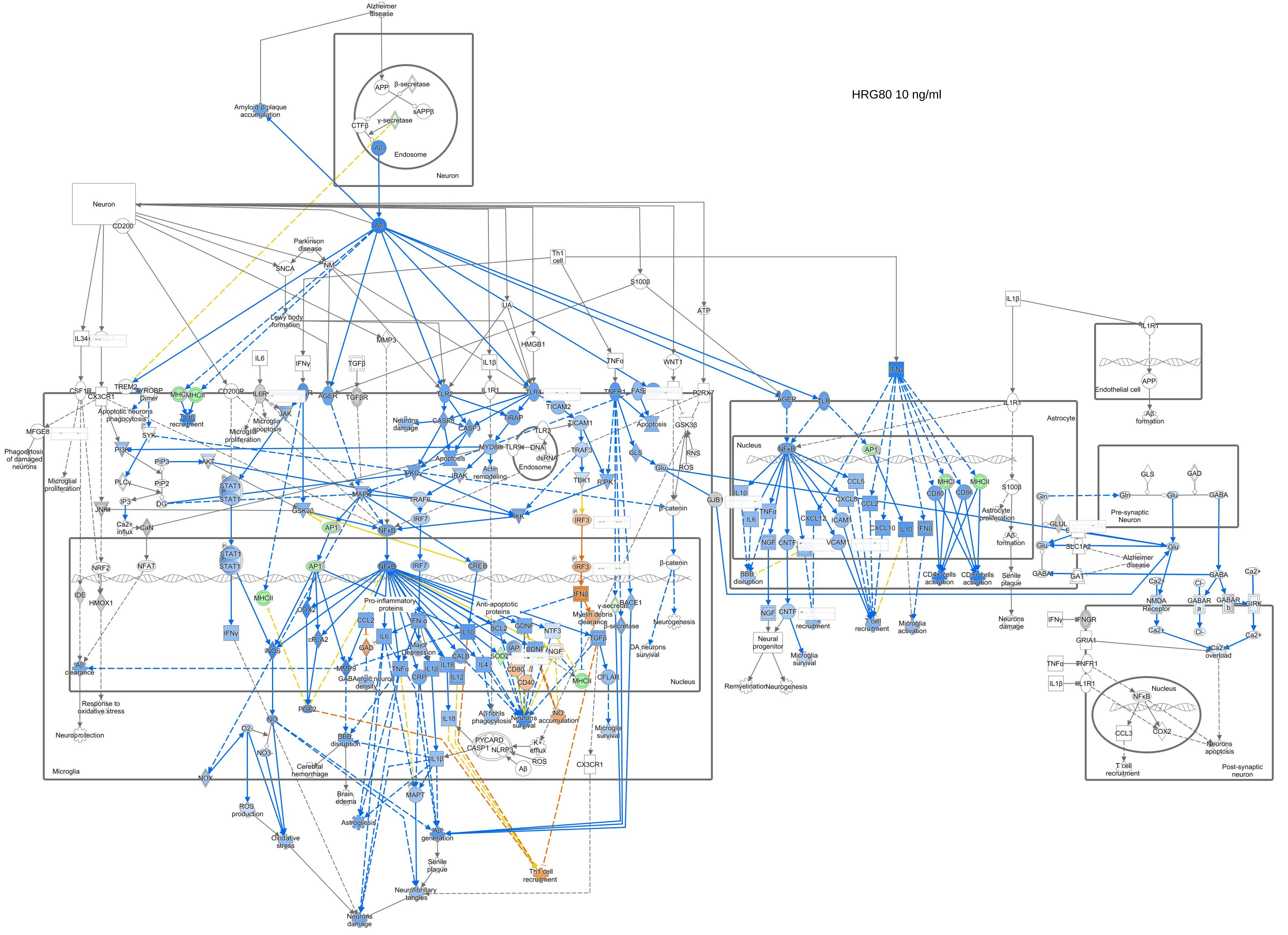

© 2000-2021 QIAGEN. All rights reserved.

Neuroinflammation involves numerous cell types, acts to clear neuronal damage, and plays a key role in maintaining the homeostasis of CNS. Homeostasis can be lost through various regulatory failures, or when humoral immune components cross the blood-brain barrier, causing chronic inflammation with excessive cell and tissue damage, which is associated with neurodegenerative diseases.

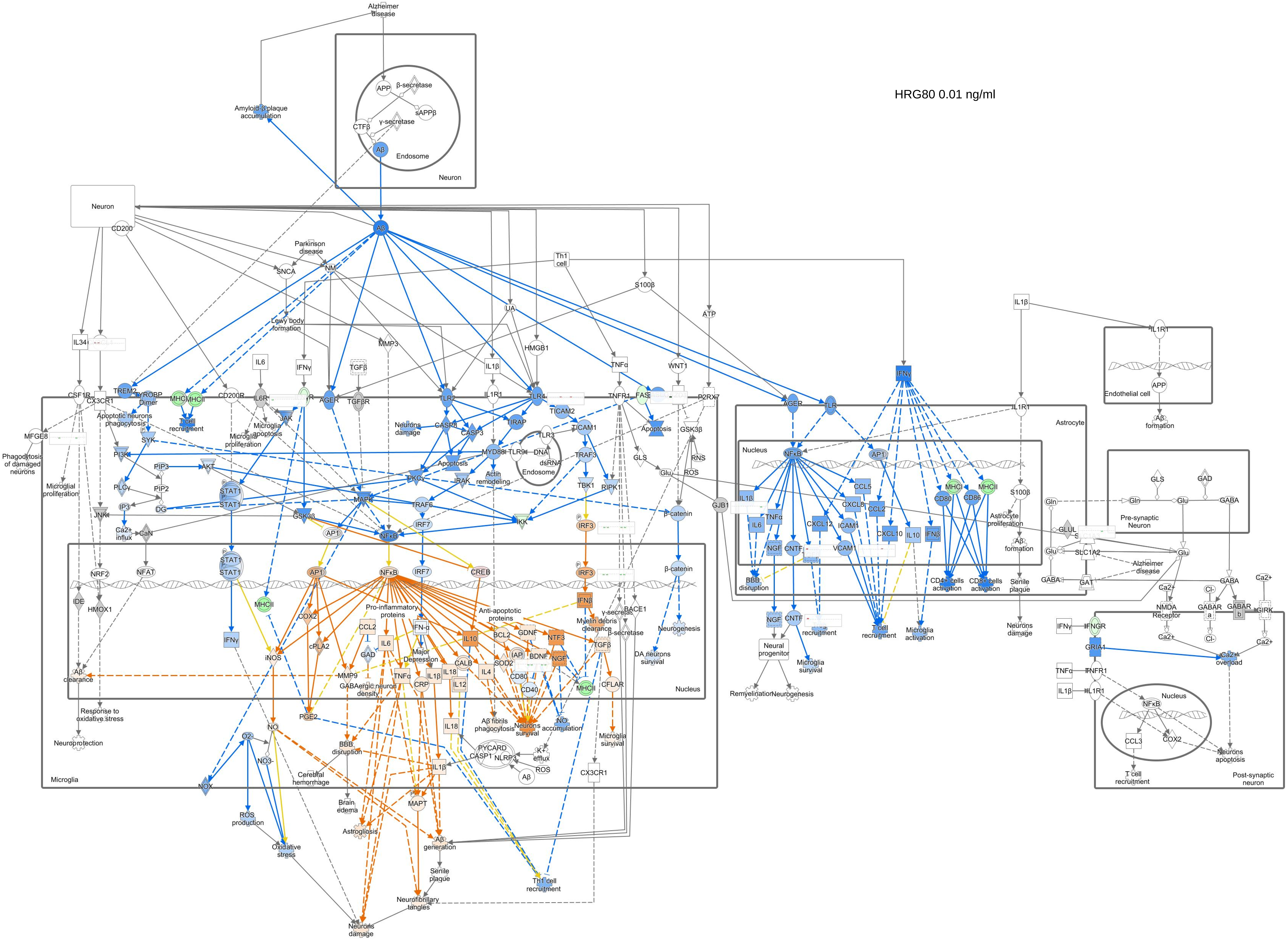

© 2000-2021 QIAGEN. All rights reserved.

Neuroinflammation involves numerous cell types, acts to clear neuronal damage, and plays a key role in maintaining the homeostasis of CNS. Homeostasis can be lost through various regulatory failures, or when humoral immune components cross the blood-brain barrier, causing chronic inflammation with excessive cell and tissue damage, which is associated with neurodegenerative diseases.

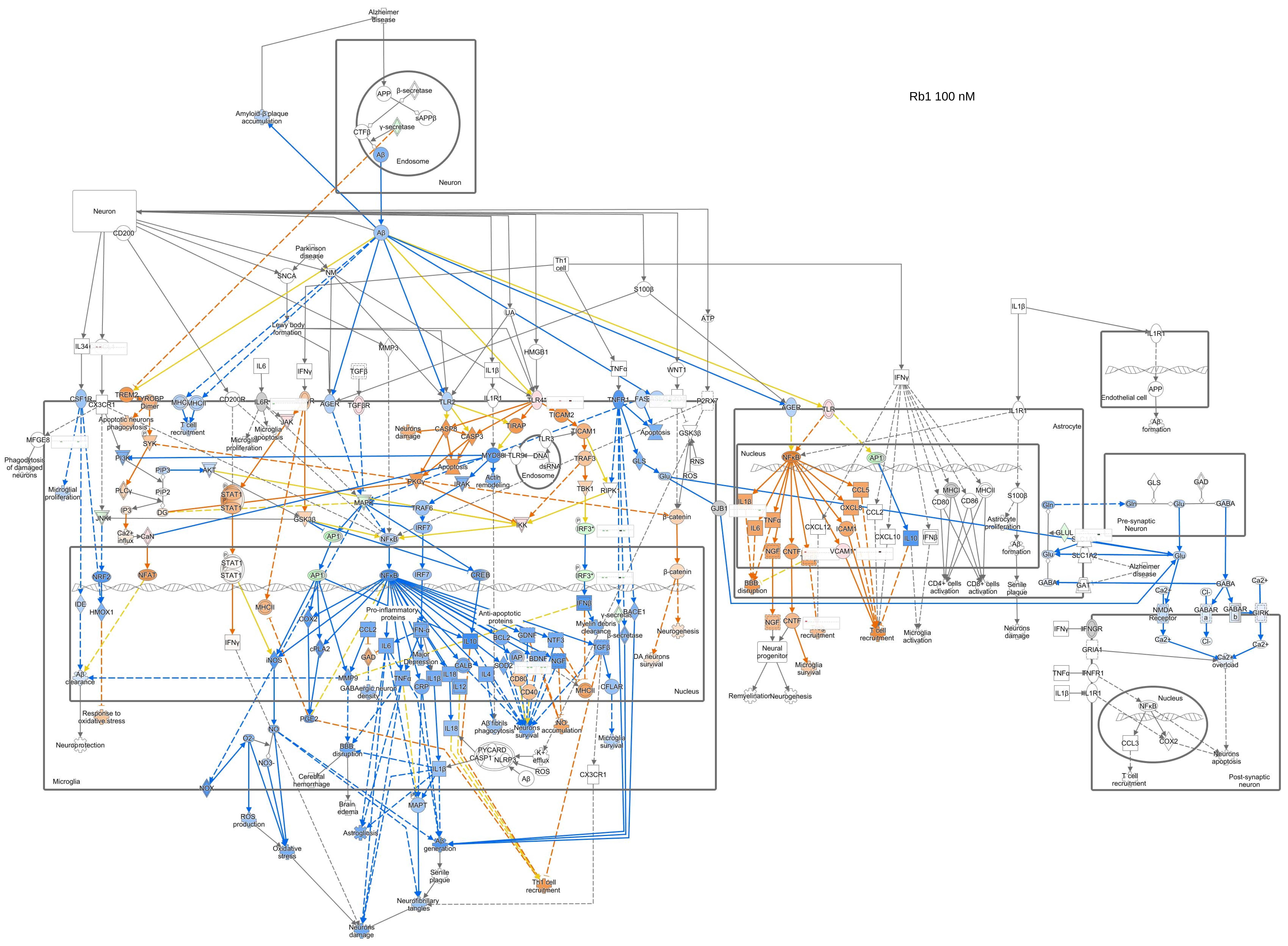

© 2000-2021 QIAGEN. All rights reserved.

Neuroinflammation involves numerous cell types, acts to clear neuronal damage, and plays a key role in maintaining the homeostasis of CNS. Homeostasis can be lost through various regulatory failures, or when humoral immune components cross the blood-brain barrier, causing chronic inflammation with excessive cell and tissue damage, which is associated with neurodegenerative diseases.

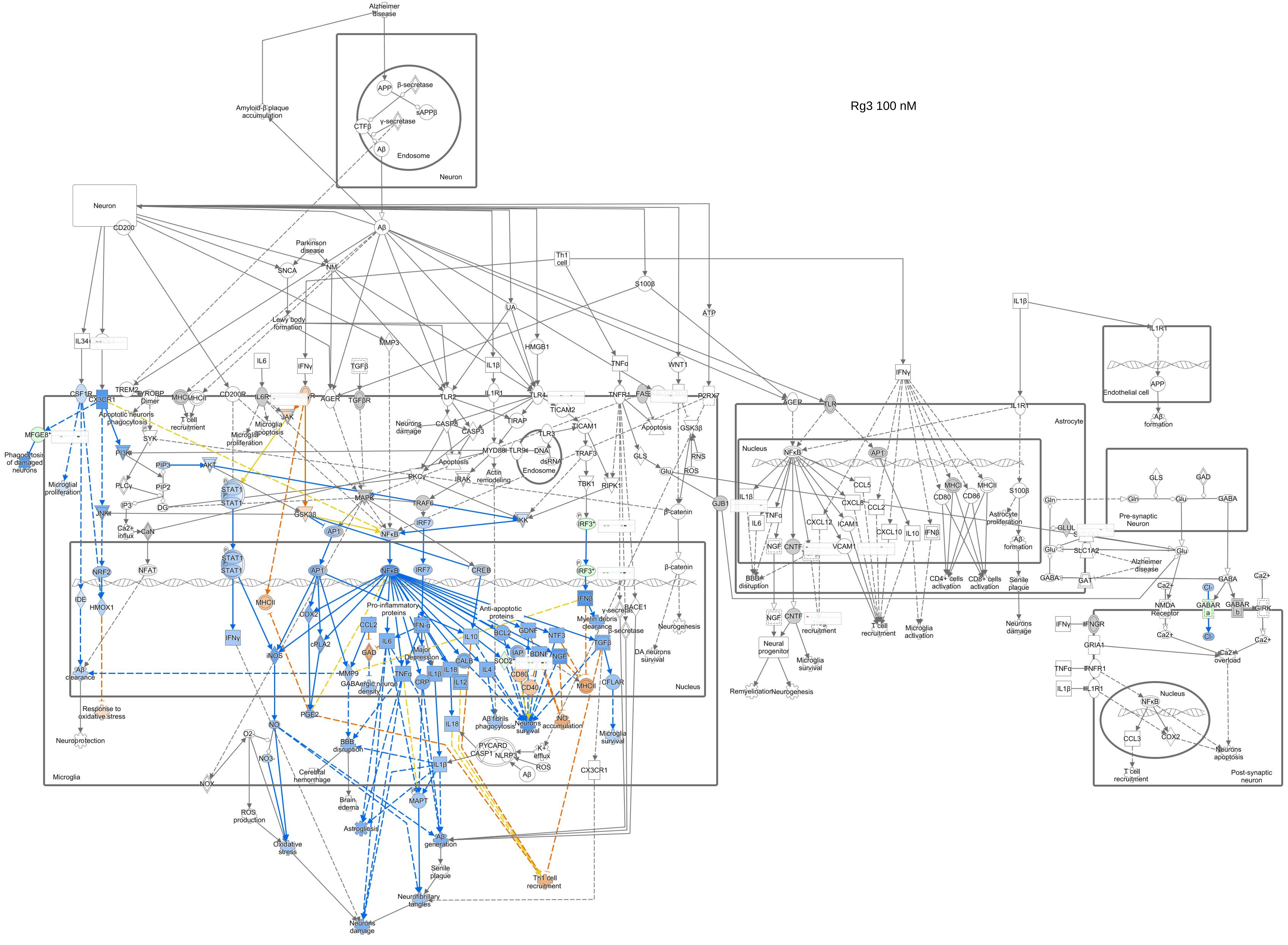

© 2000-2021 QIAGEN. All rights reserved.

Neuroinflammation involves numerous cell types, acts to clear neuronal damage, and plays a key role in maintaining the homeostasis of CNS. Homeostasis can be lost through various regulatory failures, or when humoral immune components cross the blood-brain barrier, causing chronic inflammation with excessive cell and tissue damage, which is associated with neurodegenerative diseases.

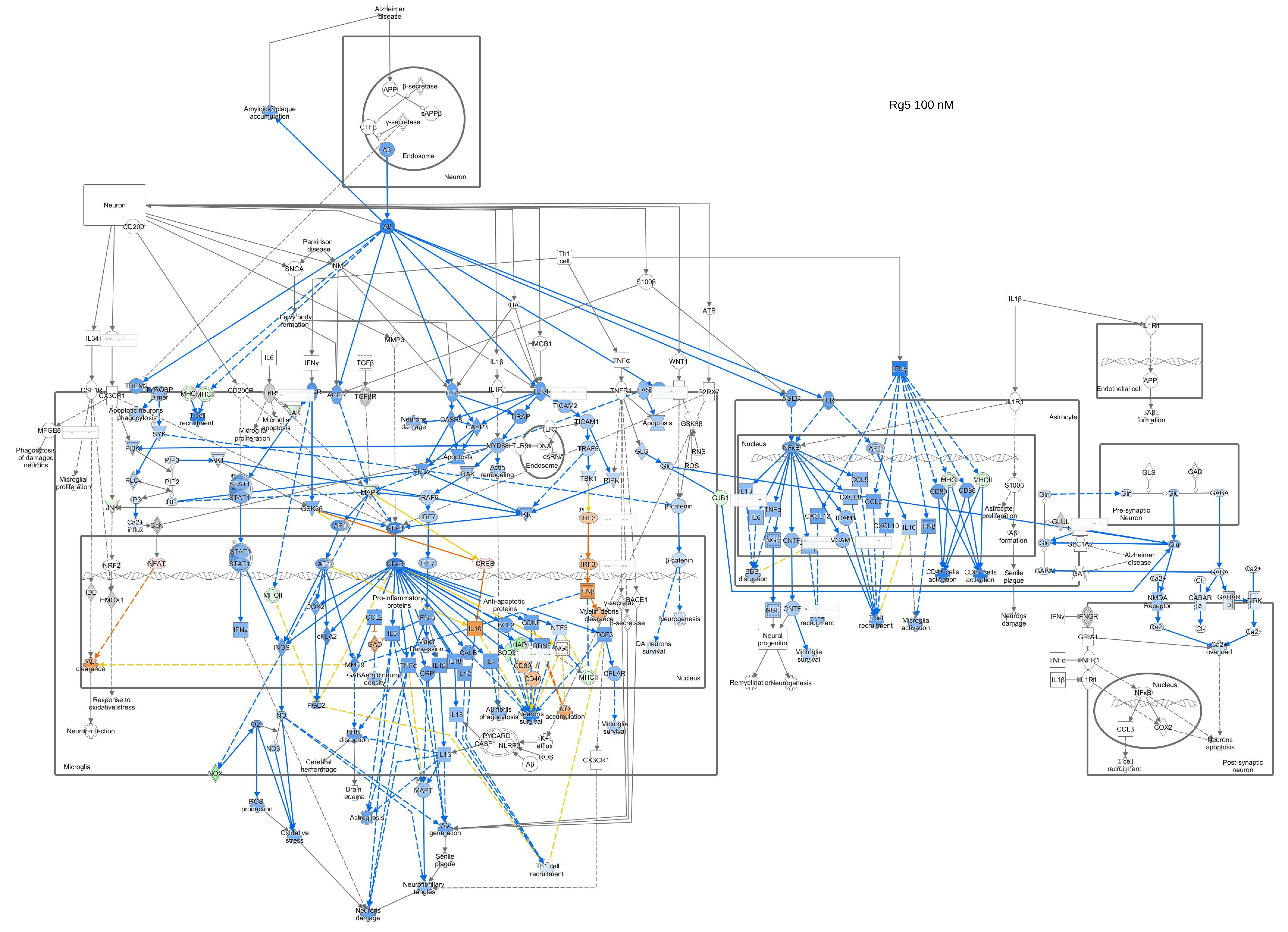

© 2000-2021 QIAGEN. All rights reserved.

Neuroinflammation involves numerous cell types, acts to clear neuronal damage, and plays a key role in maintaining the homeostasis of CNS. Homeostasis can be lost through various regulatory failures, or when humoral immune components cross the blood-brain barrier, causing chronic inflammation with excessive cell and tissue damage, which is associated with neurodegenerative diseases.

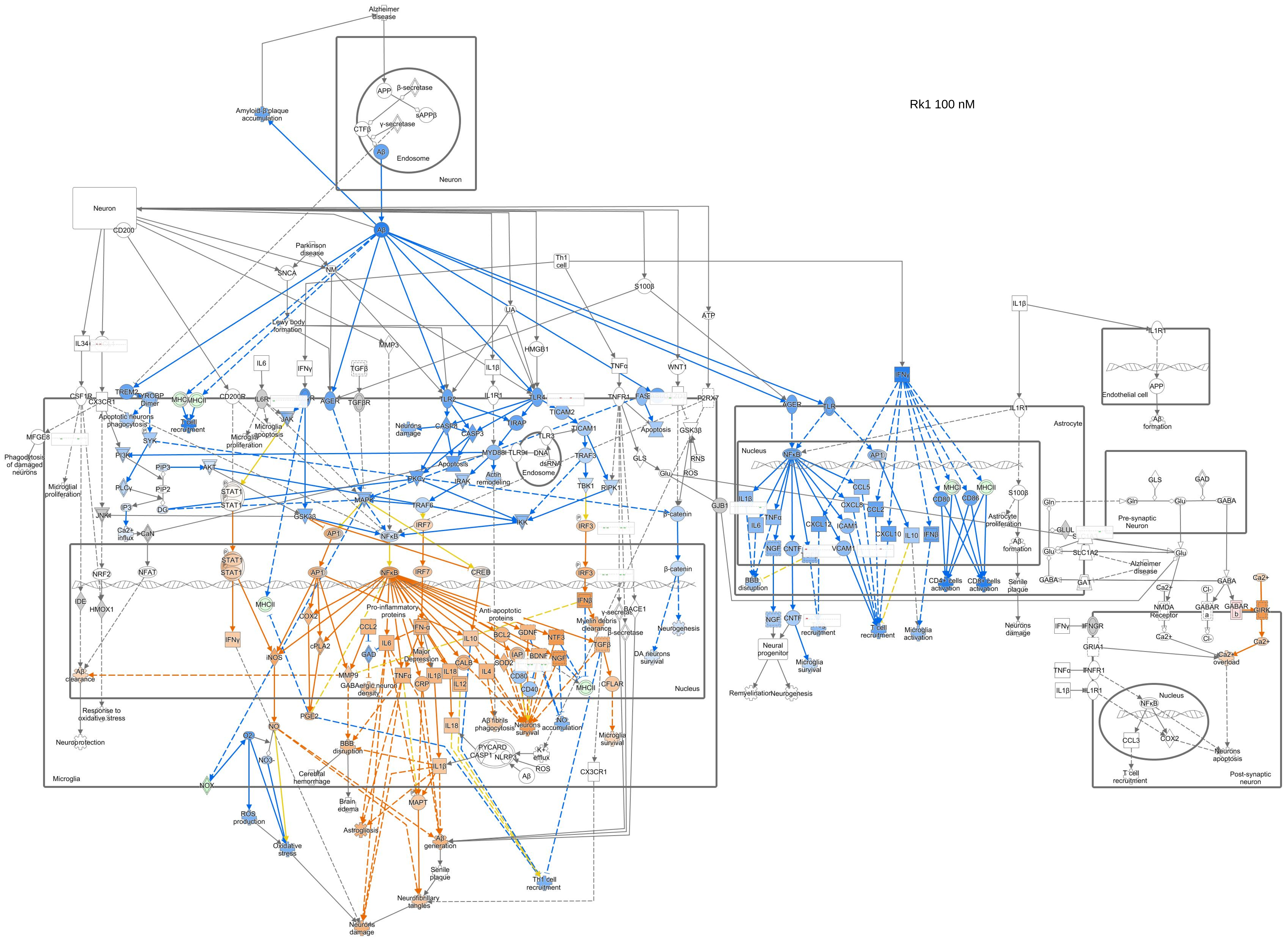

© 2000-2021 QIAGEN. All rights reserved.
