# Supplementary material for: Network Pharmacology of Ginseng (Part II): The Differential Effects of Red Ginseng and Ginsenoside Rg5 in Cancer and Heart Diseases as Determined by Transcriptomics
Source: Pharmaceuticals (Basel). 2021 Sep 30;14(10):1010. doi: 10.3390/ph14101010 (PMC8540751; doi:10.3390/ph14101010)
Supplement: Supplementary file 1 [file pharmaceuticals-14-01010-s001.zip › Supplement 4 Senescence signalling.pdf]

Cellular senescence is an anti-proliferative program that restricts the propagation of cells subjected to different kinds of stress. Senescence has important role in numerous biological processes such maintenance of tissue homeostasis, wound healing, embryonic development and also is implicated in organismal aging, in development of age-related diseases and cancer progression. These detrimental effects are primarily driven by the pro-inflammatory and hyper-secretory phenotype of senescent cells.

WG 10000 ng/ml

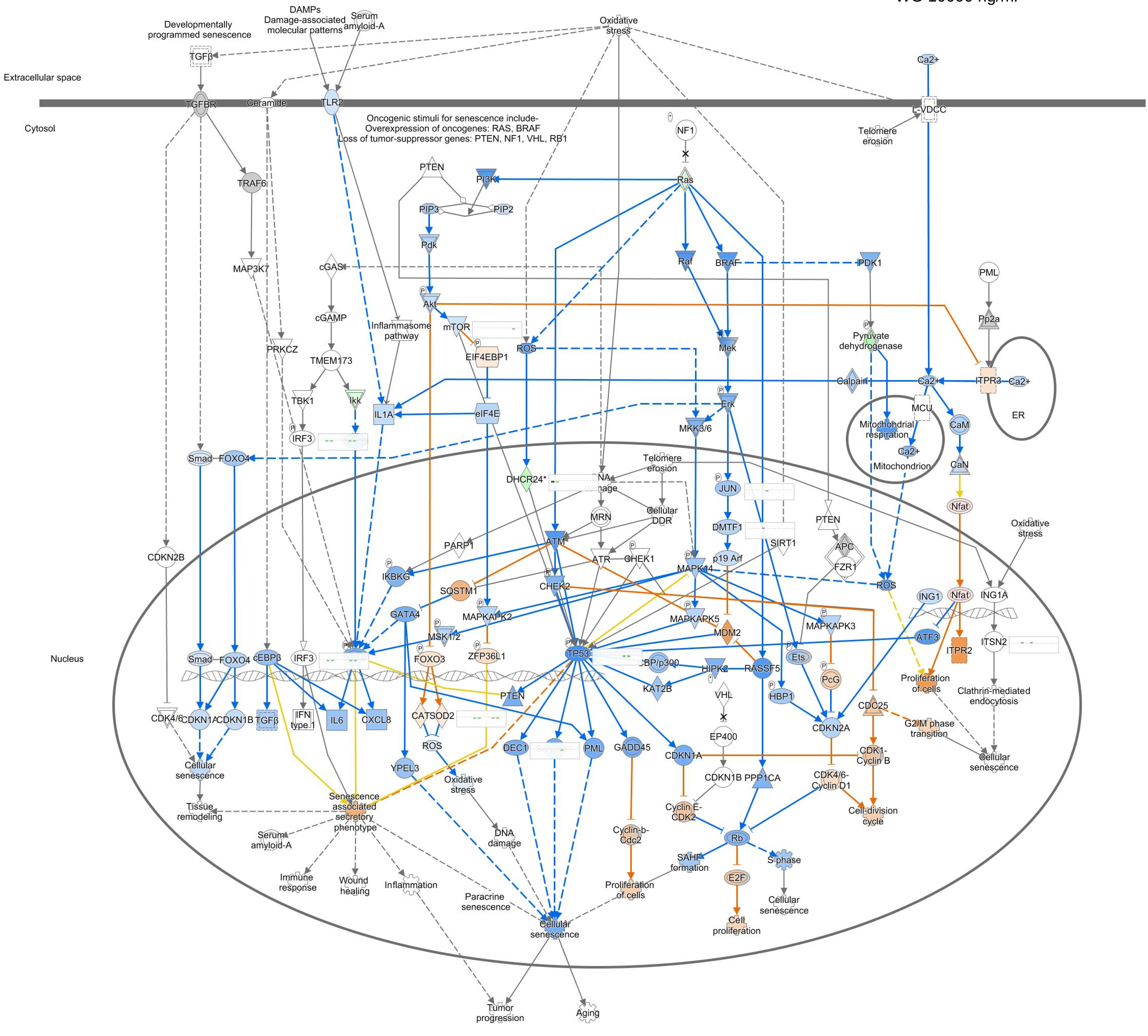

© 2000-2021 QIAGEN. All rights reserved.

Cellular senescence is an anti-proliferative program that restricts the propagation of cells subjected to different kinds of stress.
Senescence has important role in numerous biological processes such maintenance of tissue homeostasis, wound healing, embryonic development and also is implicated in organismal aging, in development of age-related diseases and cancer progression.
These detrimental effects are primarily driven by the pro-inflammatory and hyper-secretory phenotype of senescent cells.

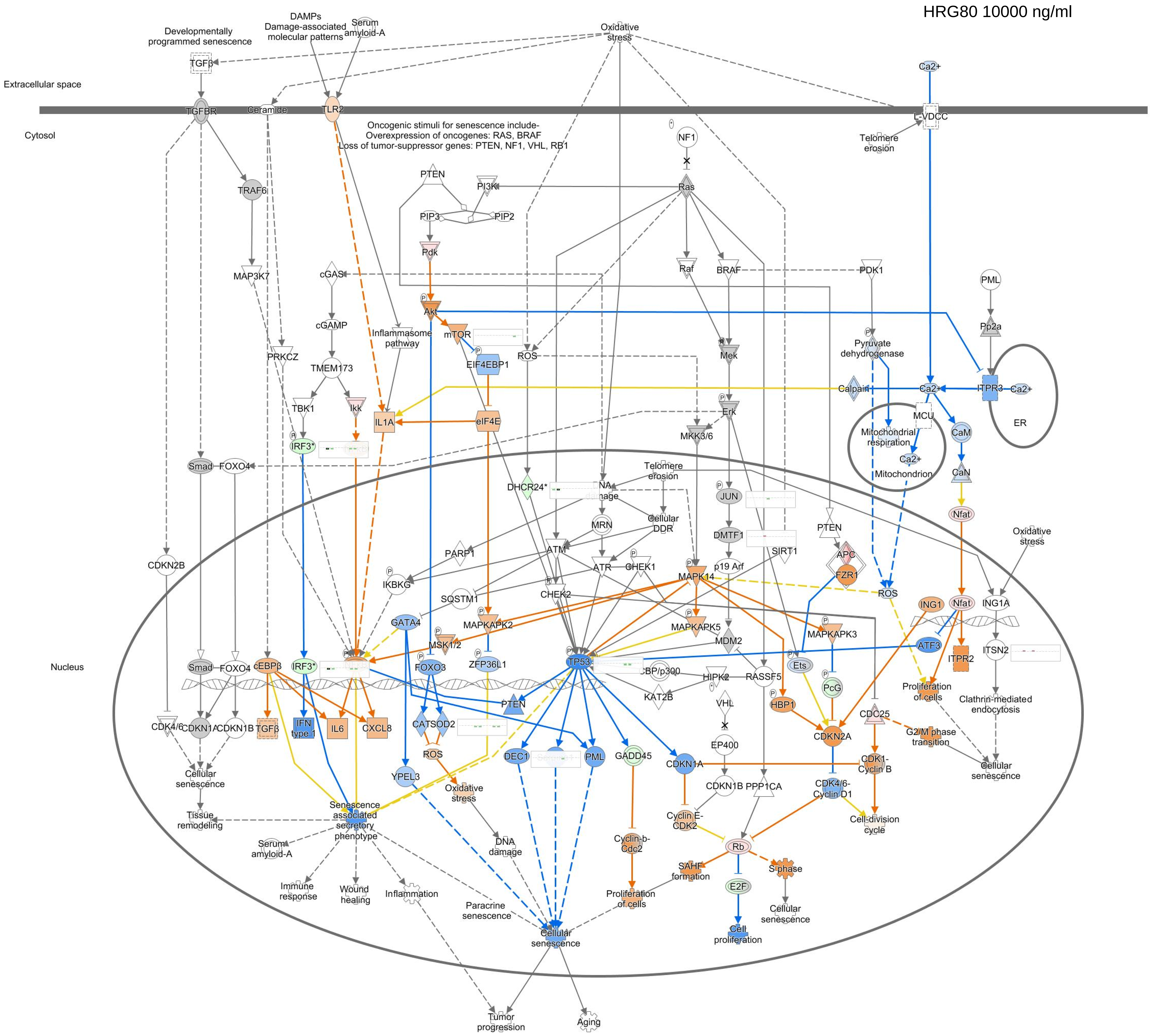

© 2000-2021 QIAGEN. All rights reserved.

Cellular senescence is an anti-proliferative program that restricts the propagation of cells subjected to different kinds of stress.
Senescence has important role in numerous biological processes such maintenance of tissue homeostasis, wound healing, embryonic development
and also is implicated in organismal aging, in development of age-related diseases and cancer progression.
These detrimental effects are primarily driven by the pro-inflammatory and hyper-secretory phenotype of senescent cells.

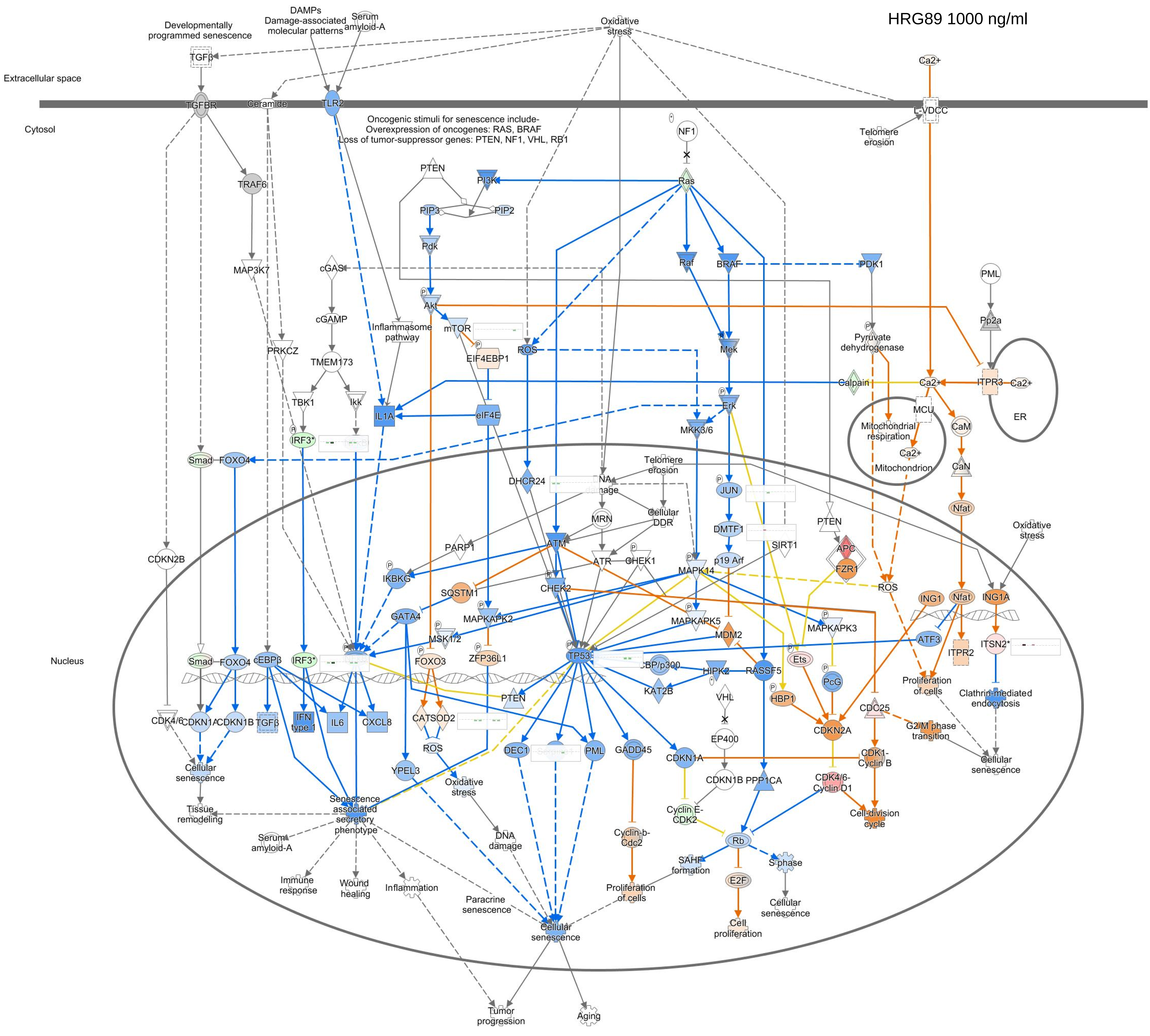

© 2000-2021 QIAGEN. All rights reserved.

Cellular senescence is an anti-proliferative program that restricts the propagation of cells subjected to different kinds of stress.
Senescence has important role in numerous biological processes such maintenance of tissue homeostasis, wound healing, embryonic development and also is implicated in organismal aging, in development of age-related diseases and cancer progression.
These detrimental effects are primarily driven by the pro-inflammatory and hyper-secretory phenotype of senescent cells.

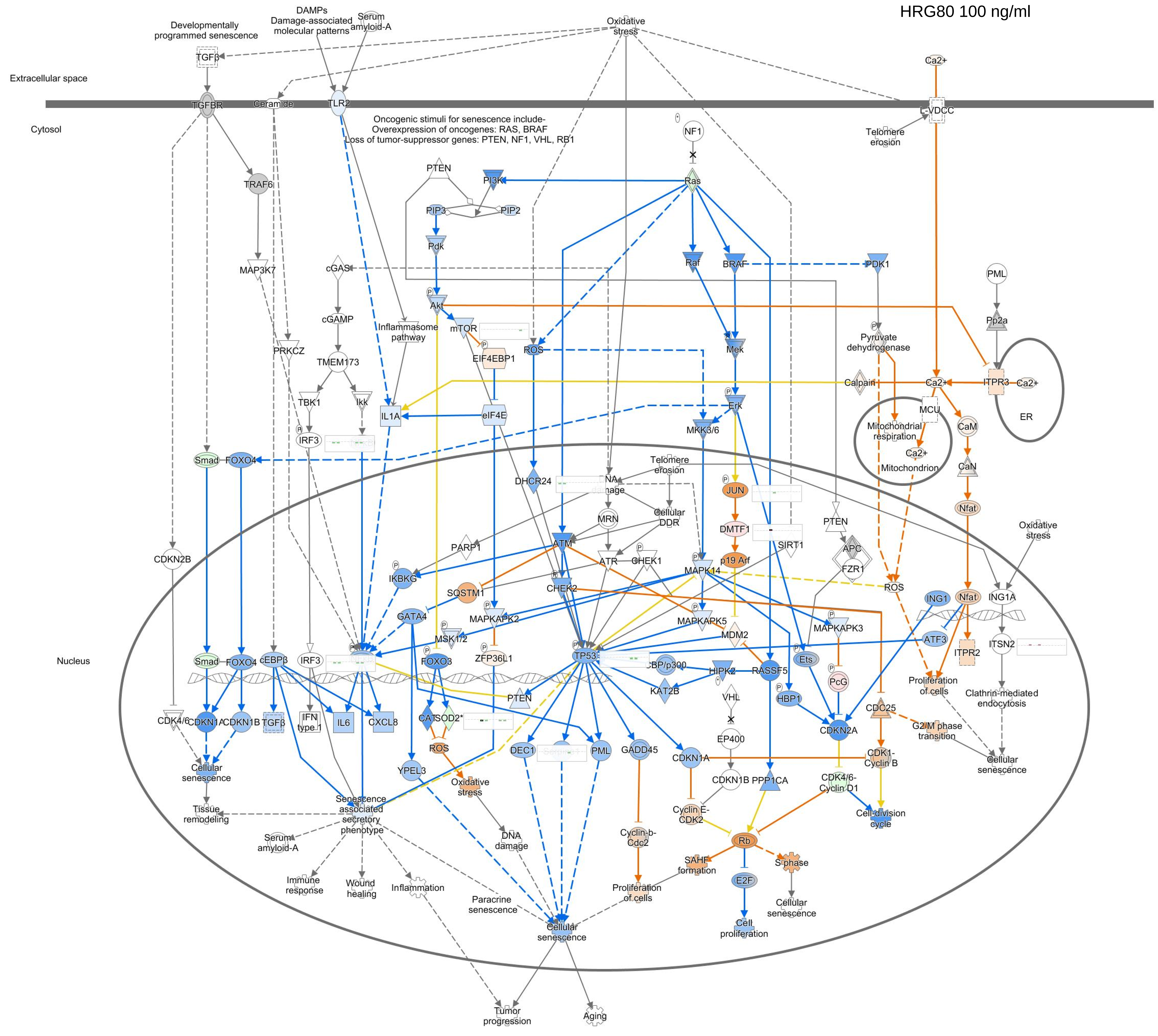

© 2000-2021 QIAGEN. All rights reserved.

Cellular senescence is an anti-proliferative program that restricts the propagation of cells subjected to different kinds of stress.
Senescence has important role in numerous biological processes such maintenance of tissue homeostasis, wound healing, embryonic development and also is implicated in organismal aging, in development of age-related diseases and cancer progression.
These detrimental effects are primarily driven by the pro-inflammatory and hyper-secretory phenotype of senescent cells.

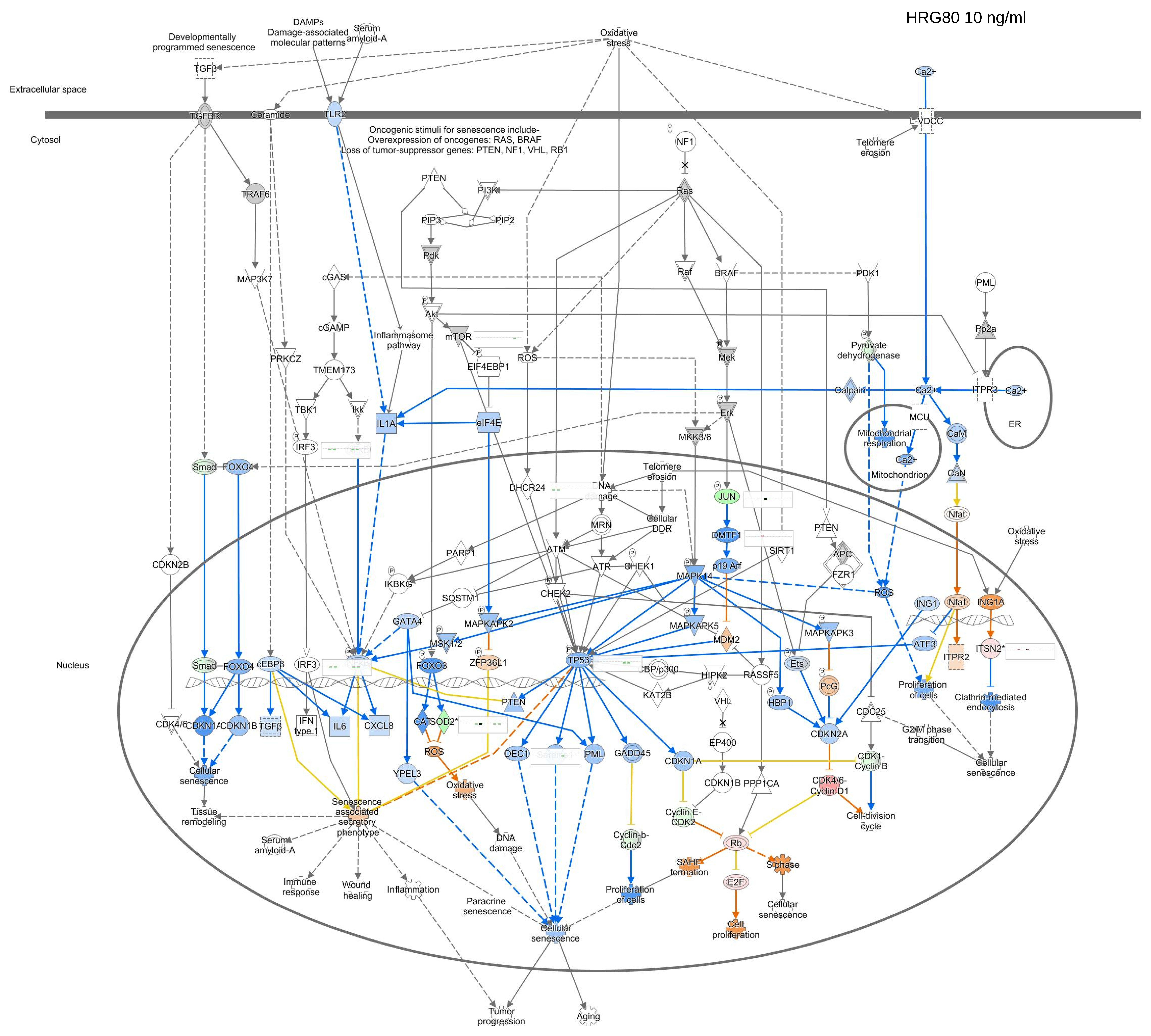

© 2000-2021 QIAGEN. All rights reserved.

Cellular senescence is an anti-proliferative program that restricts the propagation of cells subjected to different kinds of stress. Senescence has important role in numerous biological processes such maintenance of tissue homeostasis, wound healing, embryonic development and also is implicated in organismal aging, in development of age-related diseases and cancer progression. These detrimental effects are primarily driven by the pro-inflammatory and hyper-secretory phenotype of senescent cells.

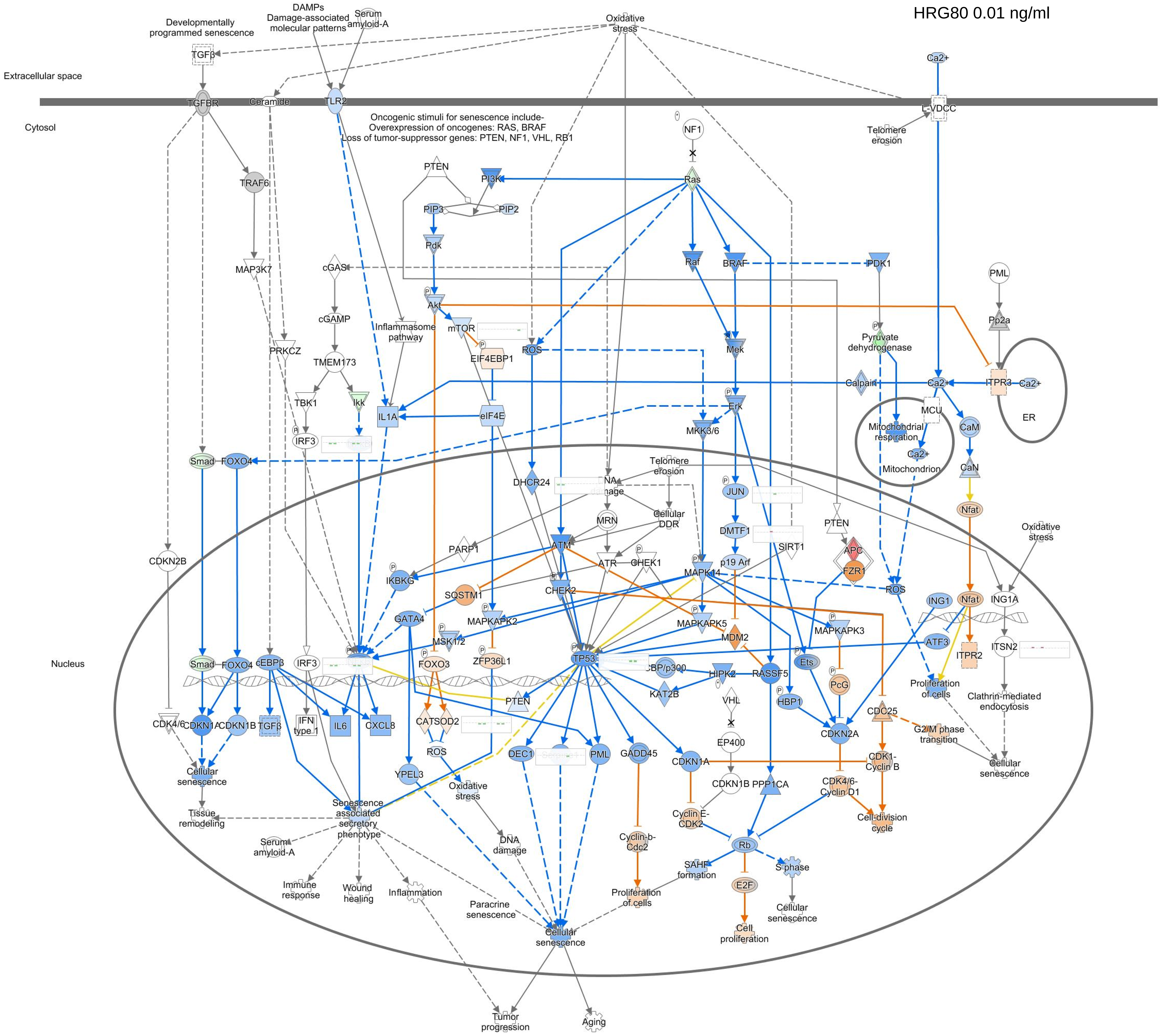

© 2000-2021 QIAGEN. All rights reserved.

Cellular senescence is an anti-proliferative program that restricts the propagation of cells subjected to different kinds of stress.
Senescence has important role in numerous biological processes such maintenance of tissue homeostasis, wound healing, embryonic development and also is implicated in organismal aging, in development of age-related diseases and cancer progression.
These detrimental effects are primarily driven by the pro-inflammatory and hyper-secretory phenotype of senescent cells.

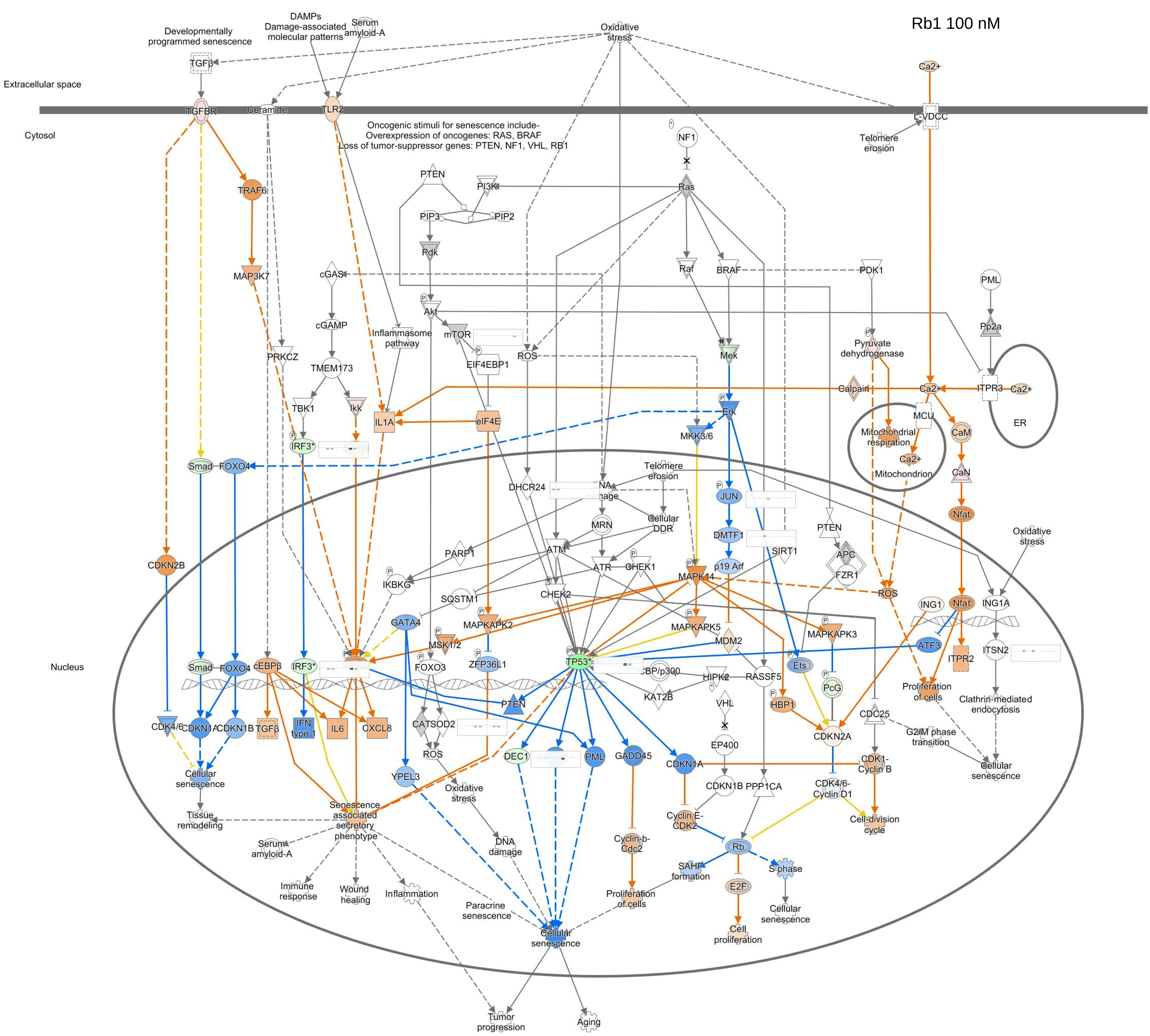

© 2000-2021 QIAGEN. All rights reserved.

Cellular senescence is an anti-proliferative program that restricts the propagation of cells subjected to different kinds of stress.
Senescence has important role in numerous biological processes such maintenance of tissue homeostasis, wound healing, embryonic development and also is implicated in organismal aging, in development of age-related diseases and cancer progression.
These detrimental effects are primarily driven by the pro-inflammatory and hyper-secretory phenotype of senescent cells.

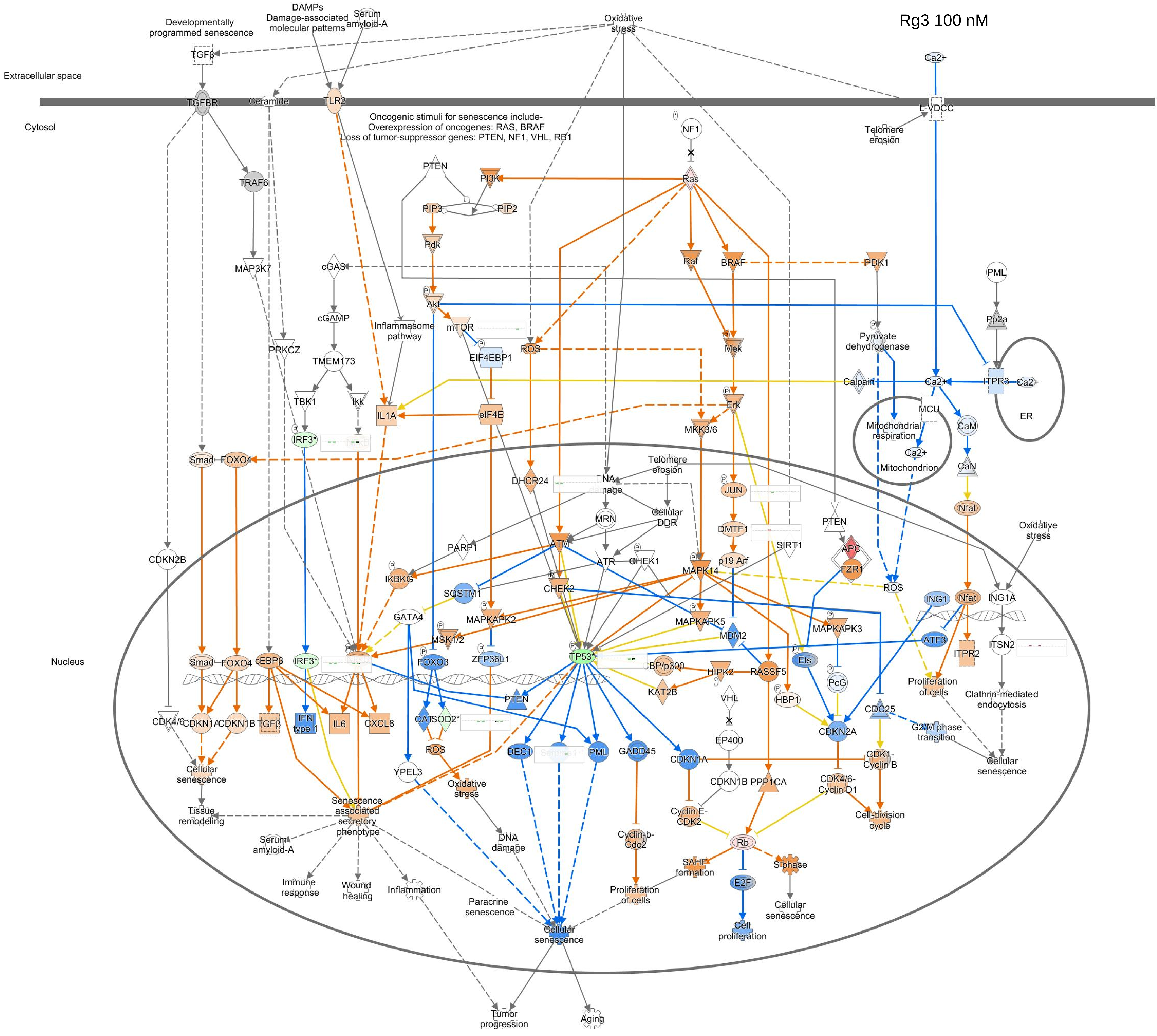

© 2000-2021 QIAGEN. All rights reserved.

Cellular senescence is an anti-proliferative program that restricts the propagation of cells subjected to different kinds of stress. Senescence has important role in numerous biological processes such maintenance of tissue homeostasis, wound healing, embryonic development and also is implicated in organismal aging, in development of age-related diseases and cancer progression. These detrimental effects are primarily driven by the pro-inflammatory and hyper-secretory phenotype of senescent cells.

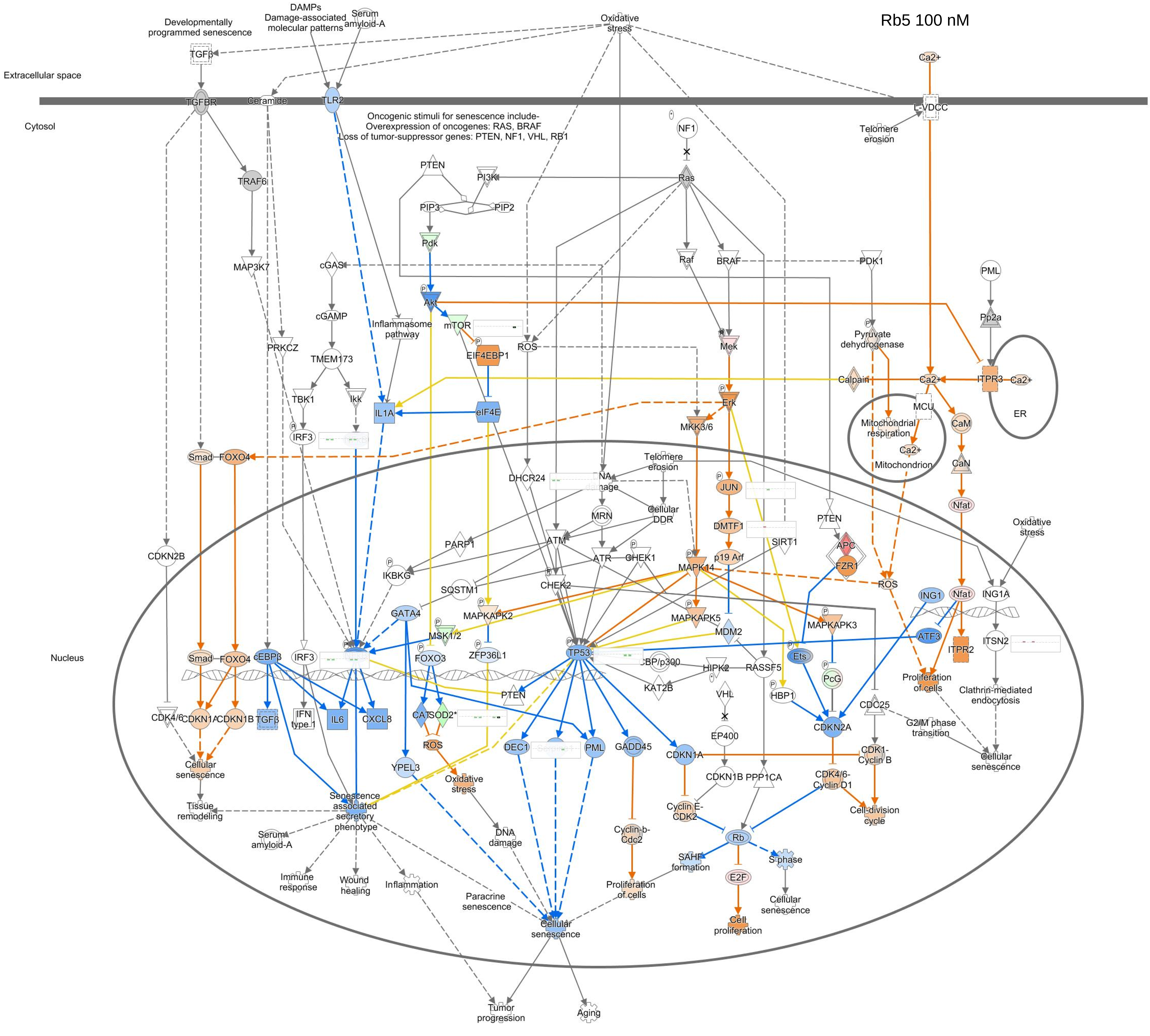

© 2000-2021 QIAGEN. All rights reserved.

Cellular senescence is an anti-proliferative program that restricts the propagation of cells subjected to different kinds of stress. Senescence has important role in numerous biological processes such maintenance of tissue homeostasis, wound healing, embryonic development and also is implicated in organismal aging, in development of age-related diseases and cancer progression. These detrimental effects are primarily driven by the pro-inflammatory and hyper-secretory phenotype of senescent cells.

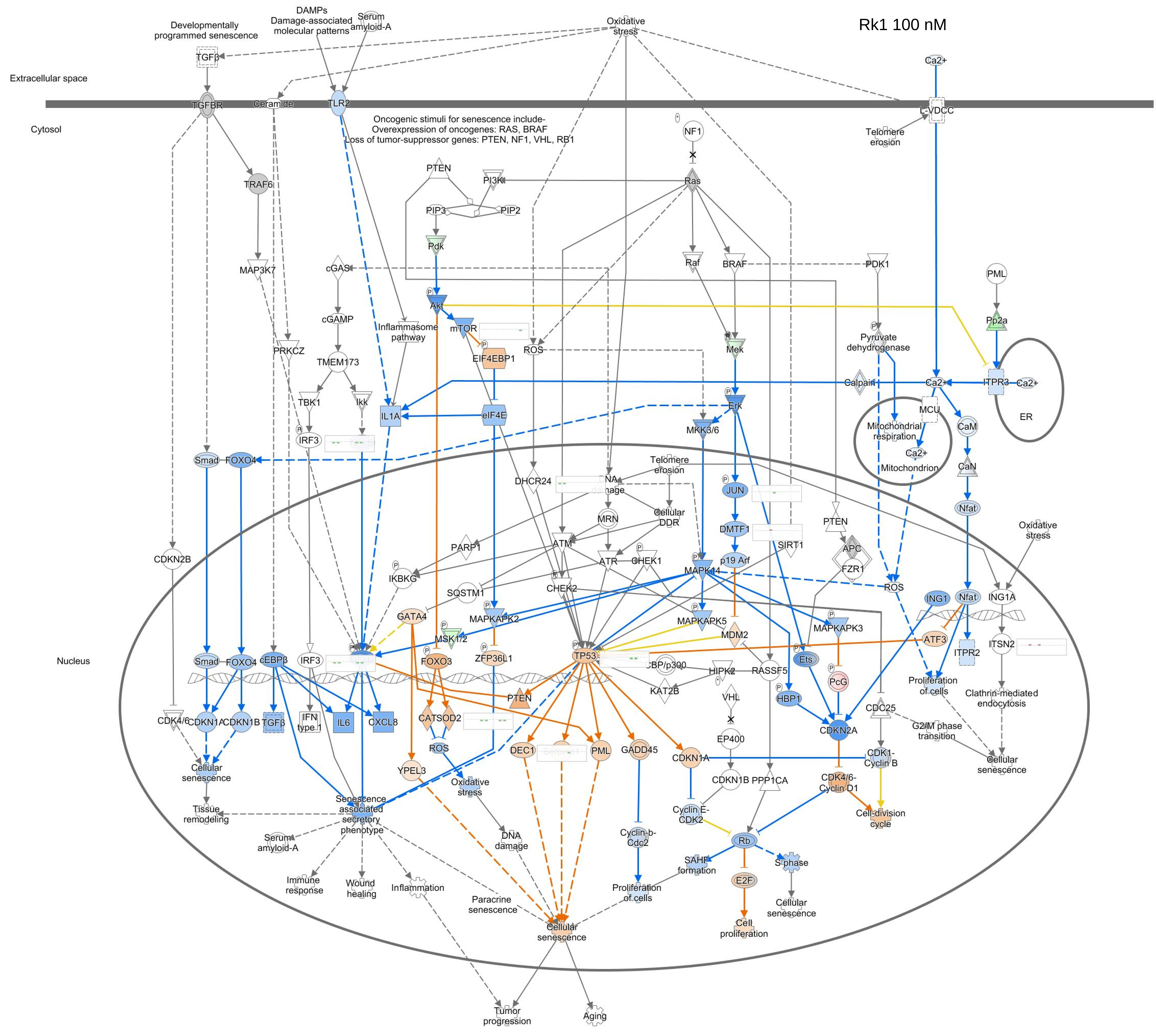

© 2000-2021 QIAGEN. All rights reserved.
