# Supplementary material for: Network Pharmacology of Ginseng (Part II): The Differential Effects of Red Ginseng and Ginsenoside Rg5 in Cancer and Heart Diseases as Determined by Transcriptomics
Source: Pharmaceuticals (Basel). 2021 Sep 30;14(10):1010. doi: 10.3390/ph14101010 (PMC8540751; doi:10.3390/ph14101010)
Supplement: Supplementary file 1 [file pharmaceuticals-14-01010-s001.zip › Supplement 6 Sirtuin signalling.pdf]

Sirtuins are class III histone deacetylase enzymes that use NAD+ as a co-substrate for their enzymatic activities.
In mammals, there are 7 sirtuin members (SIRT1-7), which play important roles in aging, metabolism, cancer, inflammation,
DNA repair and cellular responses to stress.

WG 10000 ng/ml

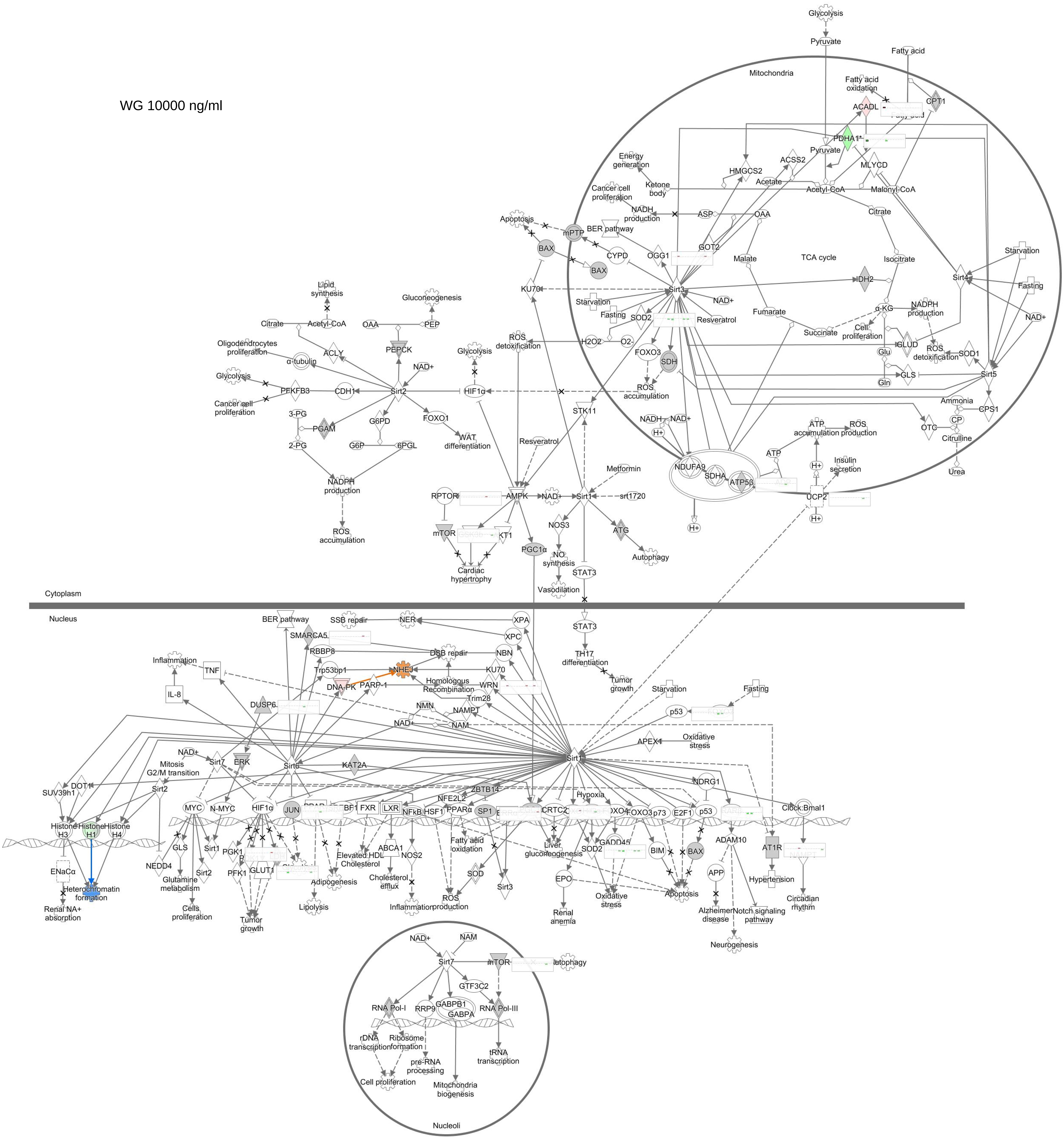

© 2000-2021 QIAGEN. All rights reserved.

Sirtuins are class III histone deacetylase enzymes that use NAD+ as a co-substrate for their enzymatic activities.
In mammals, there are 7 sirtuin members (SIRT1-7), which play important roles in aging, metabolism, cancer, inflammation,
DNA repair and cellular responses to stress.

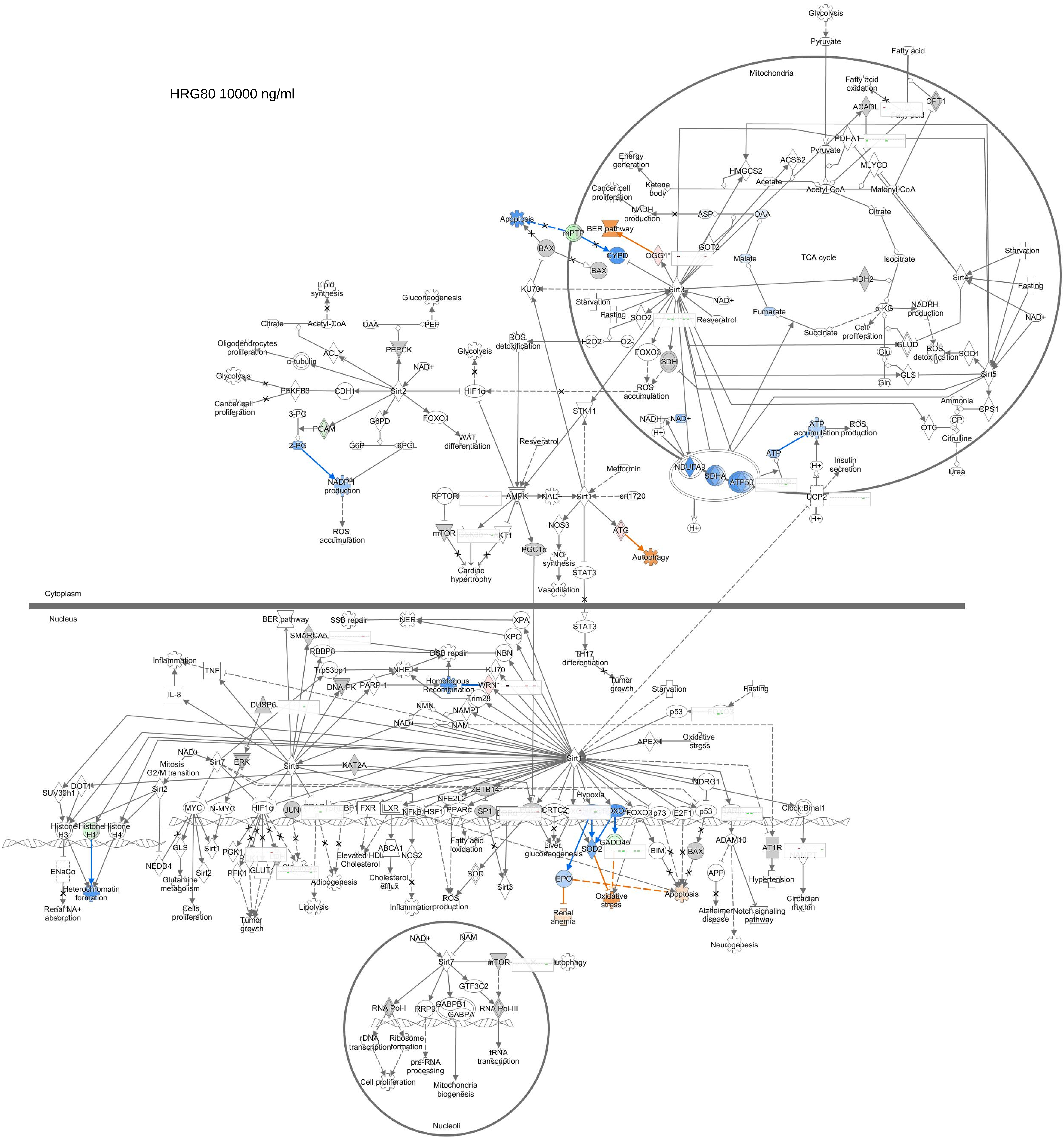

© 2000-2021 QIAGEN. All rights reserved.

Sirtuins are class III histone deacetylase enzymes that use NAD+ as a co-substrate for their enzymatic activities.
In mammals, there are 7 sirtuin members (SIRT1-7), which play important roles in aging, metabolism, cancer, inflammation,
DNA repair and cellular responses to stress.

HRG80 1000 ng/ml

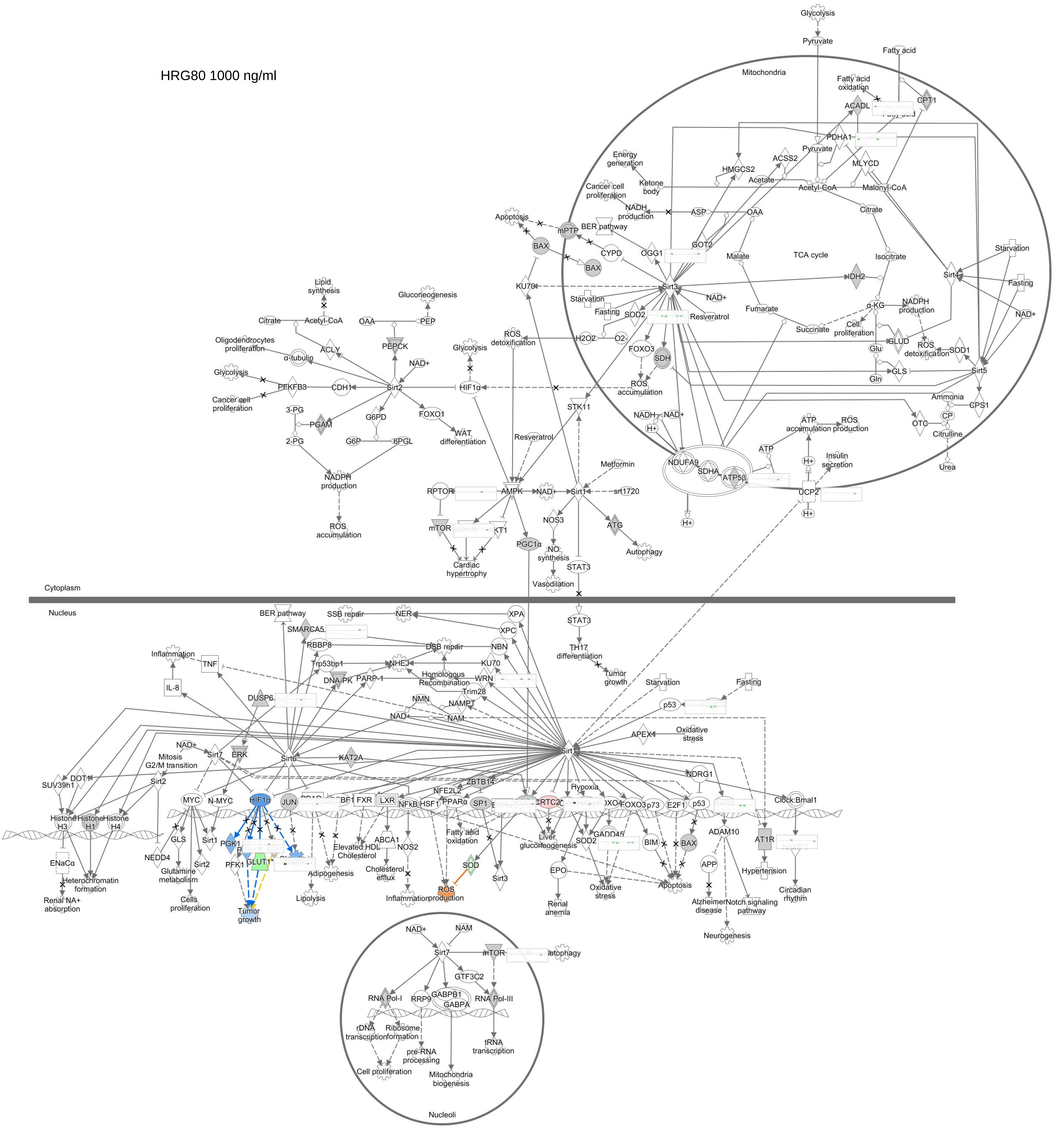

© 2000-2021 QIAGEN. All rights reserved.

Sirtuins are class III histone deacetylase enzymes that use NAD+ as a co-substrate for their enzymatic activities.
In mammals, there are 7 sirtuin members (SIRT1-7), which play important roles in aging, metabolism, cancer, inflammation,
DNA repair and cellular responses to stress.

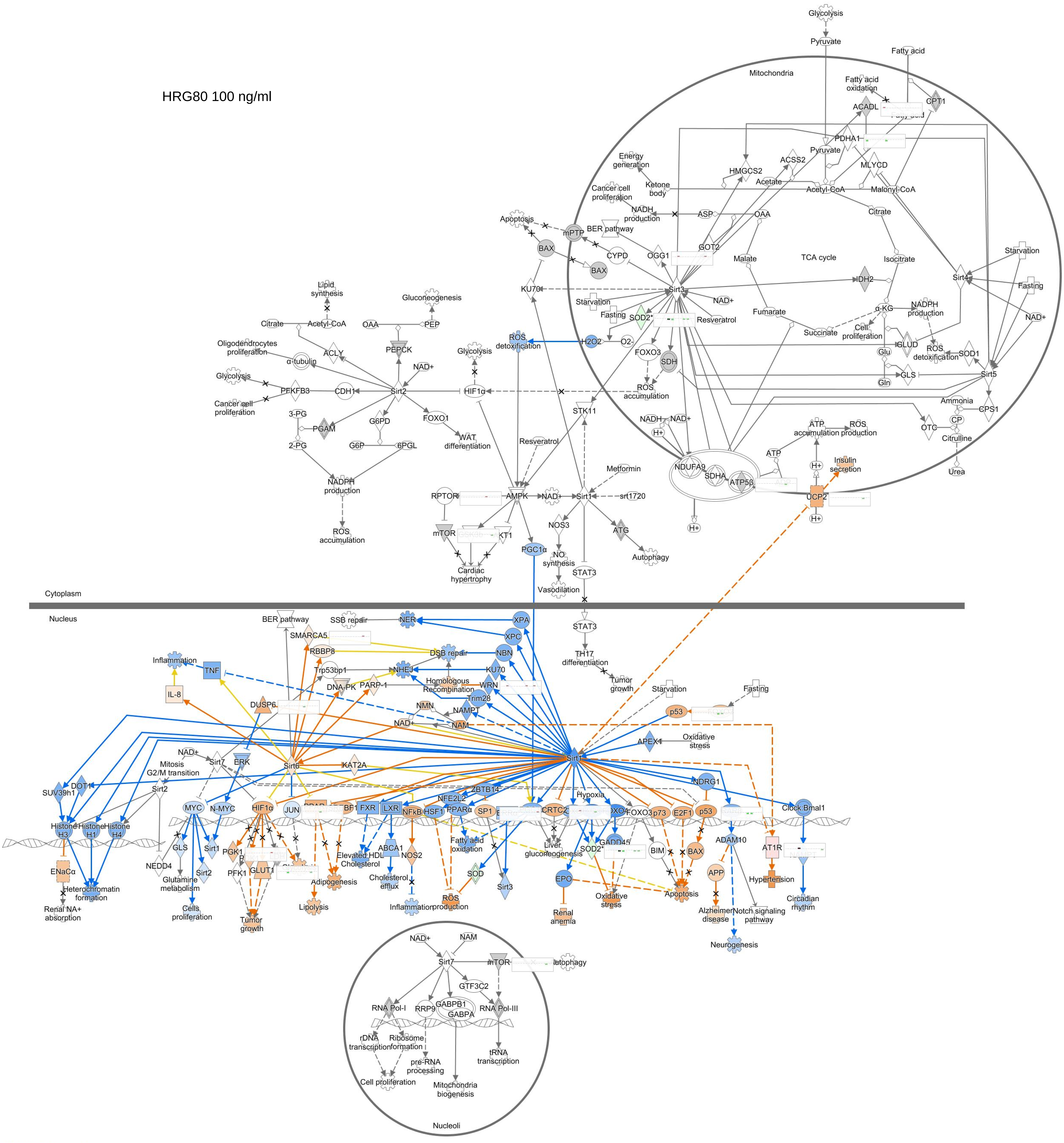

© 2000-2021 QIAGEN. All rights reserved.

Sirtuins are class III histone deacetylase enzymes that use NAD+ as a co-substrate for their enzymatic activities.
In mammals, there are 7 sirtuin members (SIRT1-7), which play important roles in aging, metabolism, cancer, inflammation, DNA repair and cellular responses to stress.

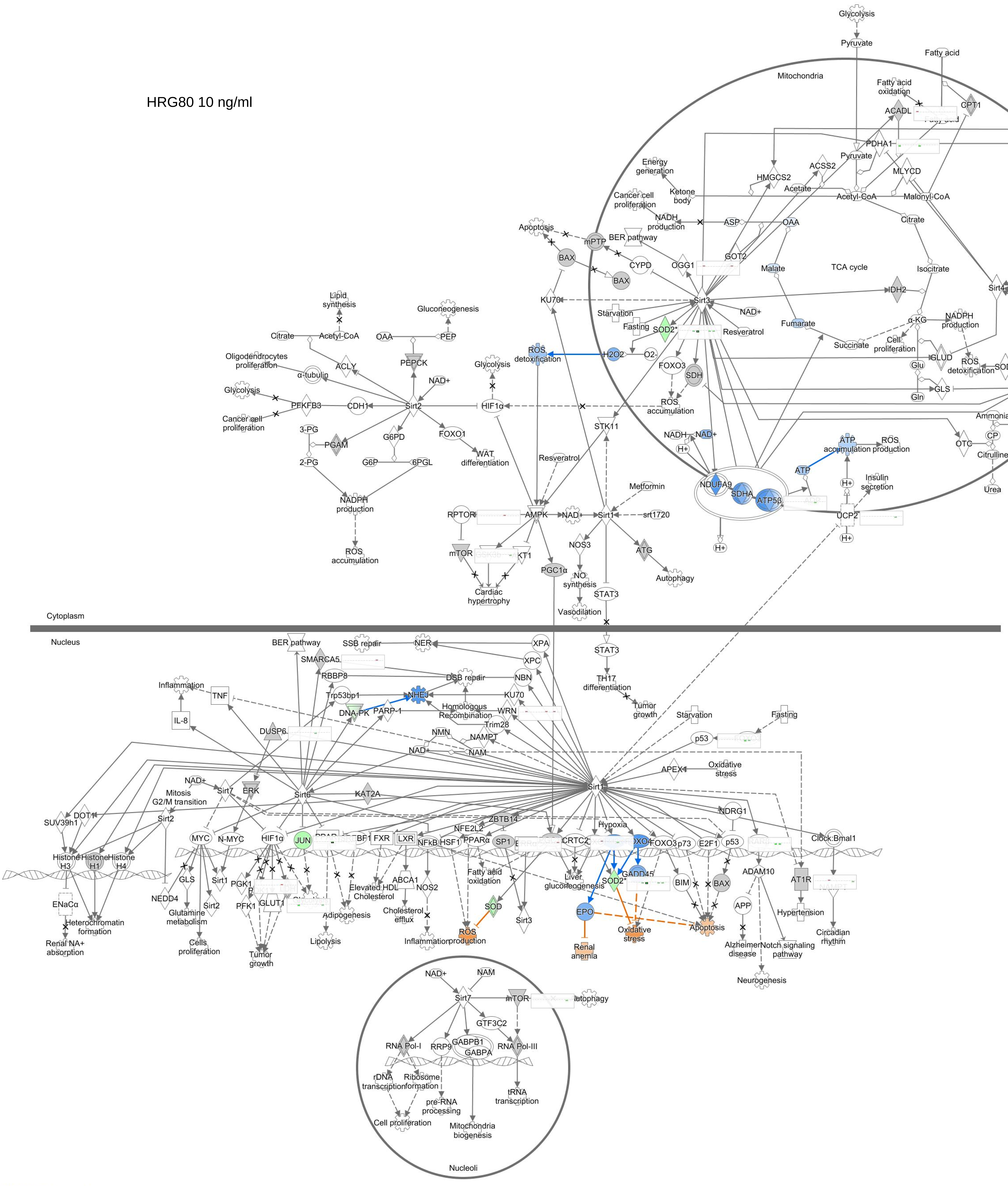

© 2000-2021 QIAGEN. All rights reserved.

Sirtuins are class III histone deacetylase enzymes that use NAD+ as a co-substrate for their enzymatic activities.
In mammals, there are 7 sirtuin members (SIRT1-7), which play important roles in aging, metabolism, cancer, inflammation,
DNA repair and cellular responses to stress.

HRG80 0.01 ng/ml

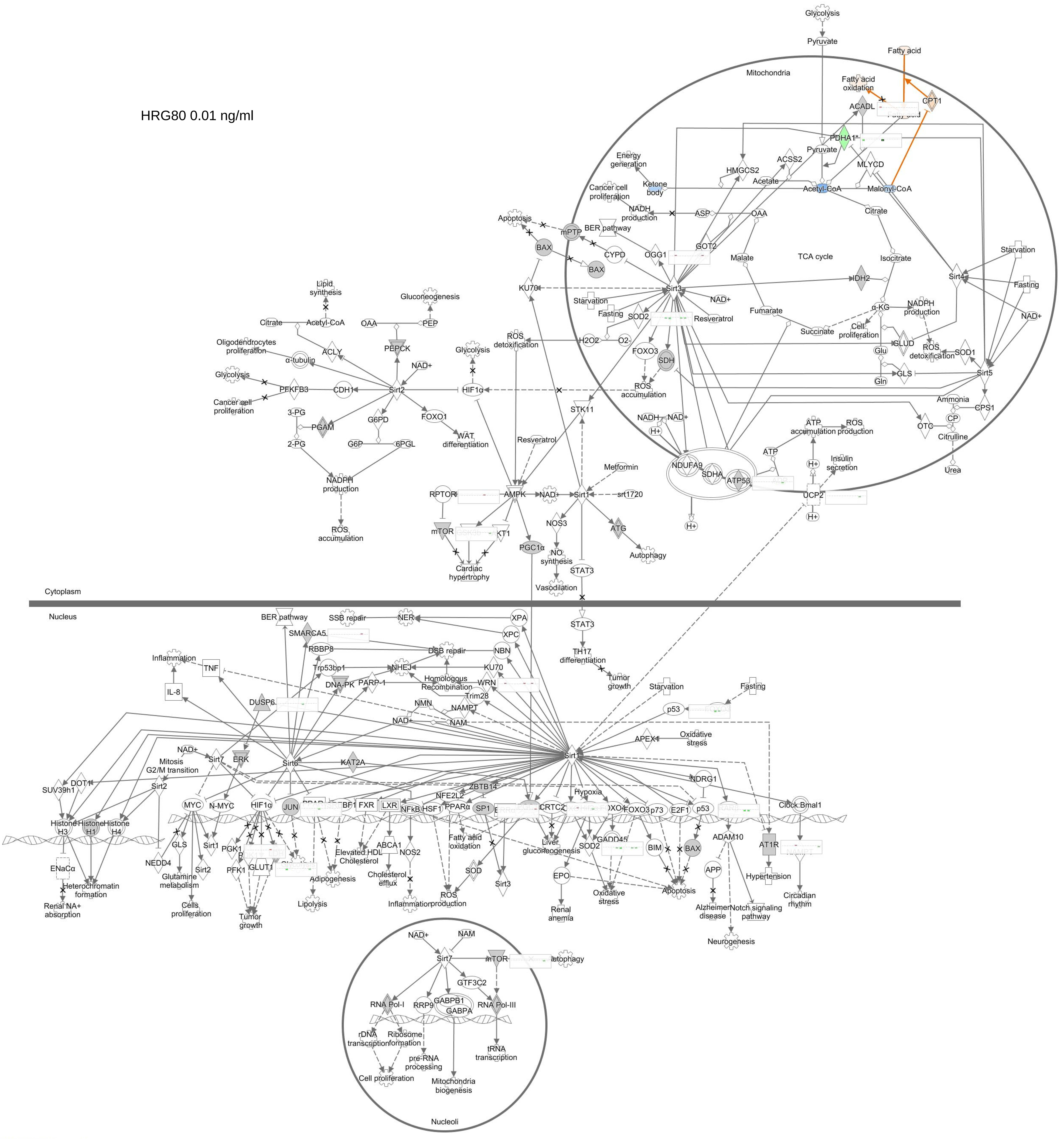

© 2000-2021 QIAGEN. All rights reserved.

Sirtuins are class III histone deacetylase enzymes that use NAD+ as a co-substrate for their enzymatic activities.
In mammals, there are 7 sirtuin members (SIRT1-7), which play important roles in aging, metabolism, cancer, inflammation,
DNA repair and cellular responses to stress.

Rb1 100 nM

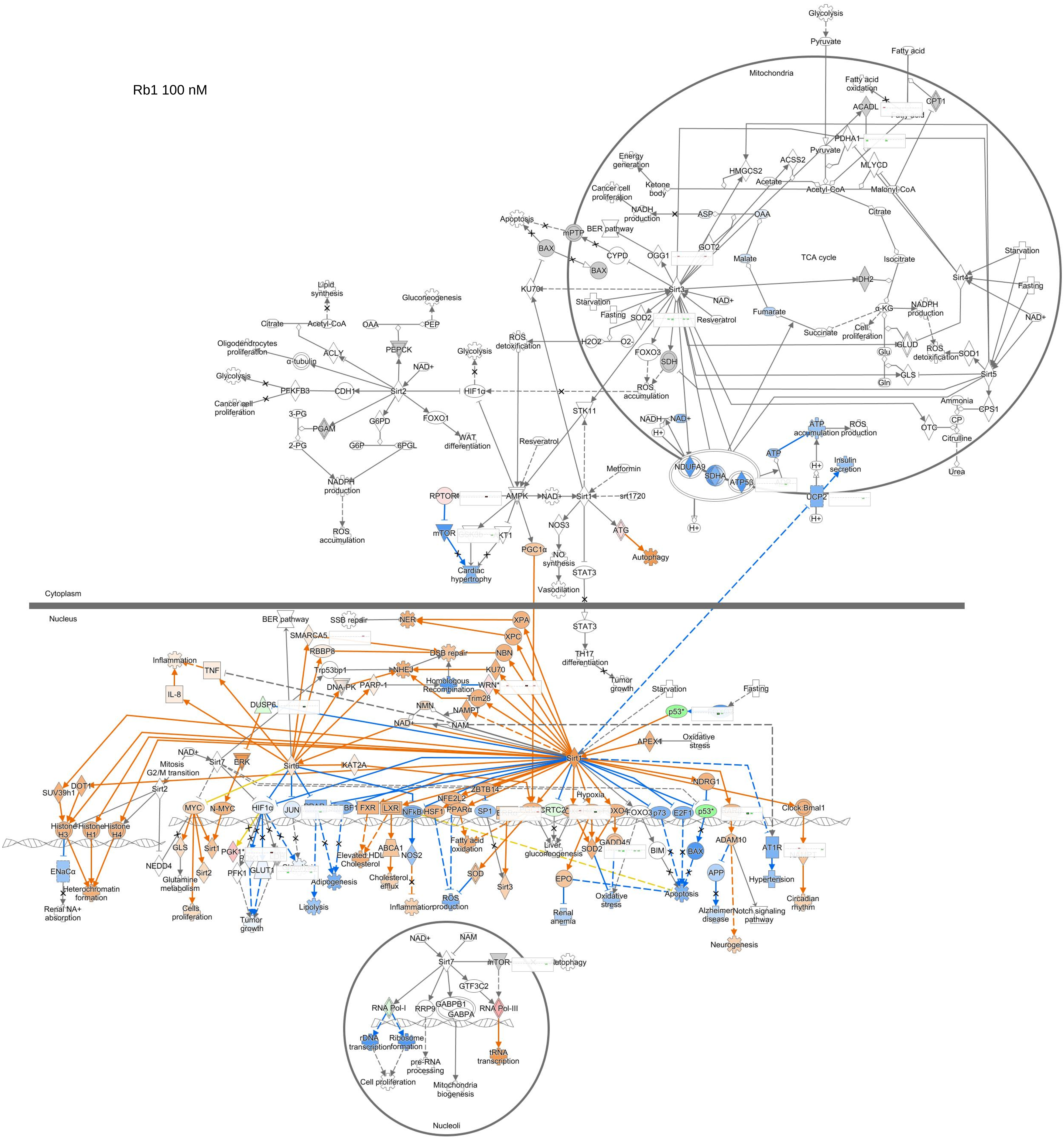

© 2000-2021 QIAGEN. All rights reserved.

Sirtuins are class III histone deacetylase enzymes that use NAD+ as a co-substrate for their enzymatic activities.
In mammals, there are 7 sirtuin members (SIRT1-7), which play important roles in aging, metabolism, cancer, inflammation,
DNA repair and cellular responses to stress.

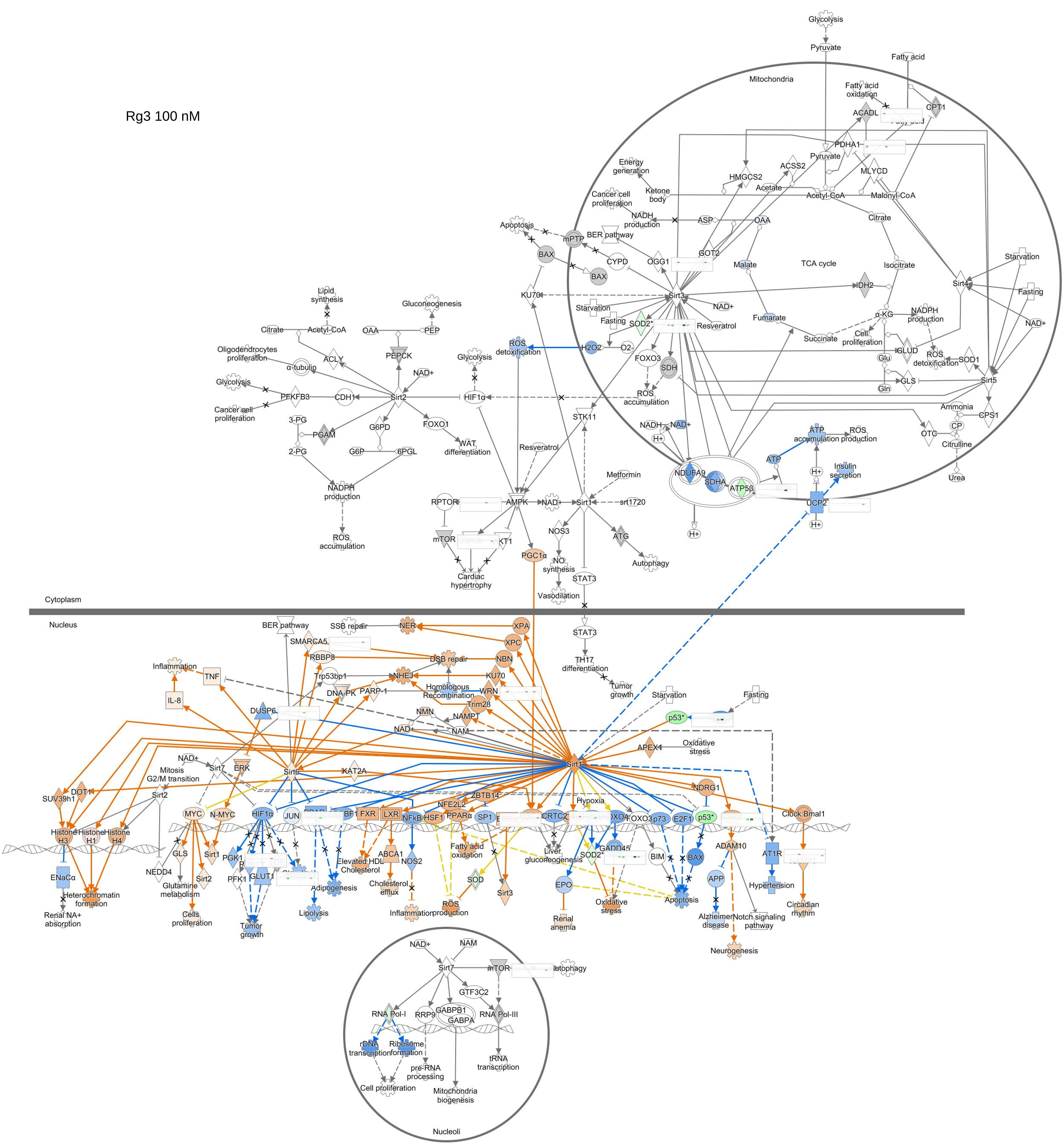

© 2000-2021 QIAGEN. All rights reserved.

Sirtuins are class III histone deacetylase enzymes that use NAD+ as a co-substrate for their enzymatic activities.
In mammals, there are 7 sirtuin members (SIRT1-7), which play important roles in aging, metabolism, cancer, inflammation,
DNA repair and cellular responses to stress.

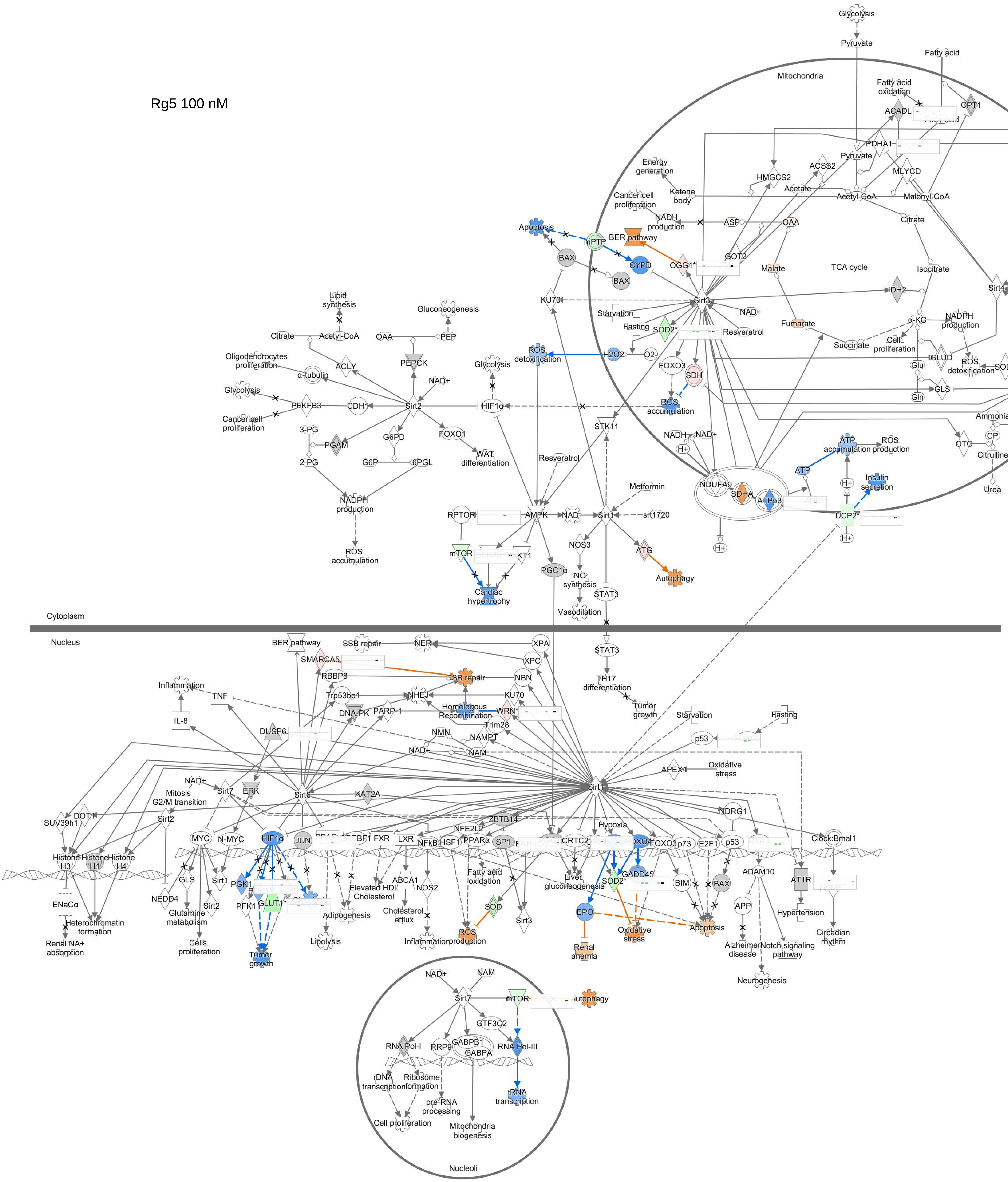

© 2000-2021 QIAGEN. All rights reserved.

Sirtuins are class III histone deacetylase enzymes that use NAD+ as a co-substrate for their enzymatic activities.
In mammals, there are 7 sirtuin members (SIRT1-7), which play important roles in aging, metabolism, cancer, inflammation, DNA repair and cellular responses to stress.

Rk1 100 nM

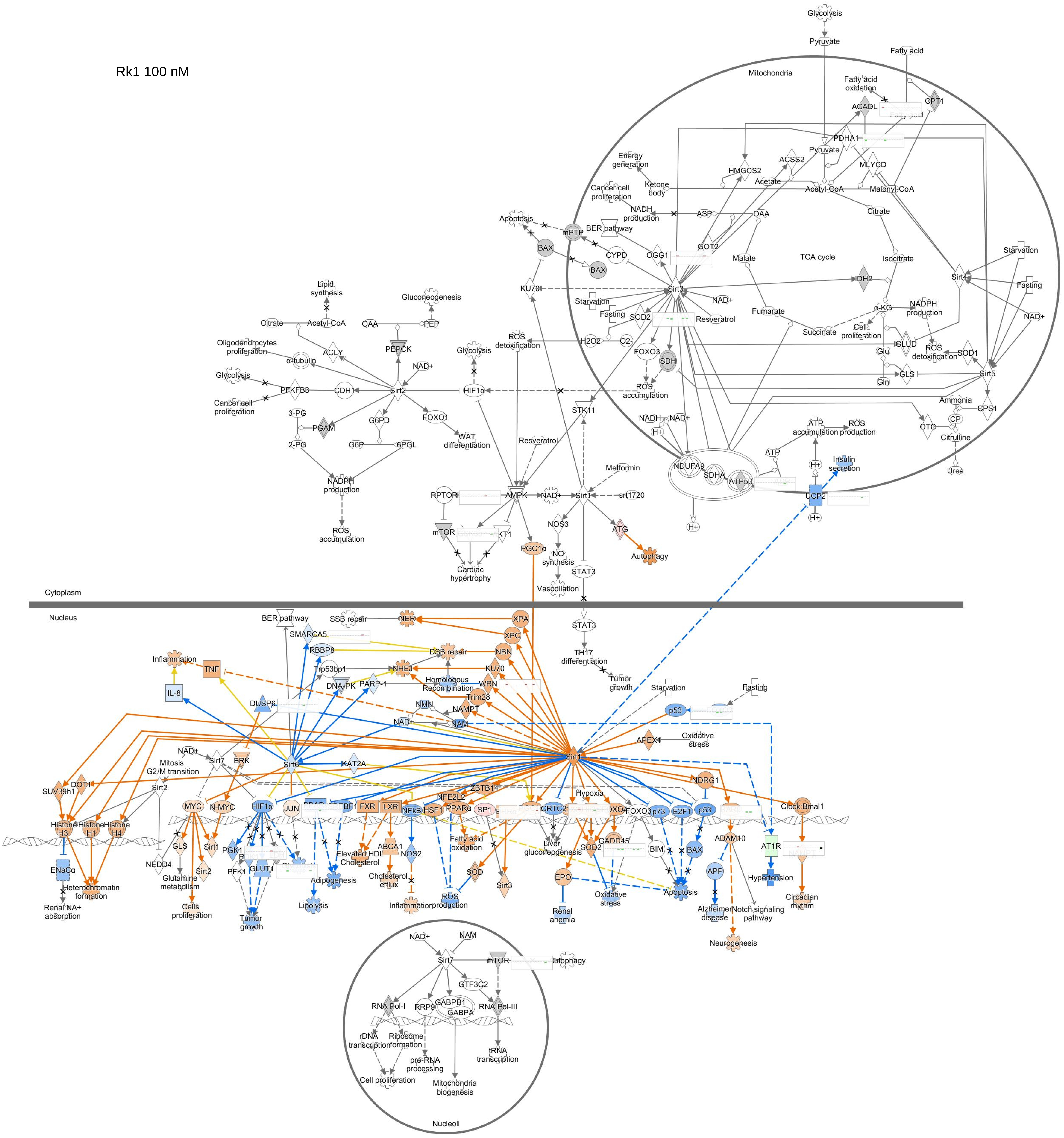

© 2000-2021 QIAGEN. All rights reserved.
