# Supplementary material for: Network Pharmacology of Ginseng (Part II): The Differential Effects of Red Ginseng and Ginsenoside Rg5 in Cancer and Heart Diseases as Determined by Transcriptomics
Source: Pharmaceuticals (Basel). 2021 Sep 30;14(10):1010. doi: 10.3390/ph14101010 (PMC8540751; doi:10.3390/ph14101010)
Supplement: Supplementary file 1 [file pharmaceuticals-14-01010-s001.zip › Supplement 9 Adrenomodulin signaling .pdf]

Adrenomedullin (AM) is a member of the calcitonin gene-related peptide (CGRP) family. It is produced by many different cell types throughout the body, with the possible exception of the thyroid and thymus, and has vasodilatory, hypotensive and angiogenic activity. AM also stimulates cell growth and inhibits apoptosis in many tumor cells.

WG 10000 ng/ml

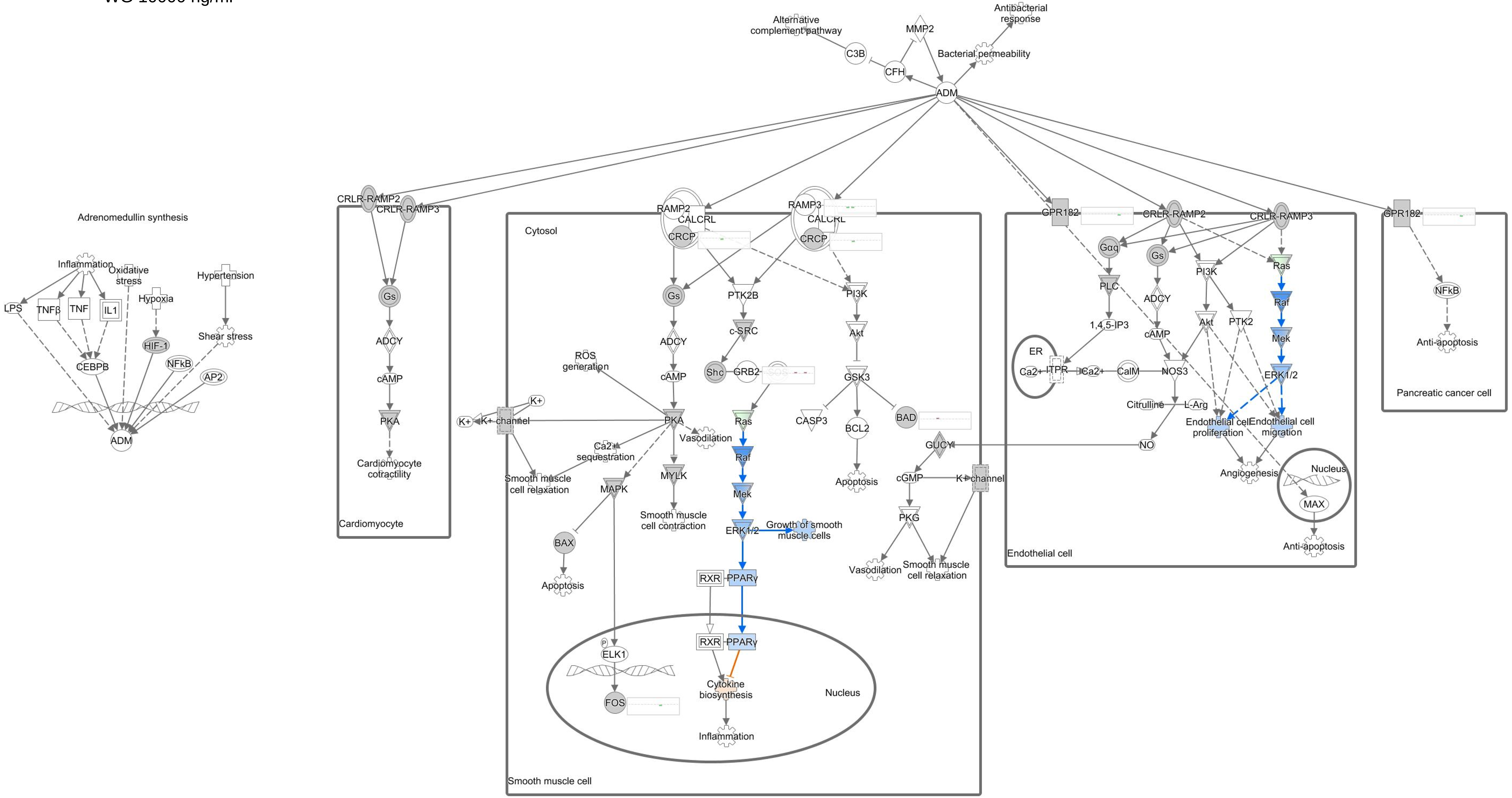

© 2000-2021 QIAGEN. All rights reserved.

Adrenomedullin (AM) is a member of the calcitonin gene-related peptide (CGRP) family. It is produced by many different cell types throughout the body, with the possible exception of the thyroid and thymus, and has vasodilatory, hypotensive and angiogenic activity. AM also stimulates cell growth and inhibits apoptosis in many tumor cells.

HRG80 10000 ng/ml

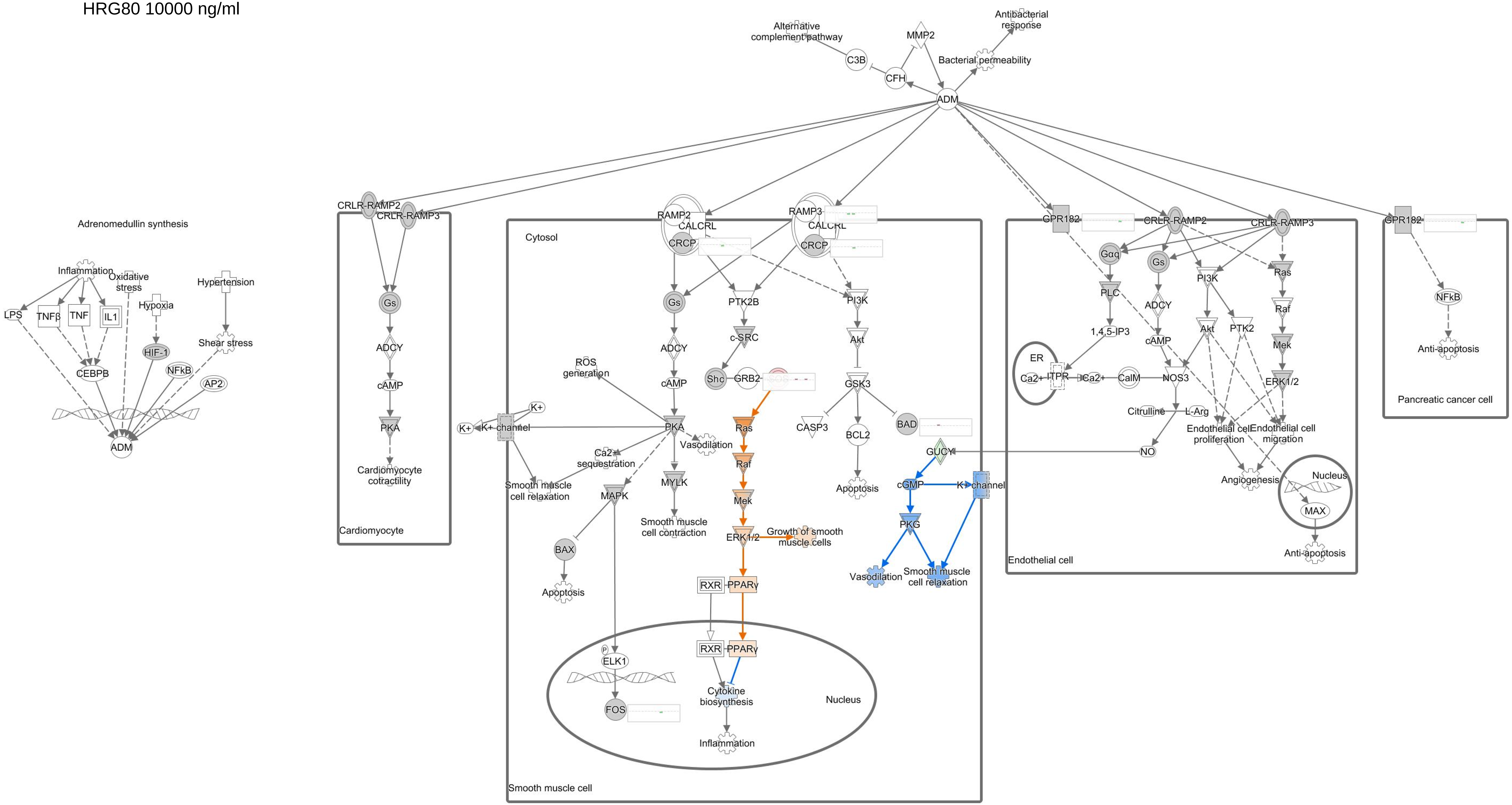

© 2000-2021 QIAGEN. All rights reserved.

Adrenomedullin (AM) is a member of the calcitonin gene-related peptide (CGRP) family. It is produced by many different cell types throughout the body, with the possible exception of the thyroid and thymus, and has vasodilatory, hypotensive and angiogenic activity. AM also stimulates cell growth and inhibits apoptosis in many tumor cells.

HRG80 1000 ng/ml

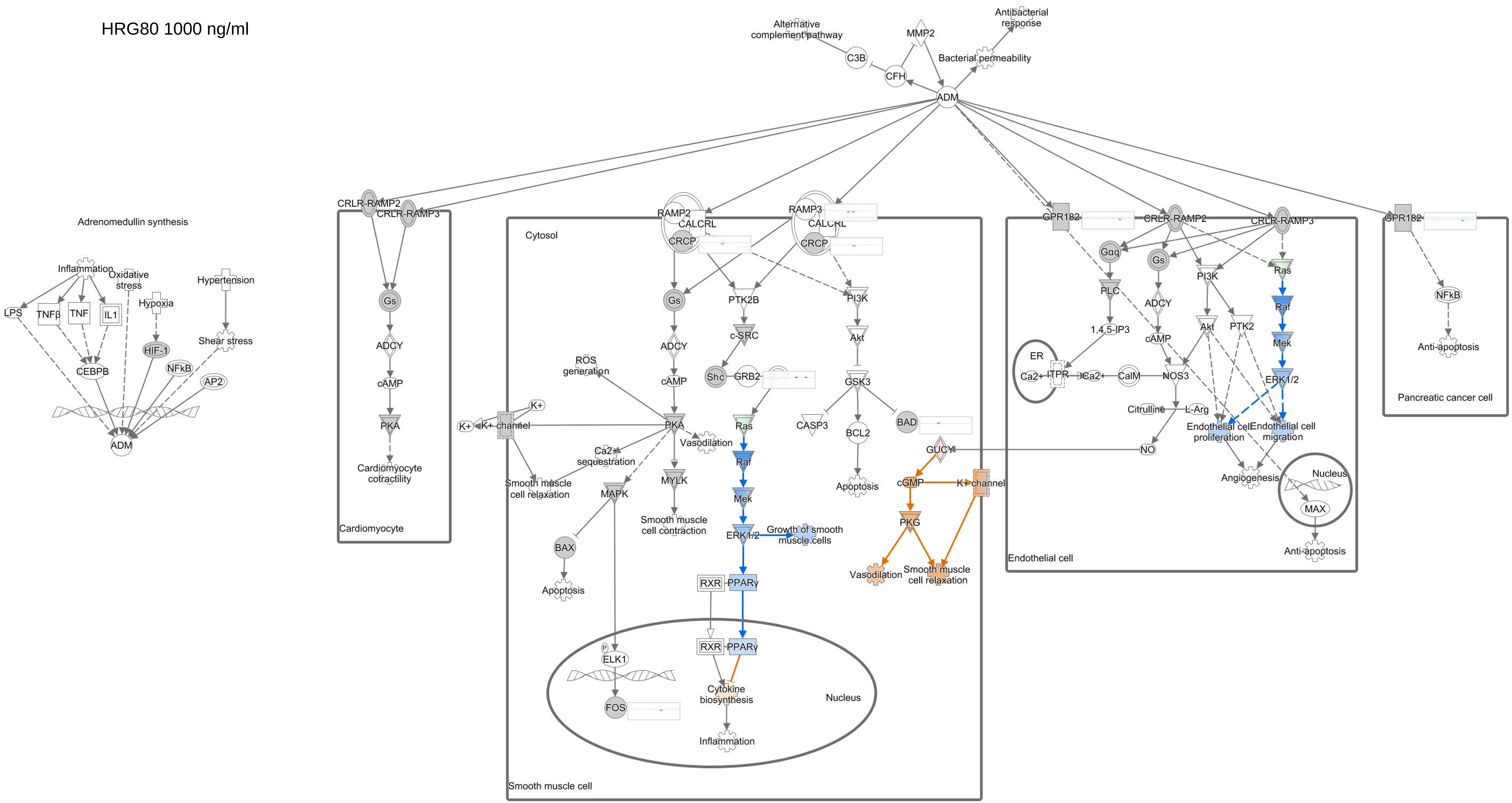

© 2000-2021 QIAGEN. All rights reserved.

Adrenomedullin (AM) is a member of the calcitonin gene-related peptide (CGRP) family. It is produced by many different cell types throughout the body, with the possible exception of the thyroid and thymus, and has vasodilatory, hypotensive and angiogenic activity. AM also stimulates cell growth and inhibits apoptosis in many tumor cells.

HRG80 100 ng/ml

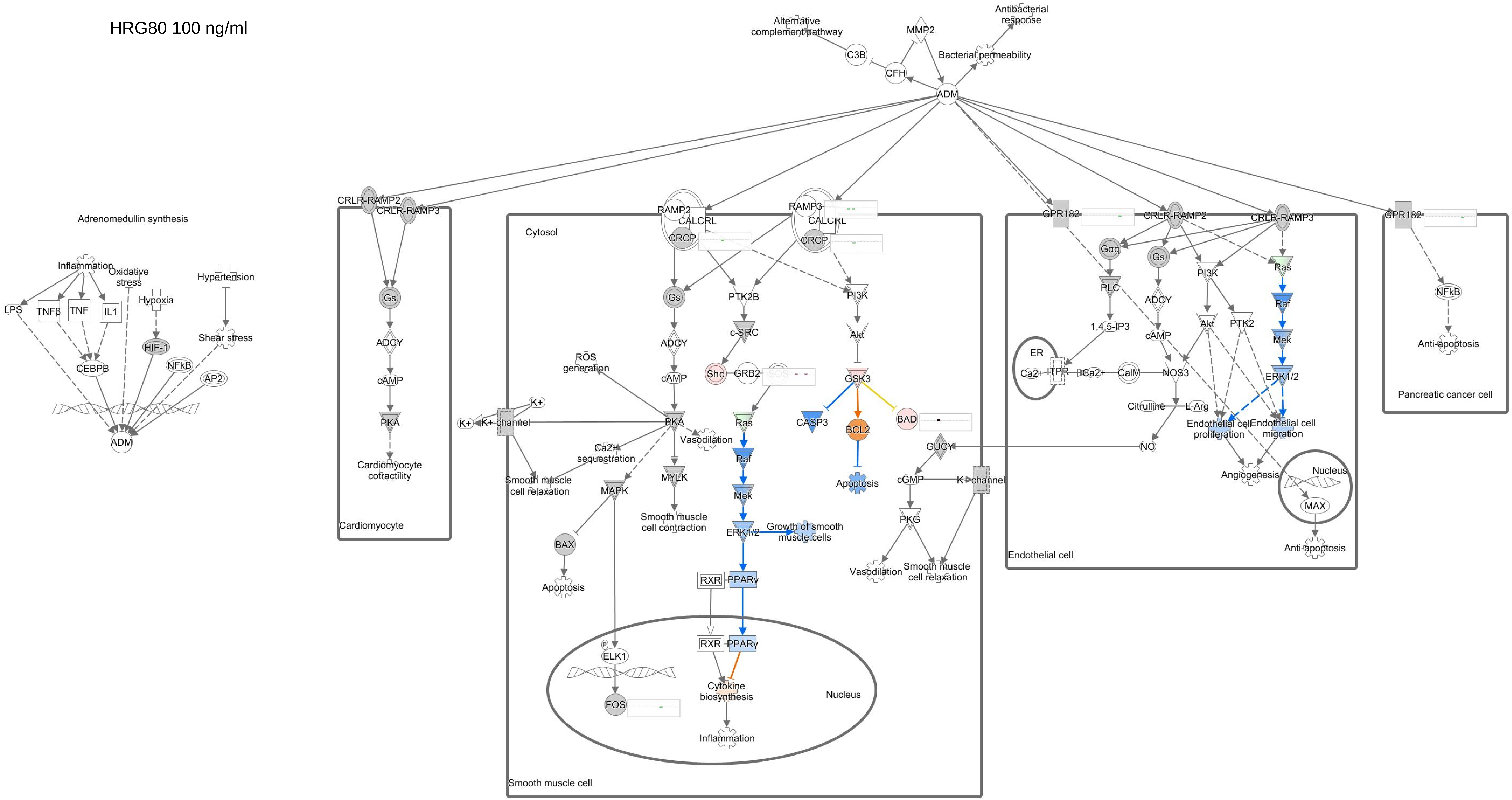

© 2000-2021 QIAGEN. All rights reserved.

Adrenomedullin (AM) is a member of the calcitonin gene-related peptide (CGRP) family. It is produced by many different cell types throughout the body, with the possible exception of the thyroid and thymus, and has vasodilatory, hypotensive and angiogenic activity. AM also stimulates cell growth and inhibits apoptosis in many tumor cells.

HRG80 10 ng/ml

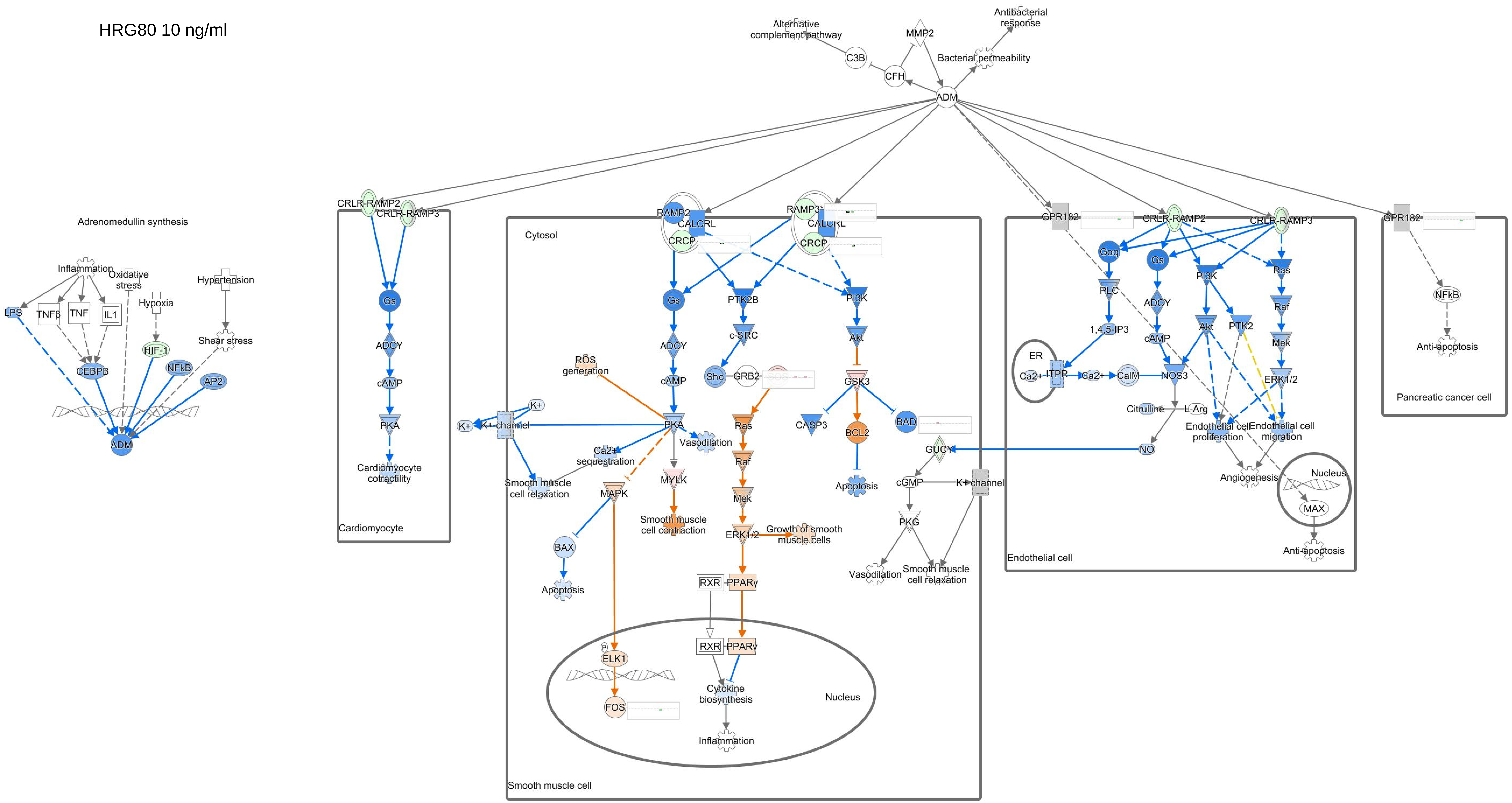

© 2000-2021 QIAGEN. All rights reserved.

Adrenomedullin (AM) is a member of the calcitonin gene-related peptide (CGRP) family. It is produced by many different cell types throughout the body, with the possible exception of the thyroid and thymus, and has vasodilatory, hypotensive and angiogenic activity. AM also stimulates cell growth and inhibits apoptosis in many tumor cells.

HRG80 0.01 ng/ml

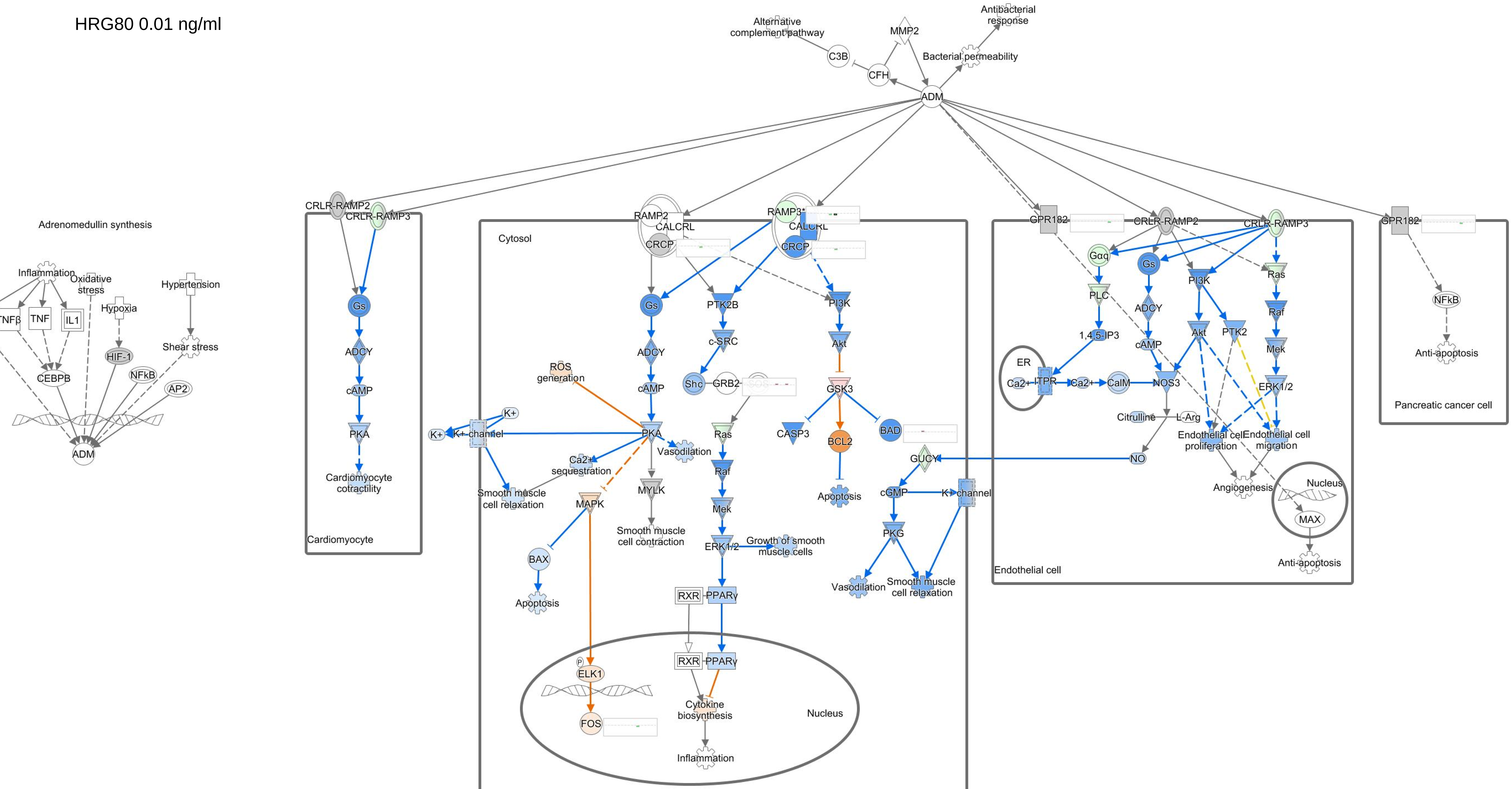

© 2000-2021 QIAGEN. All rights reserved.

Adrenomedullin (AM) is a member of the calcitonin gene-related peptide (CGRP) family. It is produced by many different cell types throughout the body, with the possible exception of the thyroid and thymus, and has vasodilatory, hypotensive and angiogenic activity. AM also stimulates cell growth and inhibits apoptosis in many tumor cells.

Rb1 100 nM

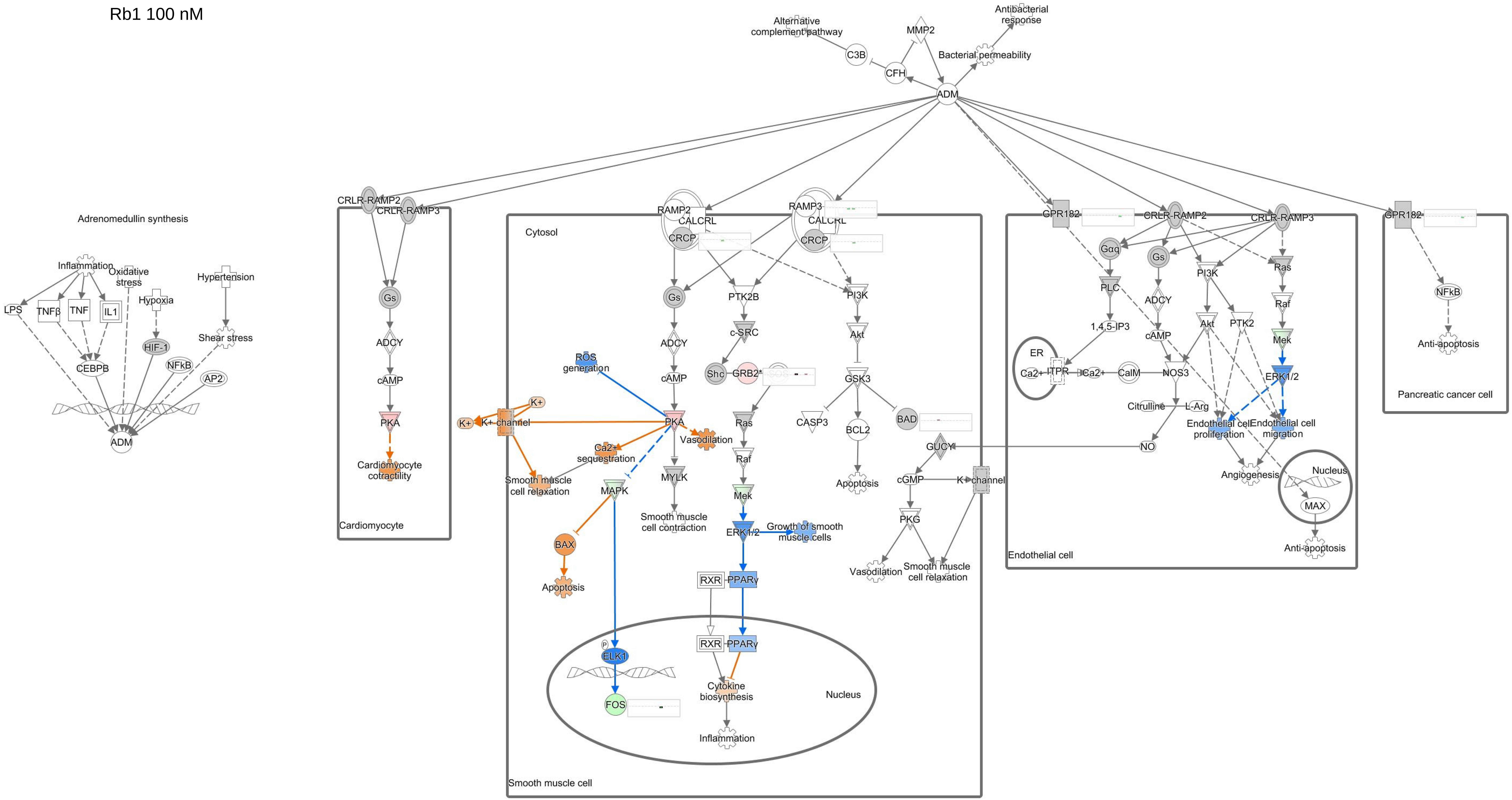

© 2000-2021 QIAGEN. All rights reserved.

Adrenomedullin (AM) is a member of the calcitonin gene-related peptide (CGRP) family. It is produced by many different cell types throughout the body, with the possible exception of the thyroid and thymus, and has vasodilatory, hypotensive and angiogenic activity. AM also stimulates cell growth and inhibits apoptosis in many tumor cells.

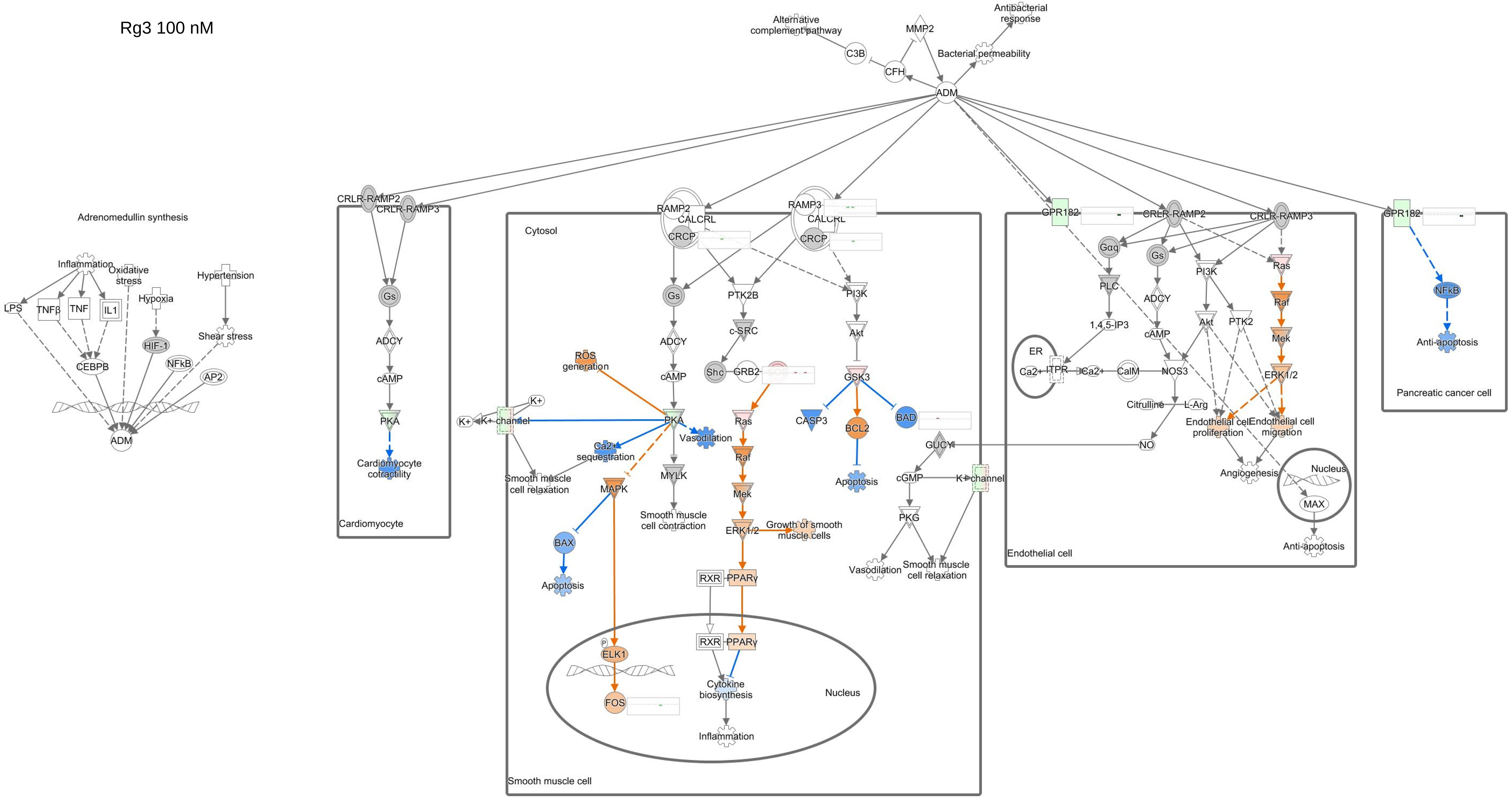

© 2000-2021 QIAGEN. All rights reserved.

Adrenomedullin (AM) is a member of the calcitonin gene-related peptide (CGRP) family. It is produced by many different cell types throughout the body, with the possible exception of the thyroid and thymus, and has vasodilatory, hypotensive and angiogenic activity. AM also stimulates cell growth and inhibits apoptosis in many tumor cells.

Rg5 100 nM

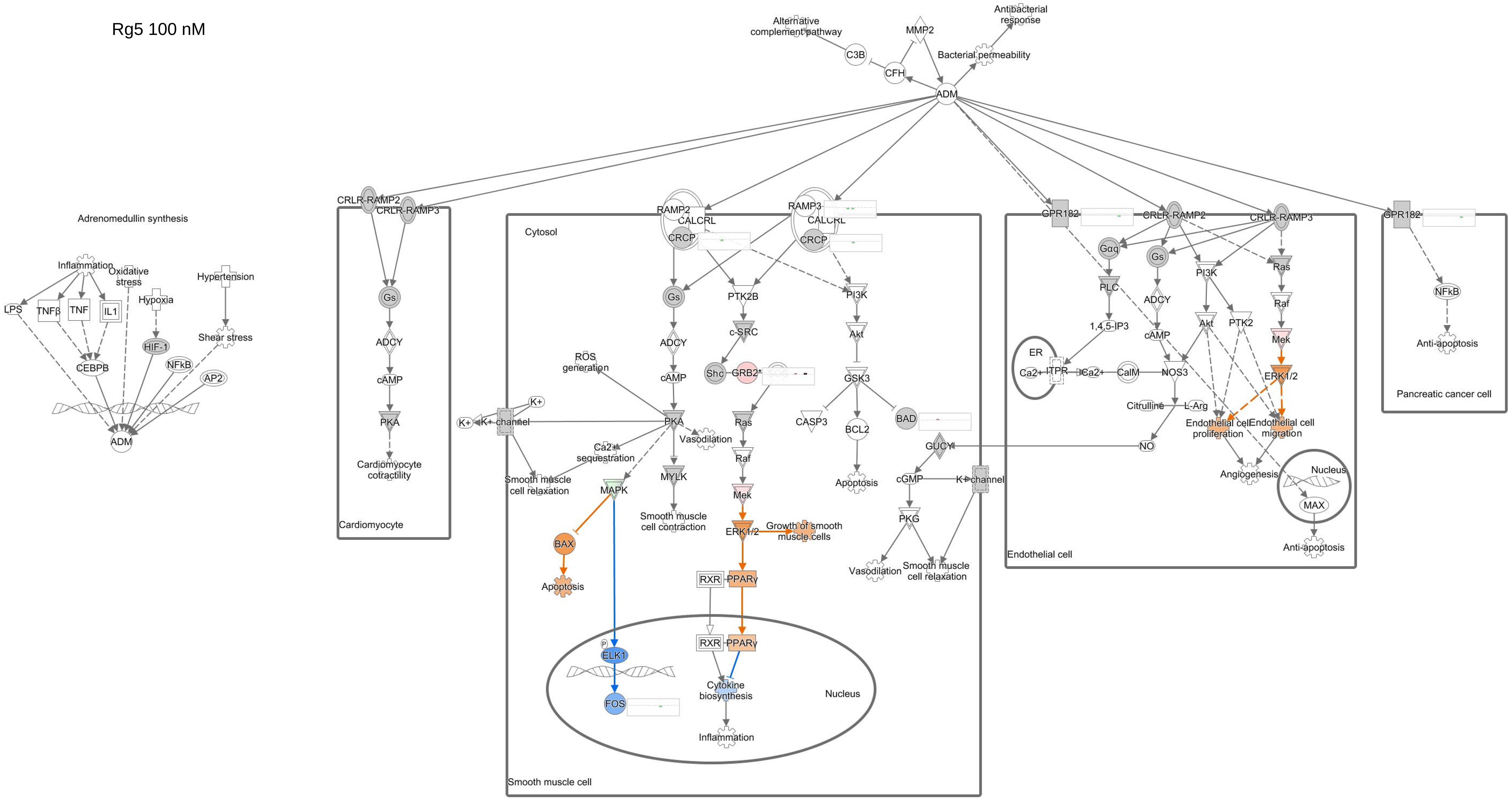

© 2000-2021 QIAGEN. All rights reserved.

Adrenomedullin (AM) is a member of the calcitonin gene-related peptide (CGRP) family. It is produced by many different cell types throughout the body, with the possible exception of the thyroid and thymus, and has vasodilatory, hypotensive and angiogenic activity. AM also stimulates cell growth and inhibits apoptosis in many tumor cells.

Rk1 100 nM

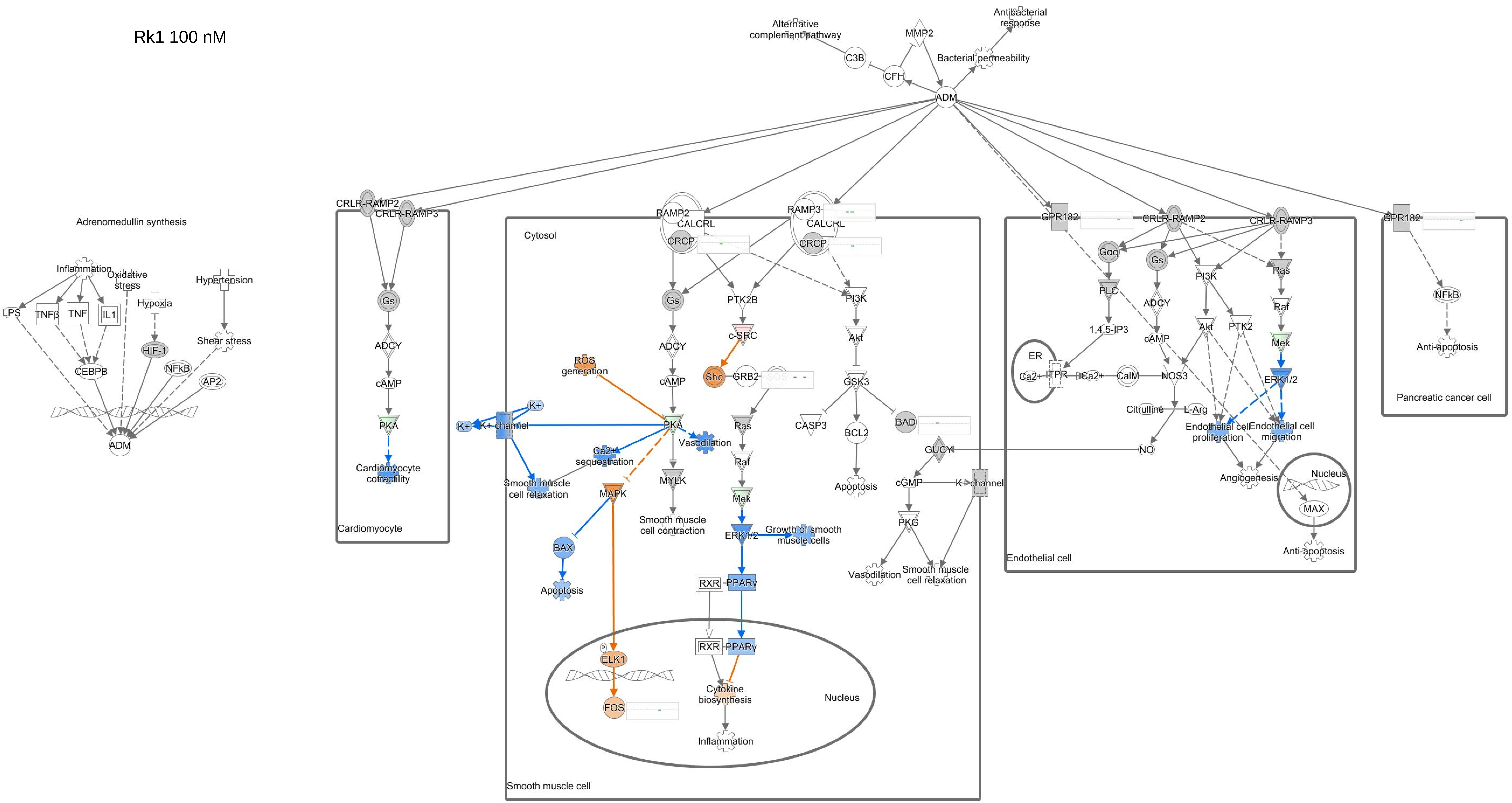

© 2000-2021 QIAGEN. All rights reserved.
