# Supplementary figures and images for: Network Pharmacology of Ginseng (Part II): The Differential Effects of Red Ginseng and Ginsenoside Rg5 in Cancer and Heart Diseases as Determined by Transcriptomics
Source: Pharmaceuticals (Basel). 2021 Sep 30;14(10):1010. doi: 10.3390/ph14101010 (PMC8540751; doi:10.3390/ph14101010)

WG 10000 ng/ml

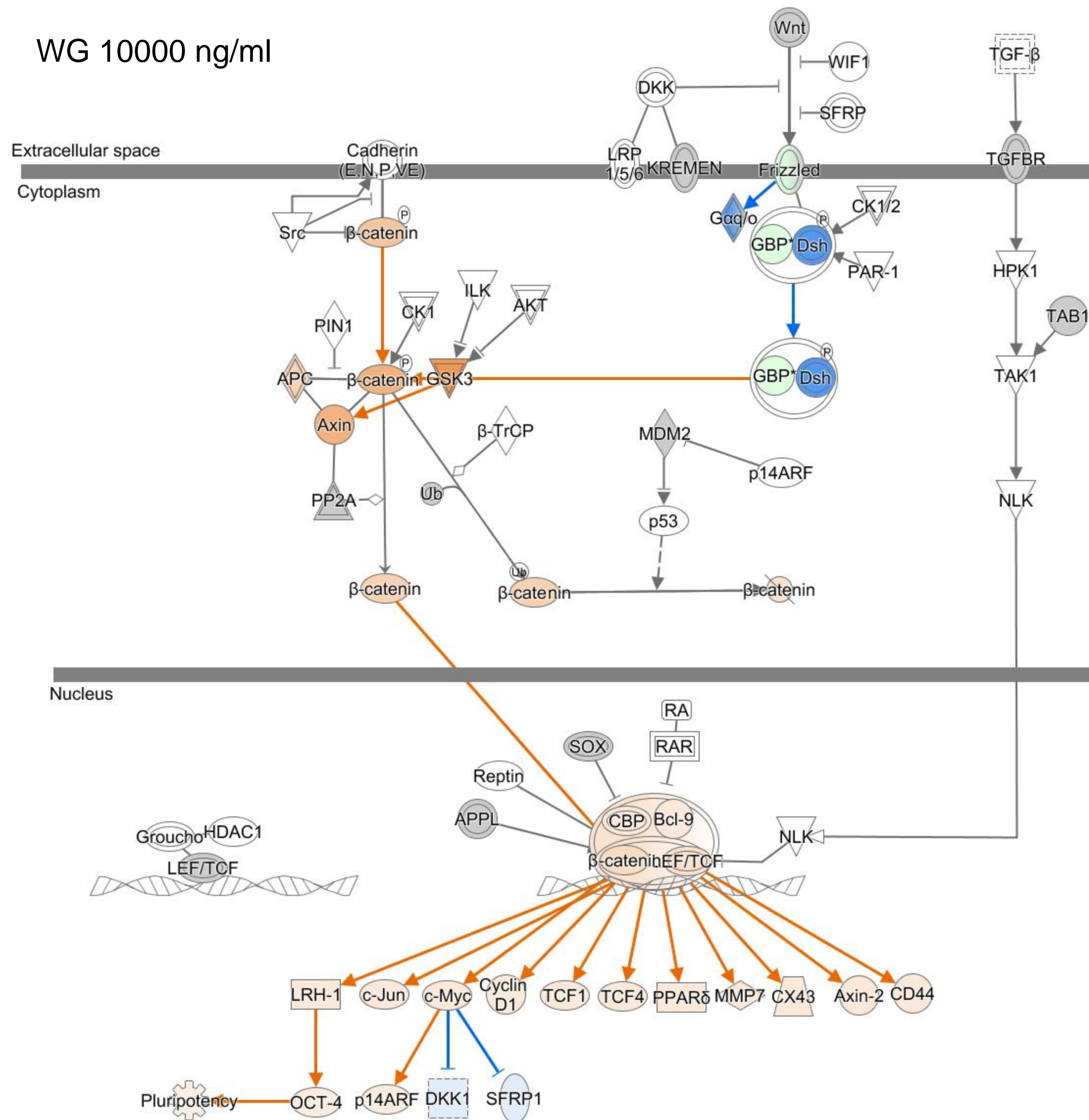

HRG80 10000 ng/ml

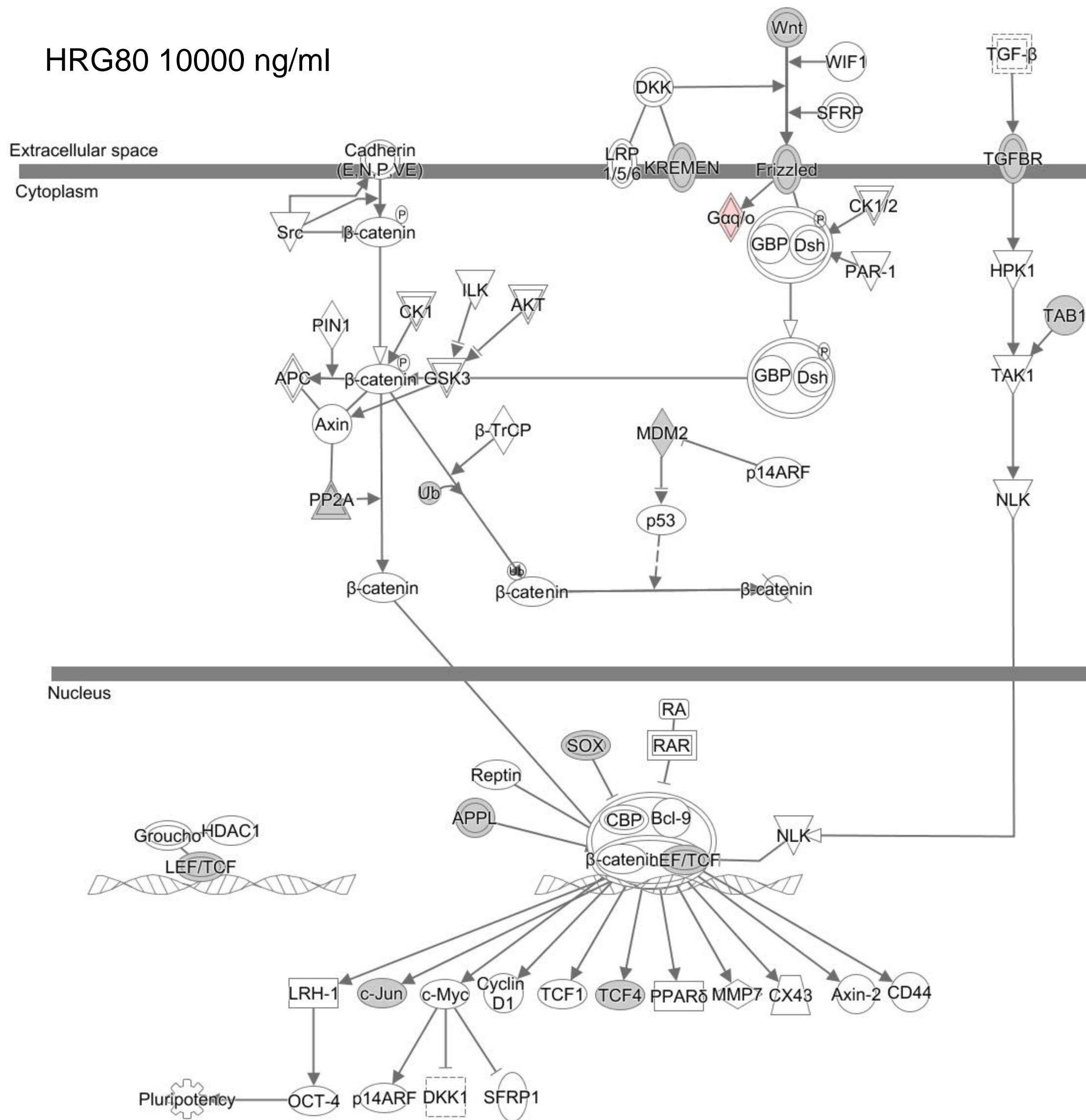

HRG80 1000 ng/ml

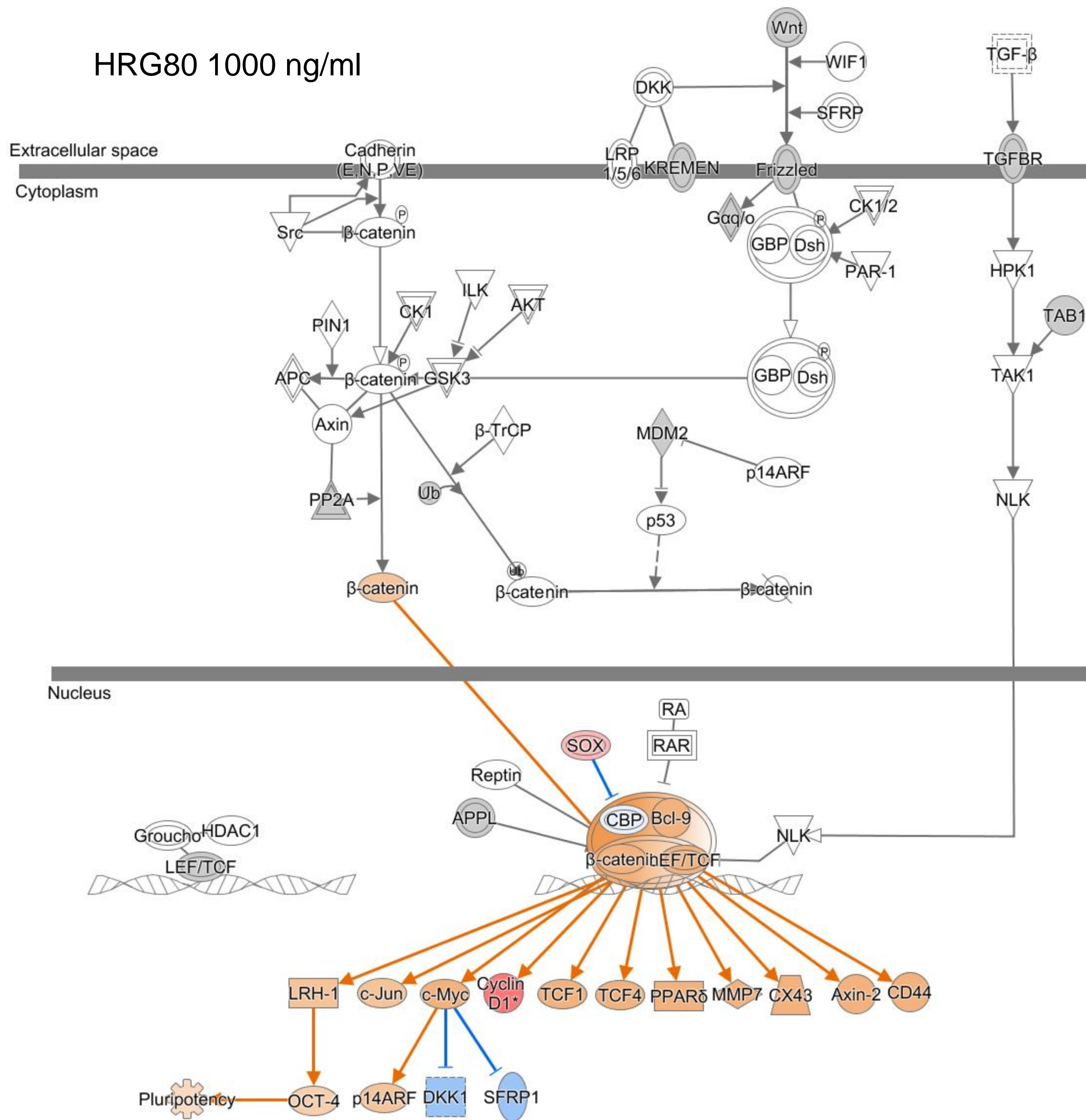

HRG80 100 ng/ml

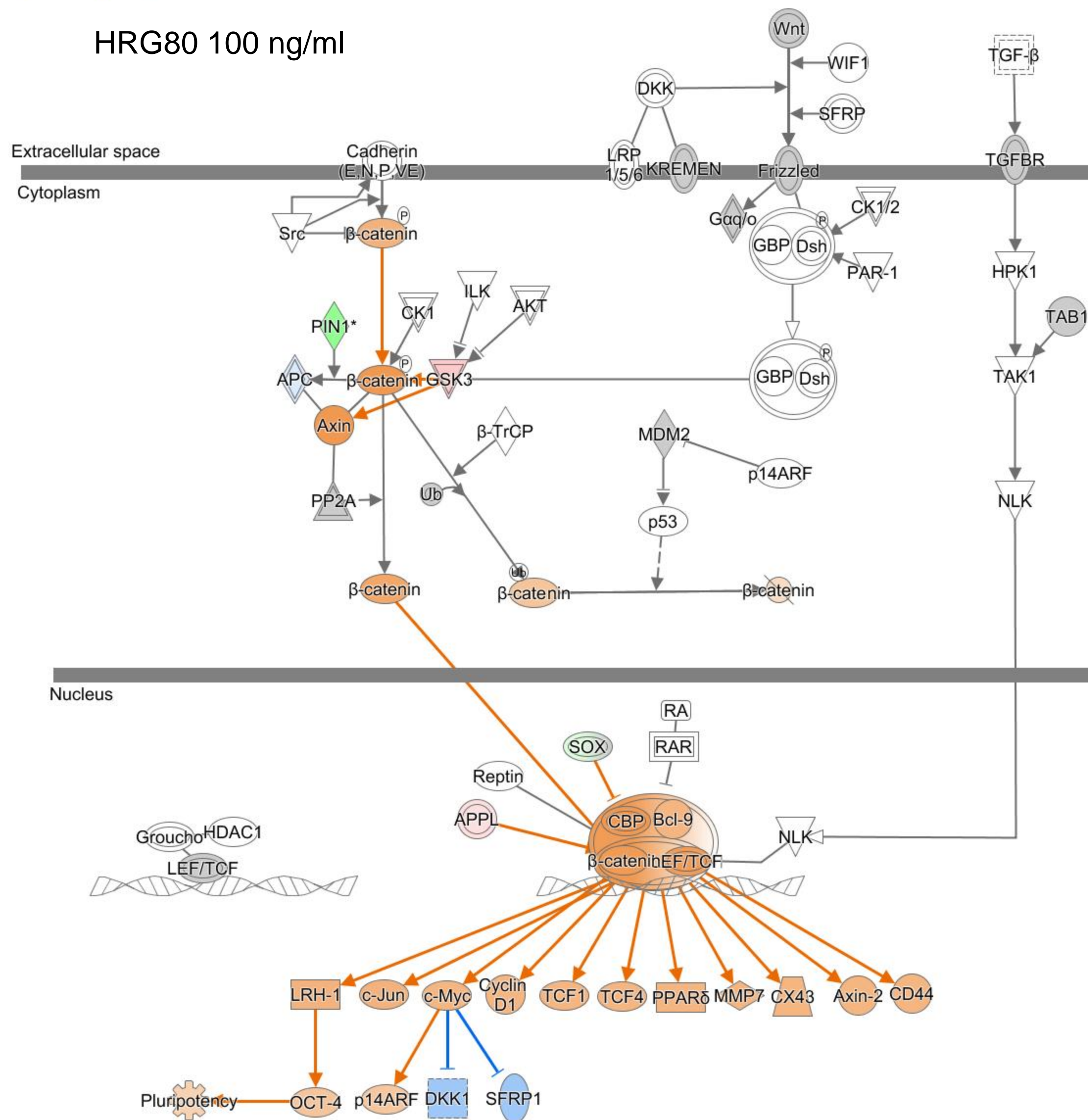

HRG80 10 ng/ml

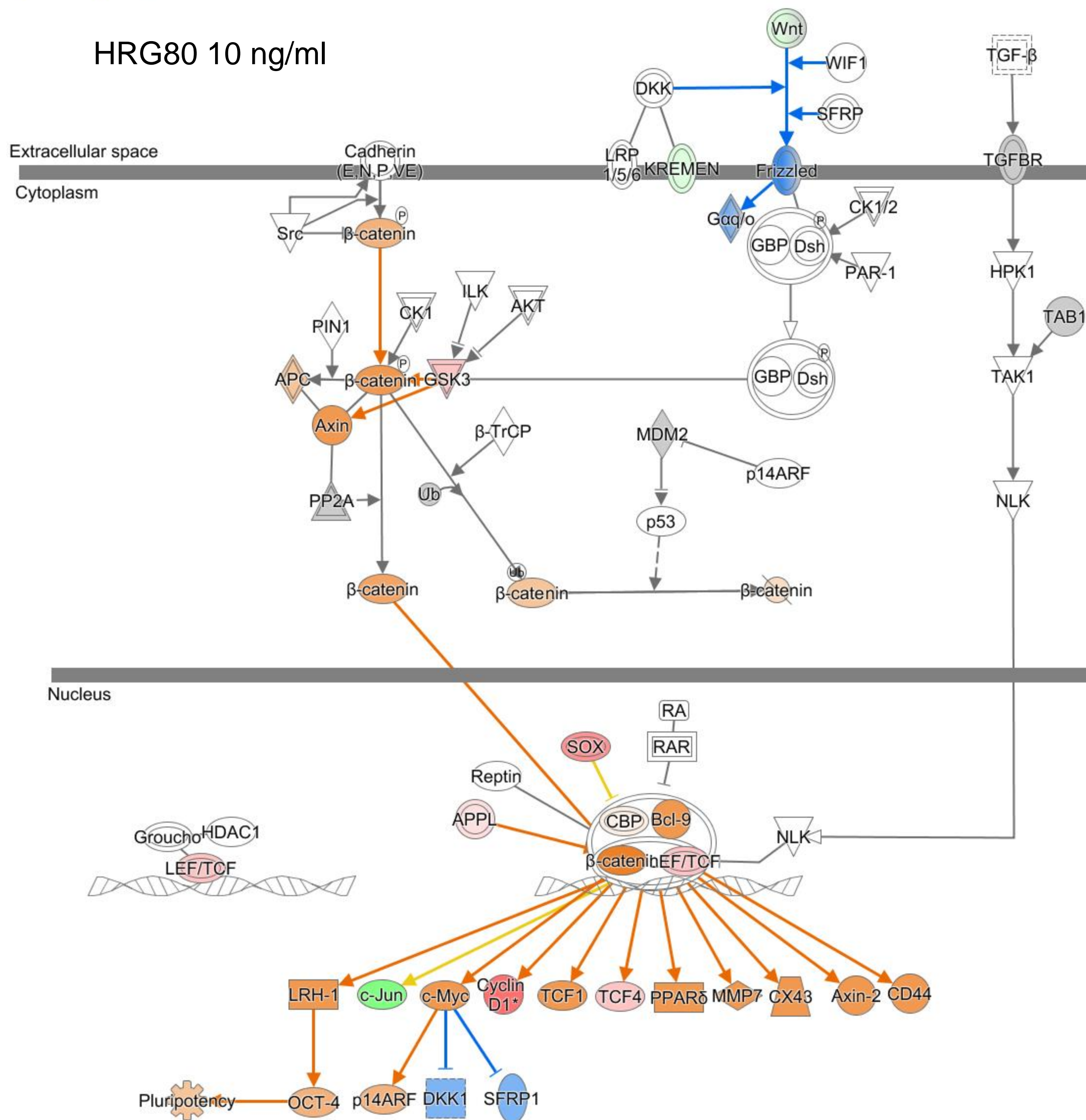

HRG80 0.01 ng/ml

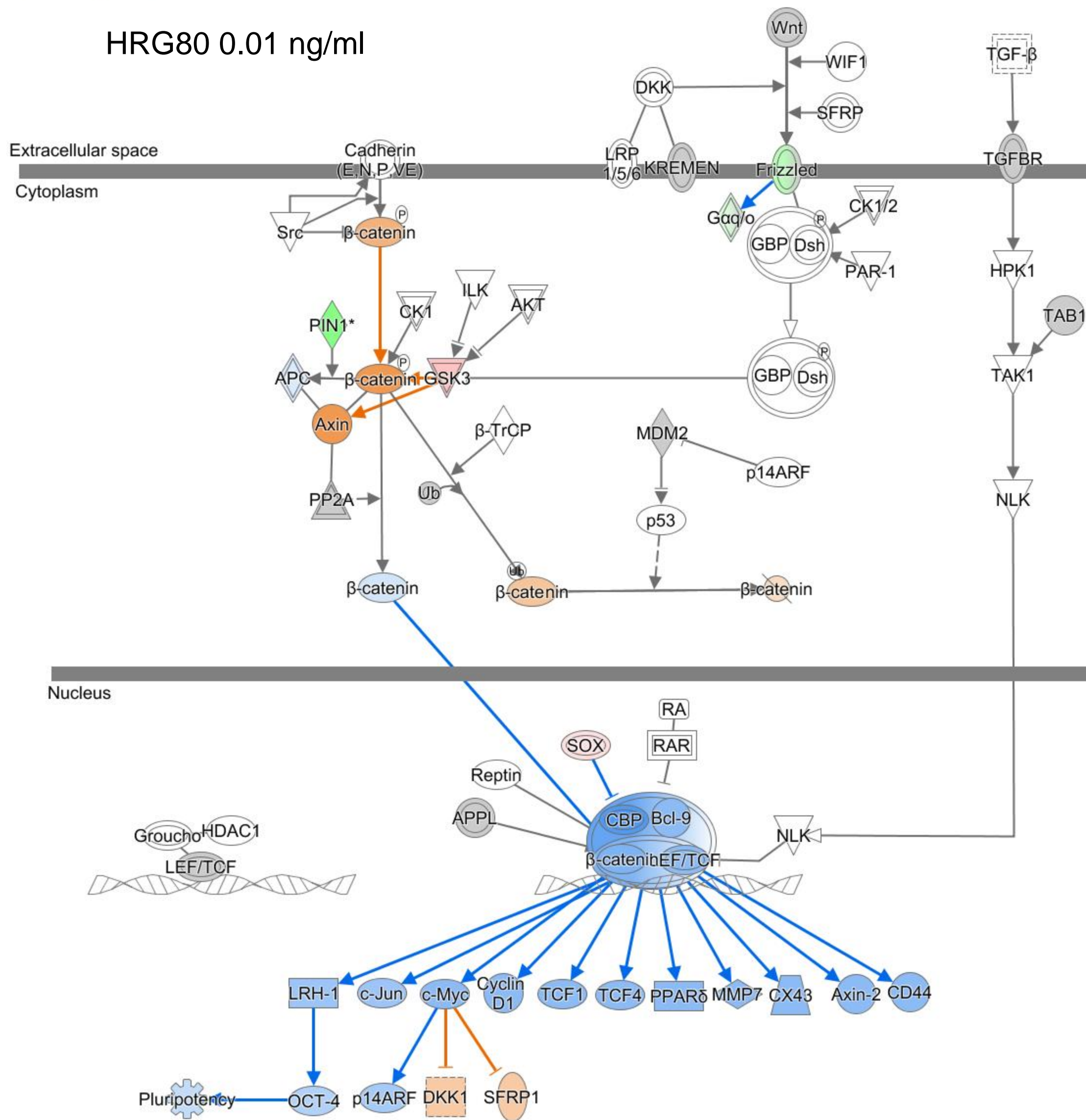

Rb1 100 nM

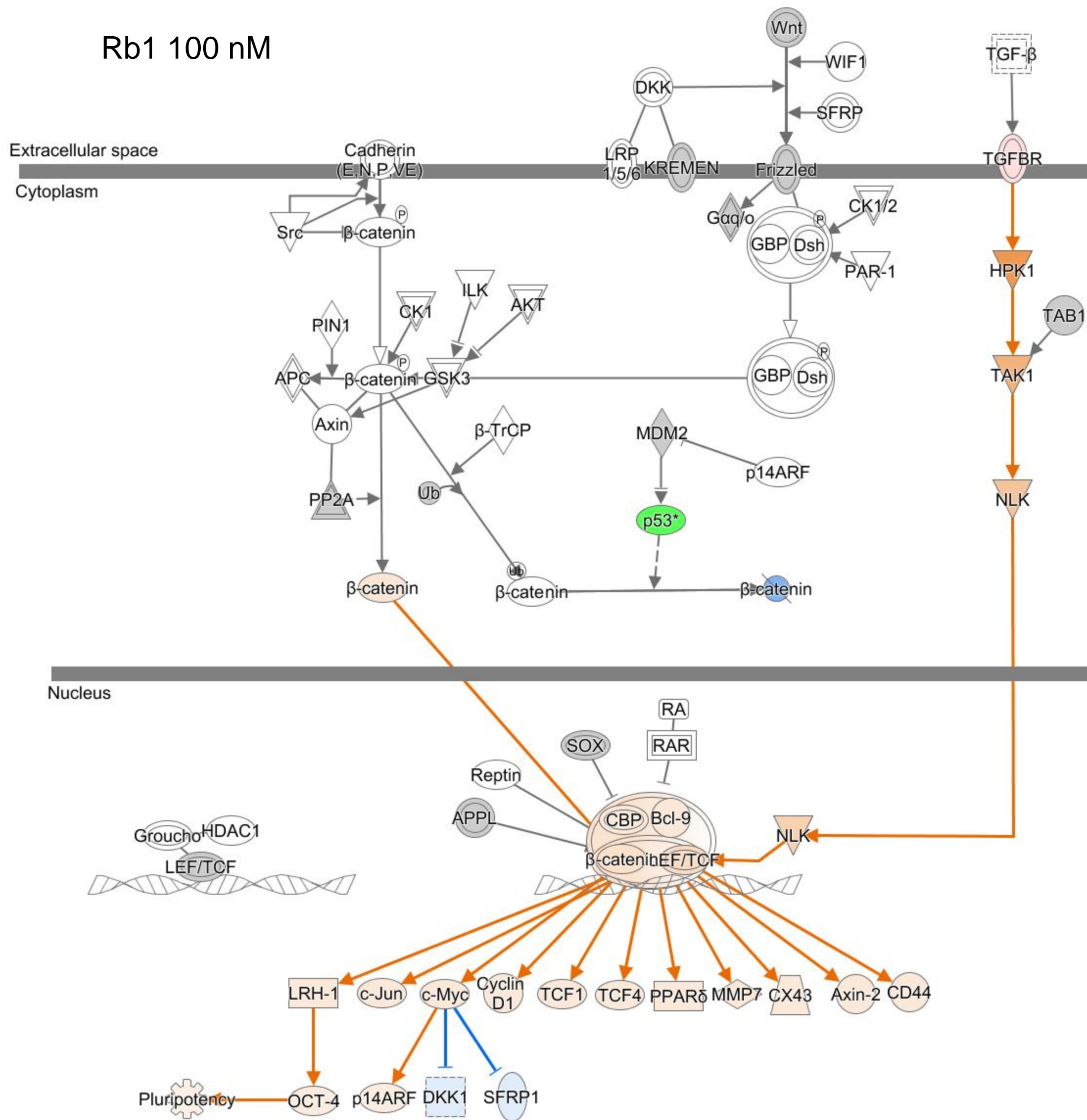

Rg3 100 nM

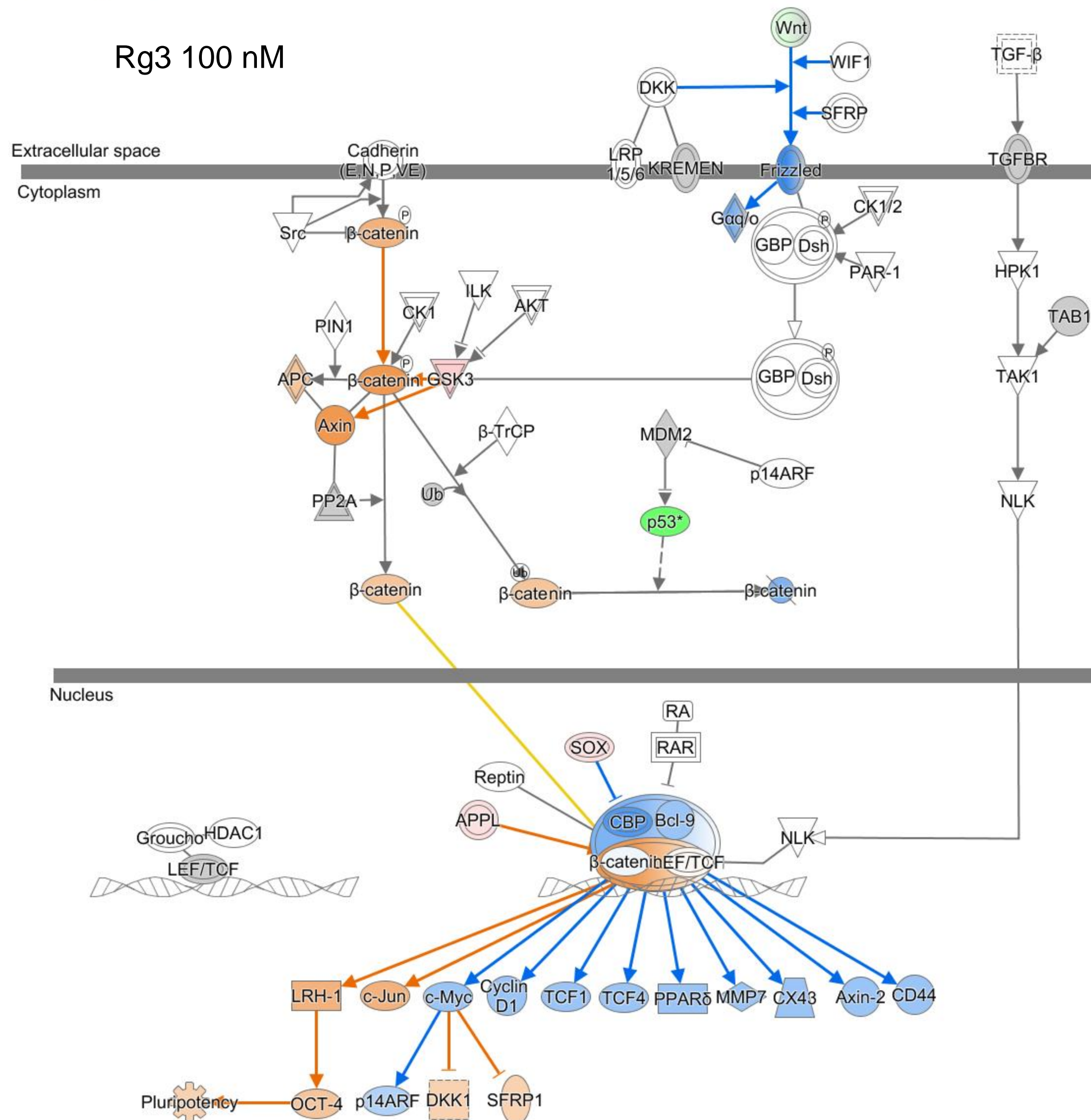

Rg5 100 nM

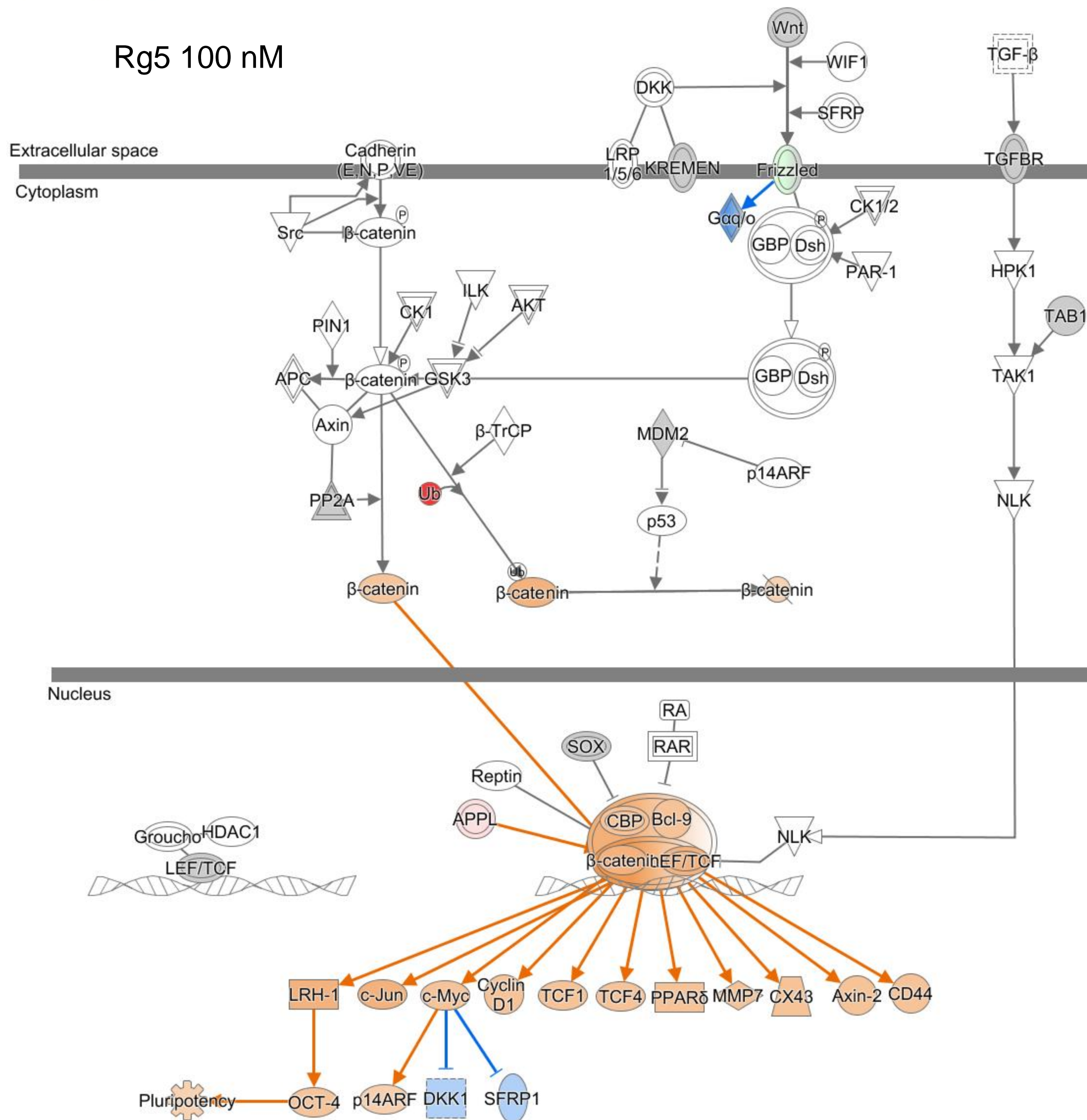

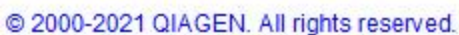

Supplement: Supplementary file 1 [file pharmaceuticals-14-01010-s001.zip › Supplement 10 WNT - carenin siignalling.pdf]

WG 10000 ng/ml

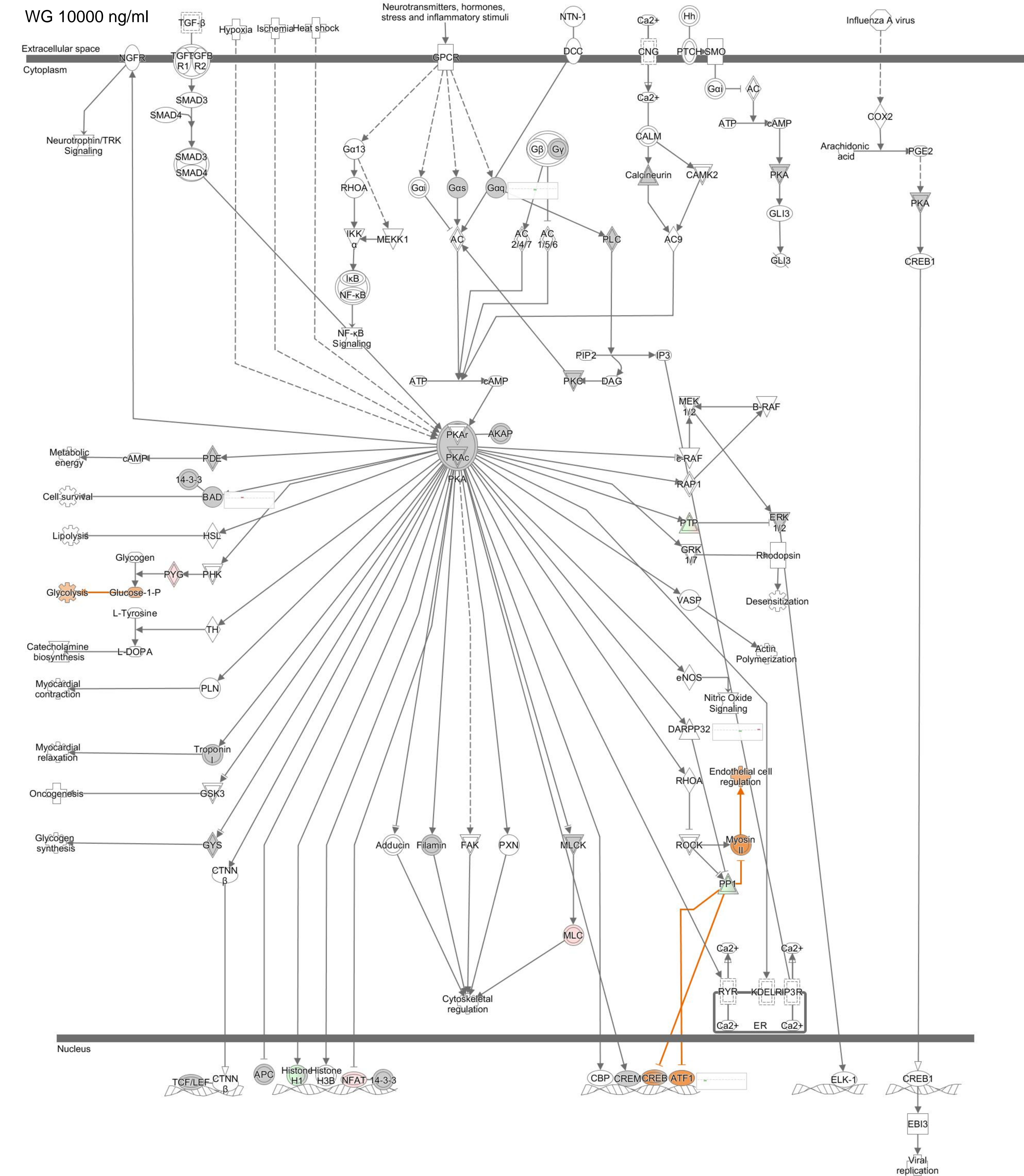

HRG80 10000 ng/ml

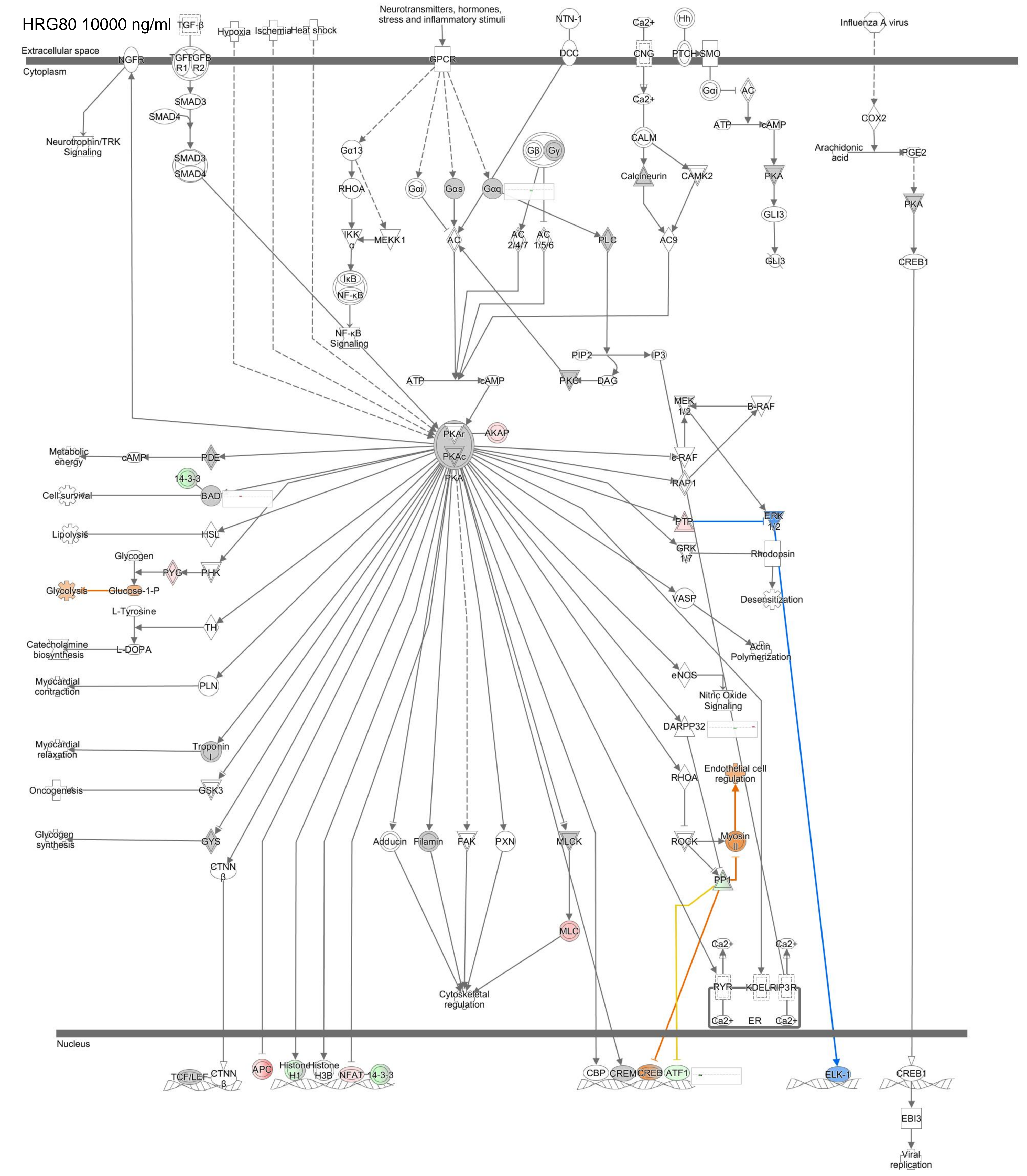

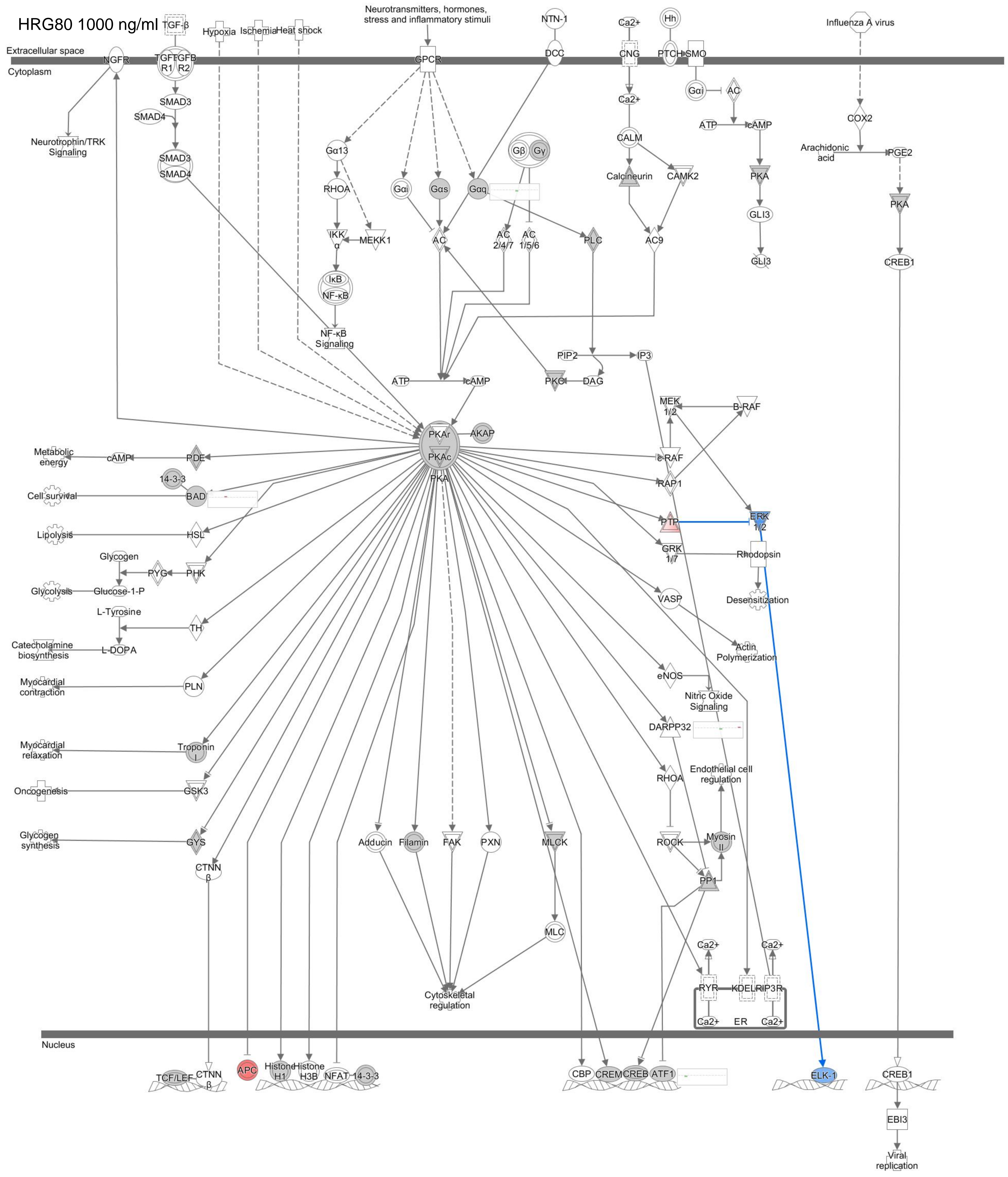

HRG80 100 ng/ml

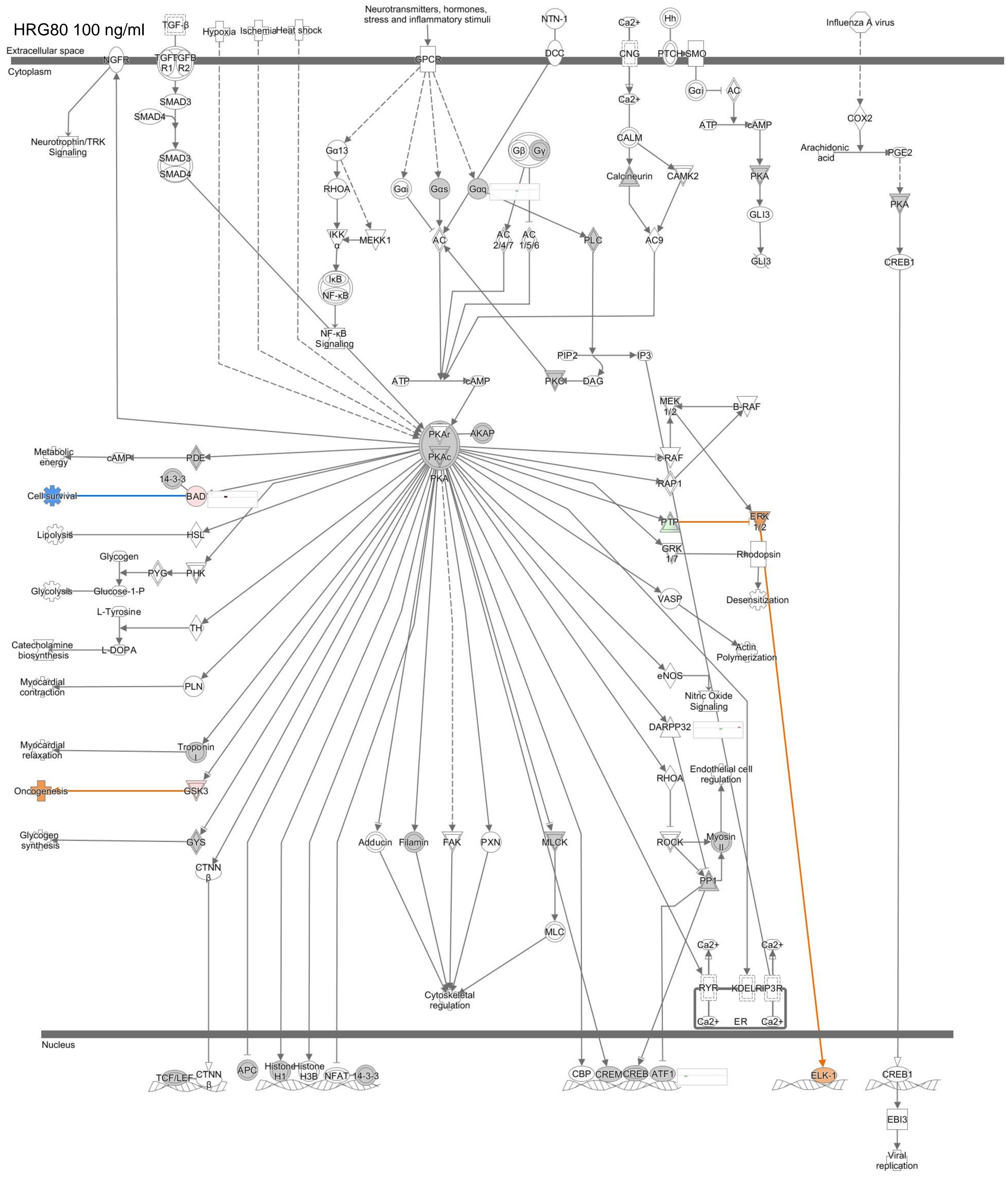



HRG80 0.01 ng/ml

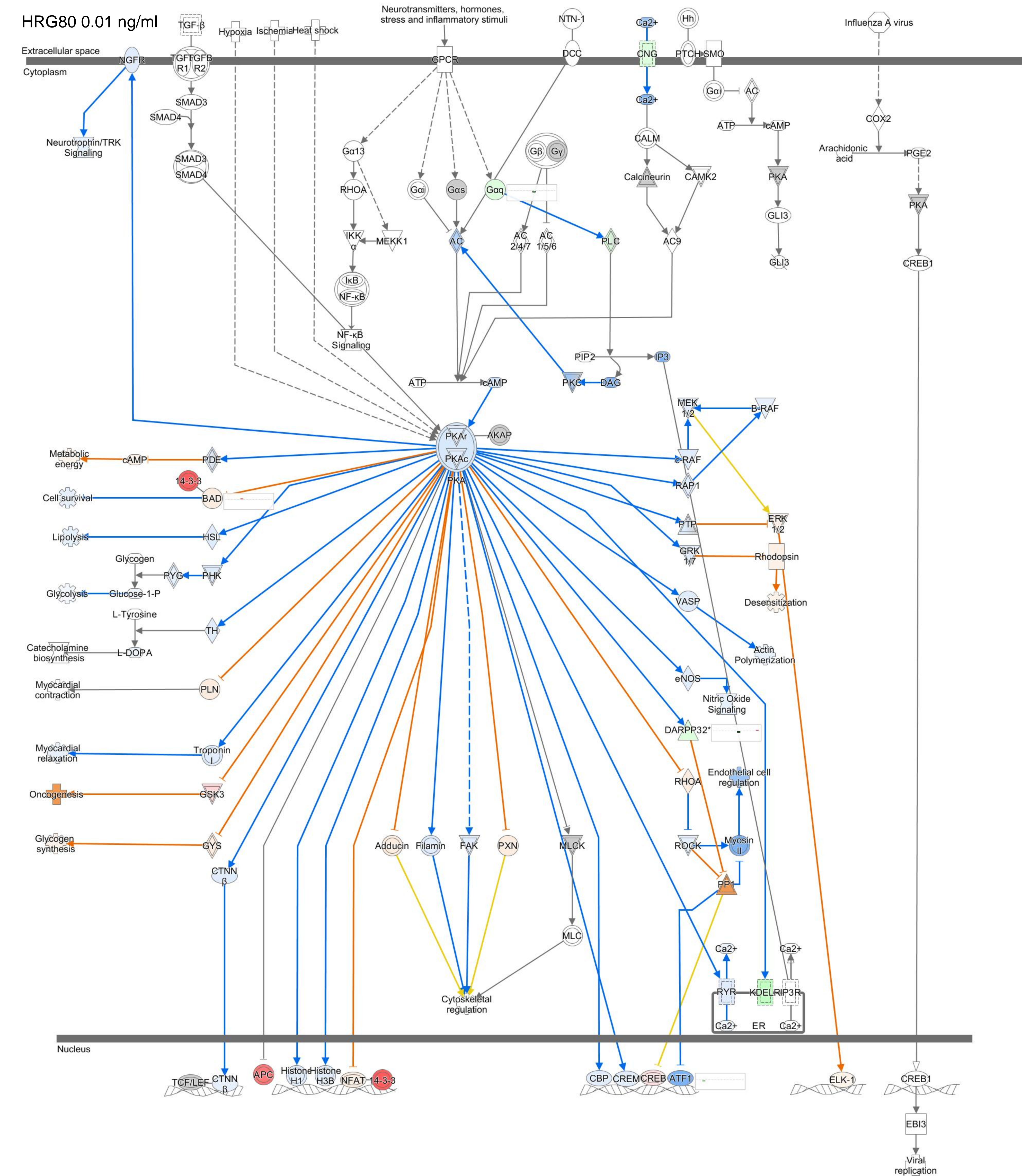

Rb1 100 nM

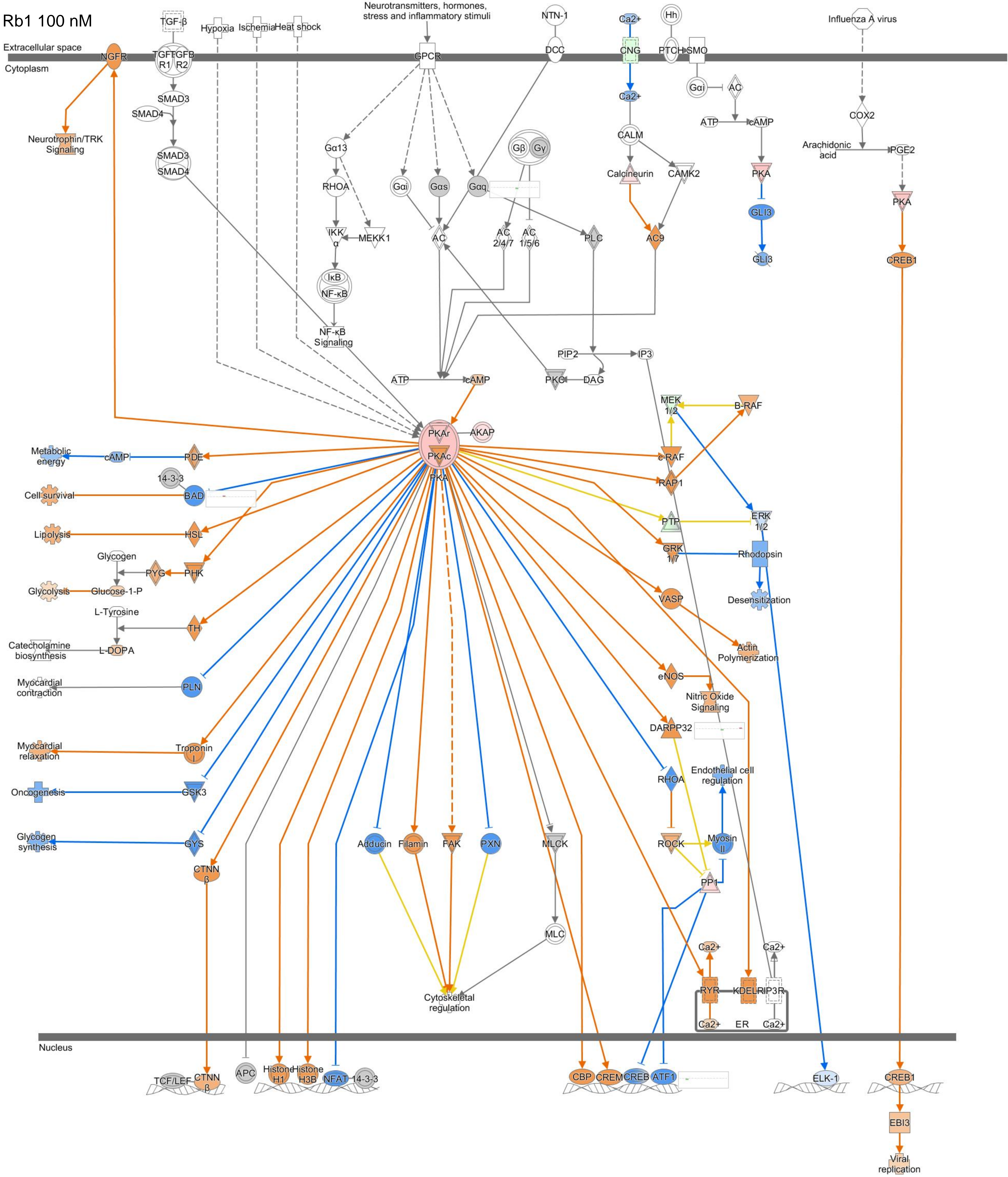

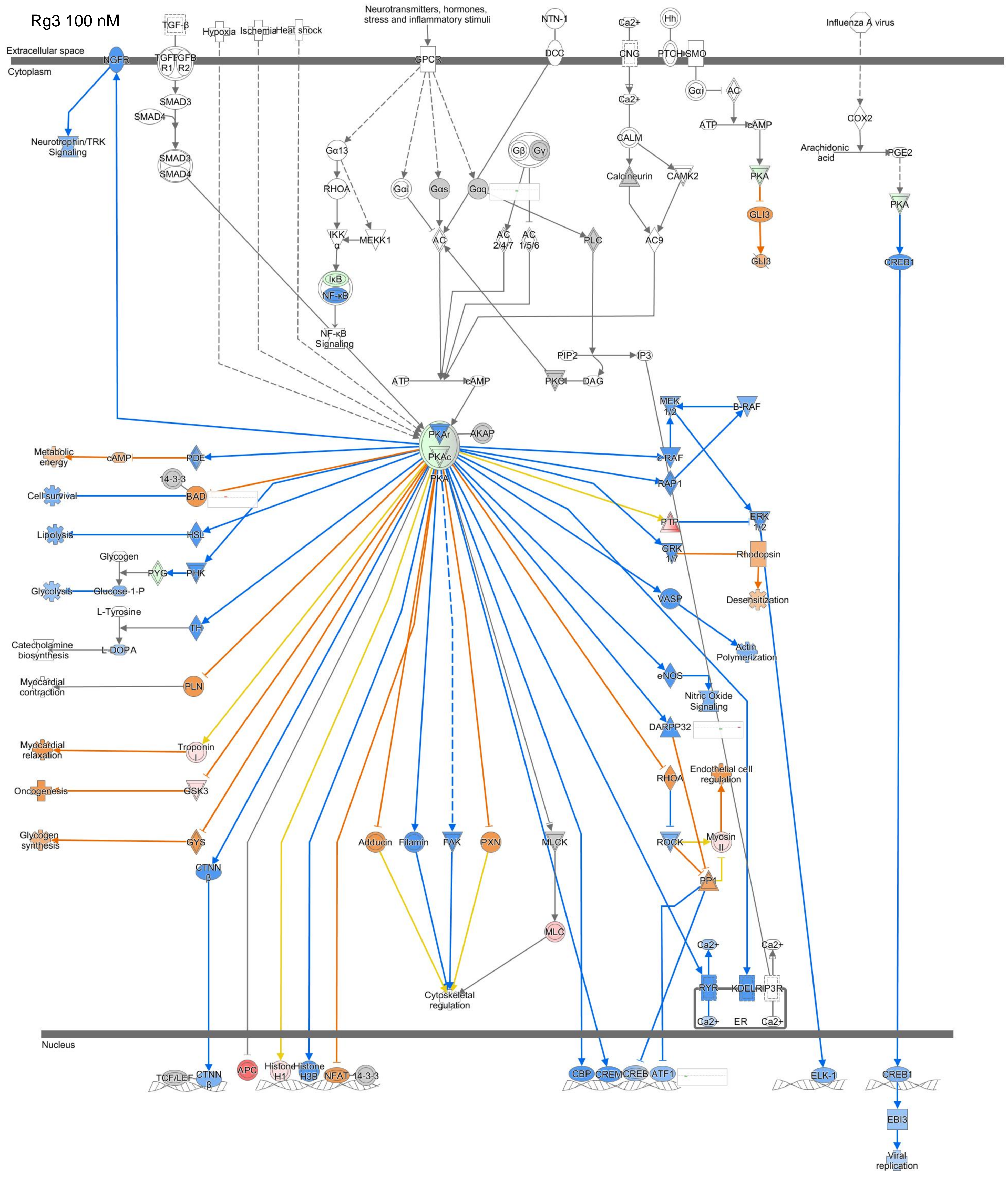

Rg5 100 nM

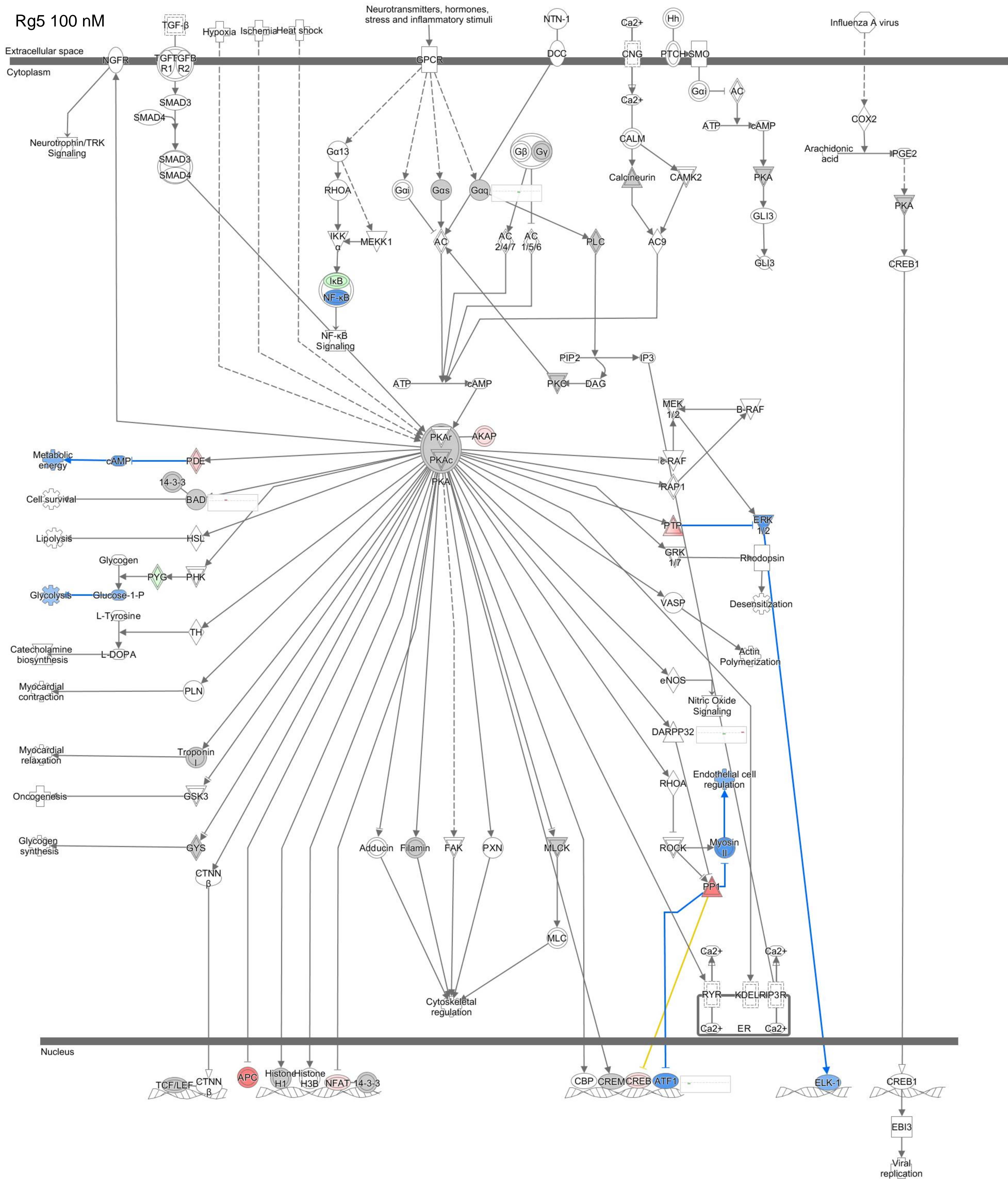

Rk1 100 nM

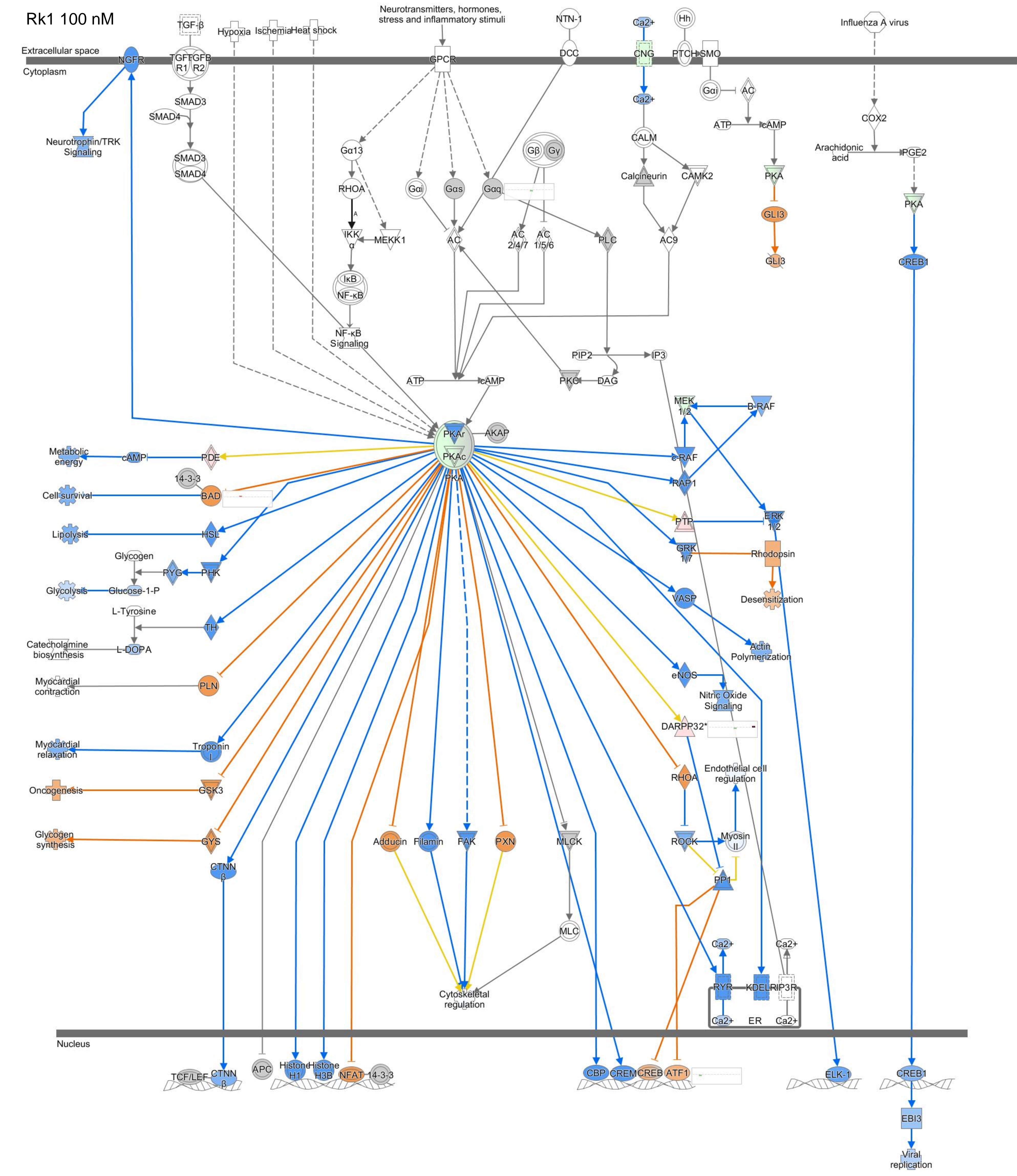

Supplement: Supplementary file 1 [file pharmaceuticals-14-01010-s001.zip › Supplement 5 Protein Kinase A signalling.pdf]

WG 10000 ng/ml

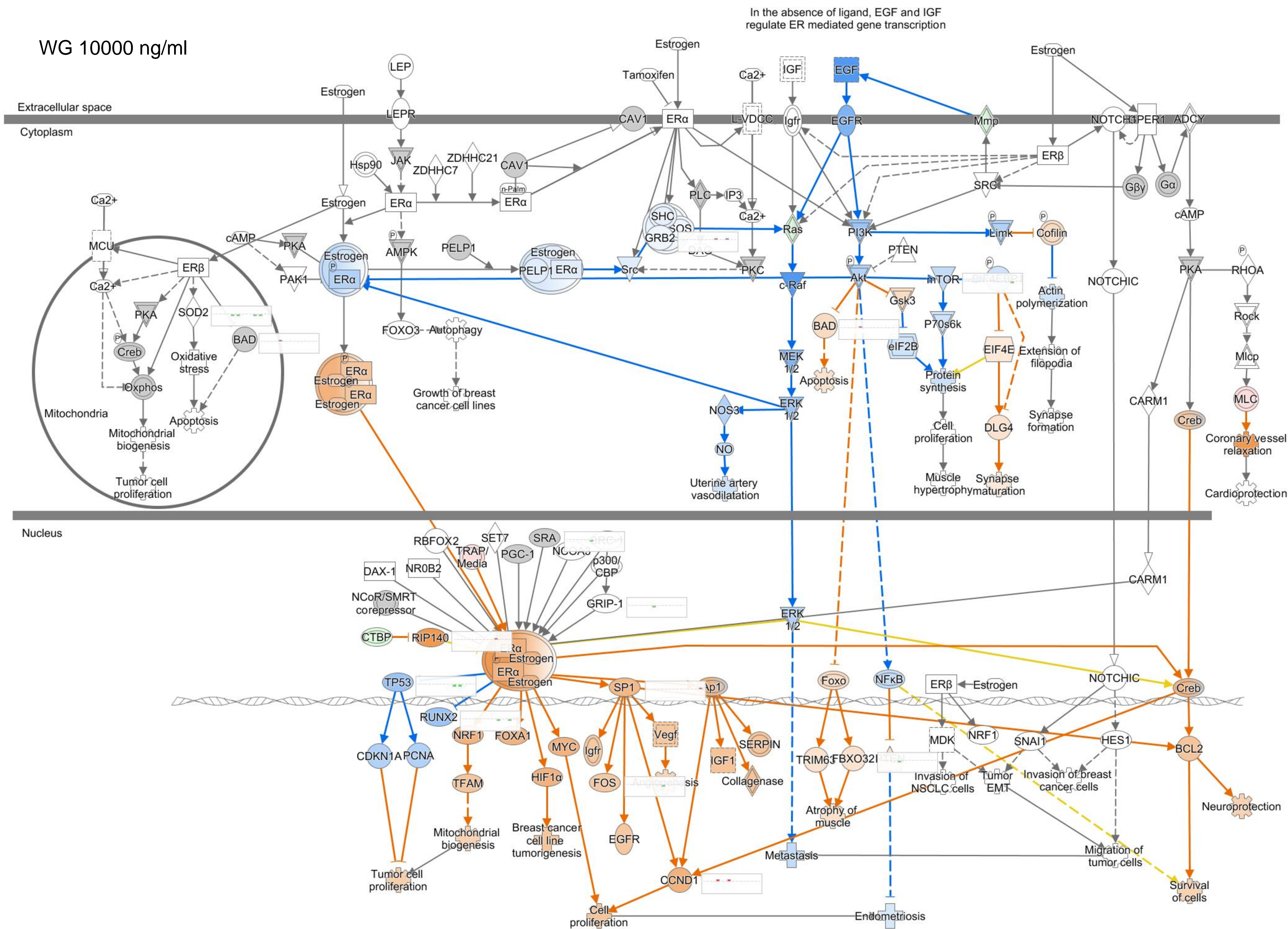

HRG80 10000 ng/ml

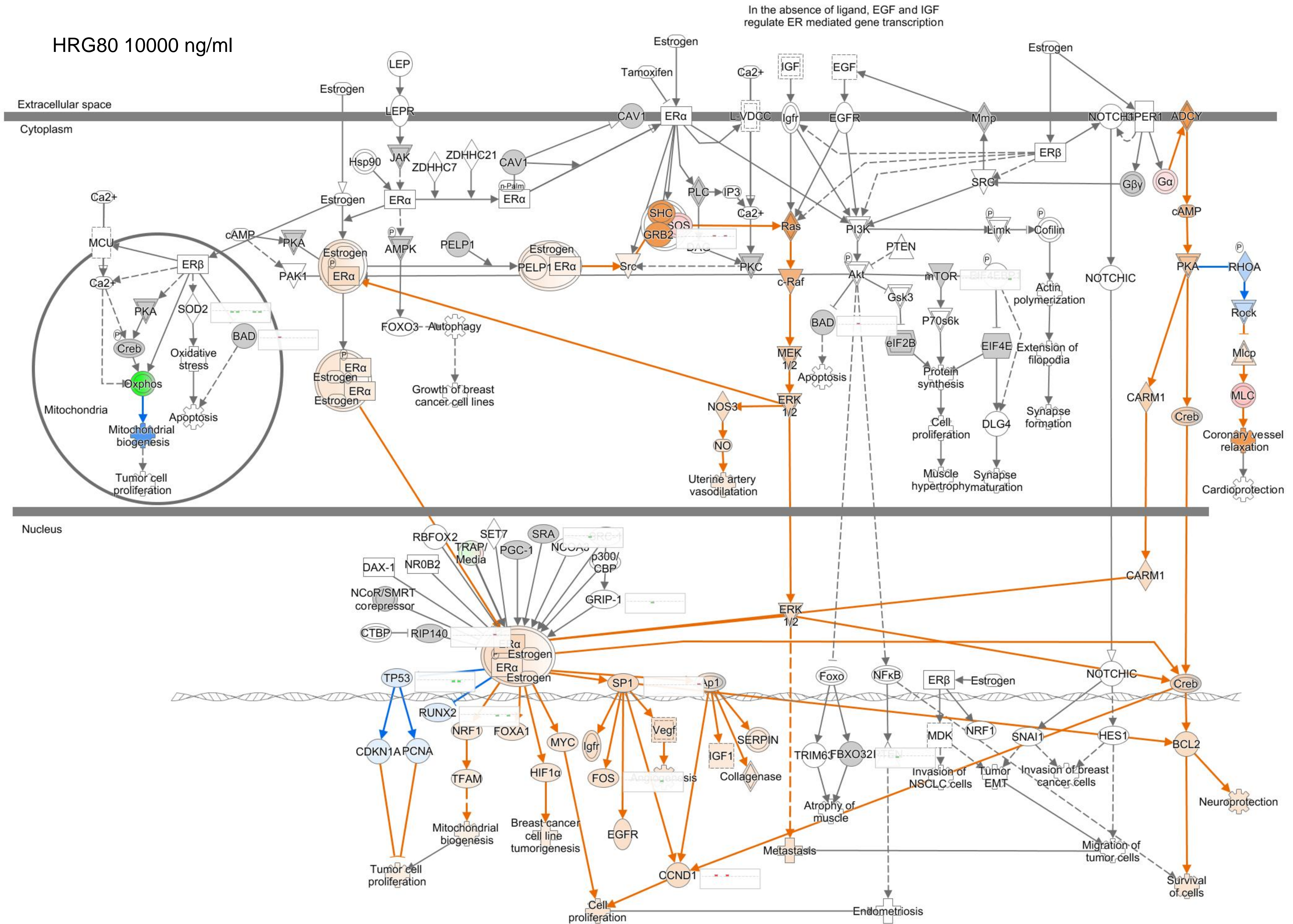

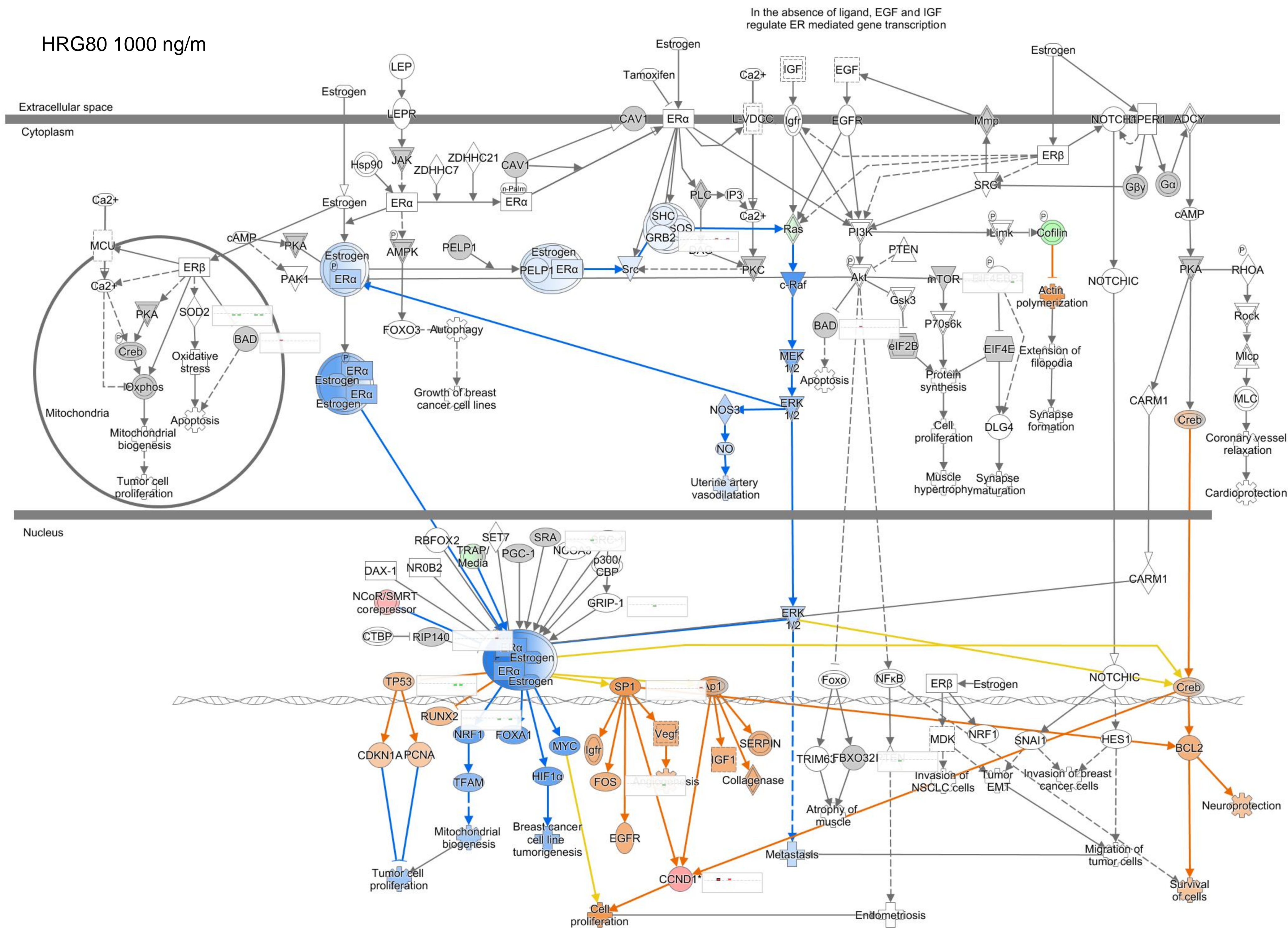

HRG80 100 ng/m

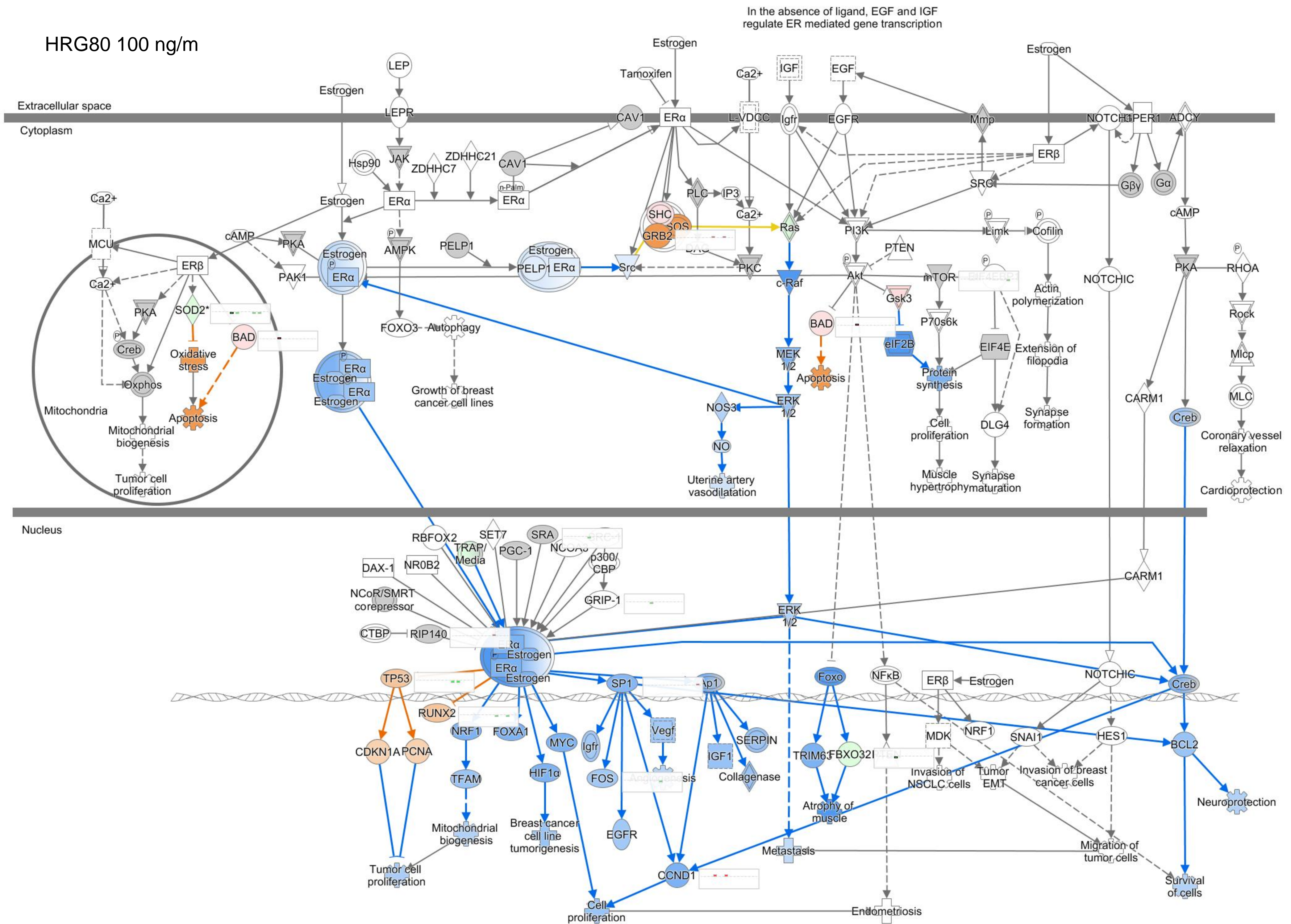

HRG80 10 ng/m

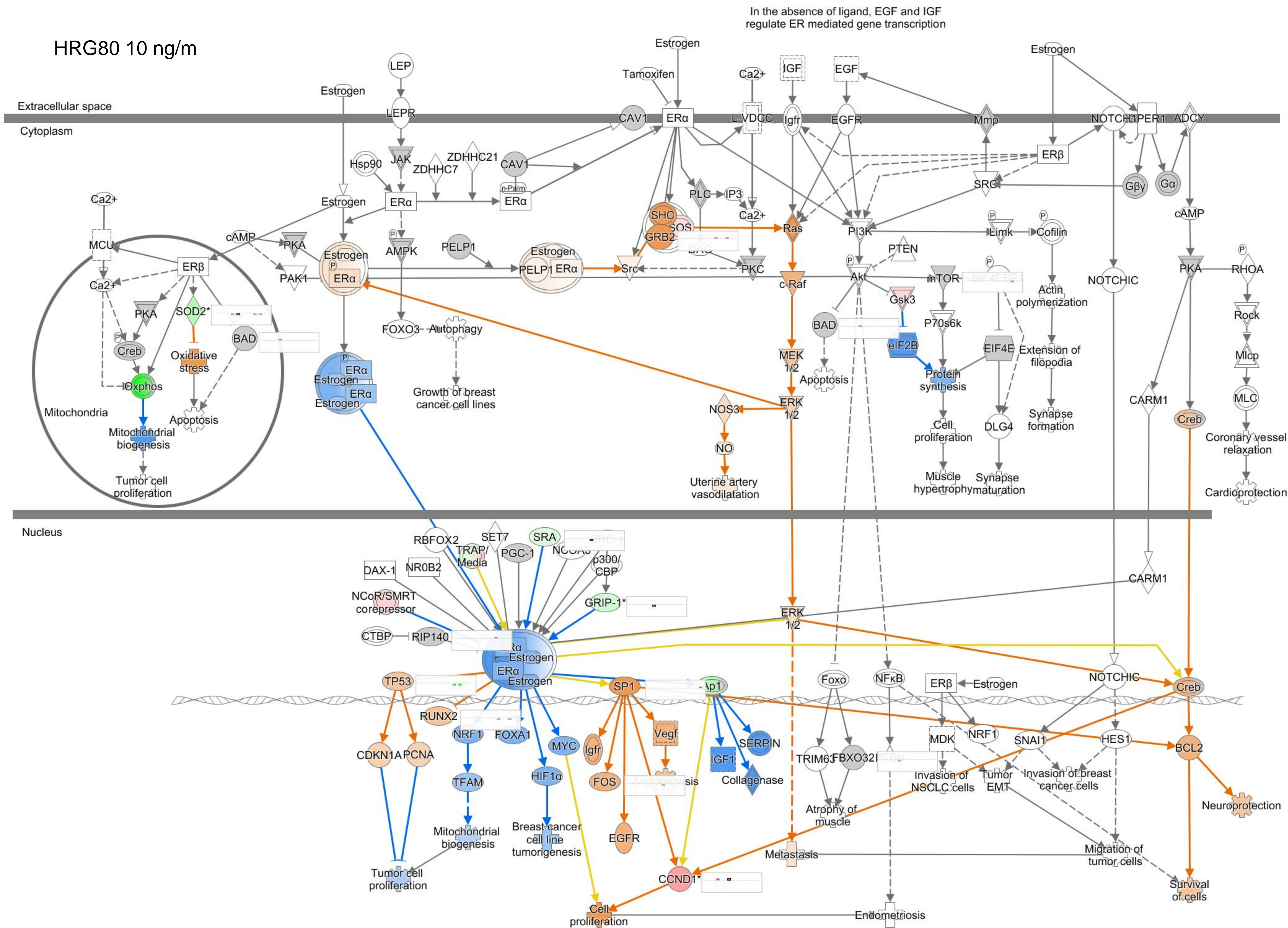

HRG80 0.01 ng/m

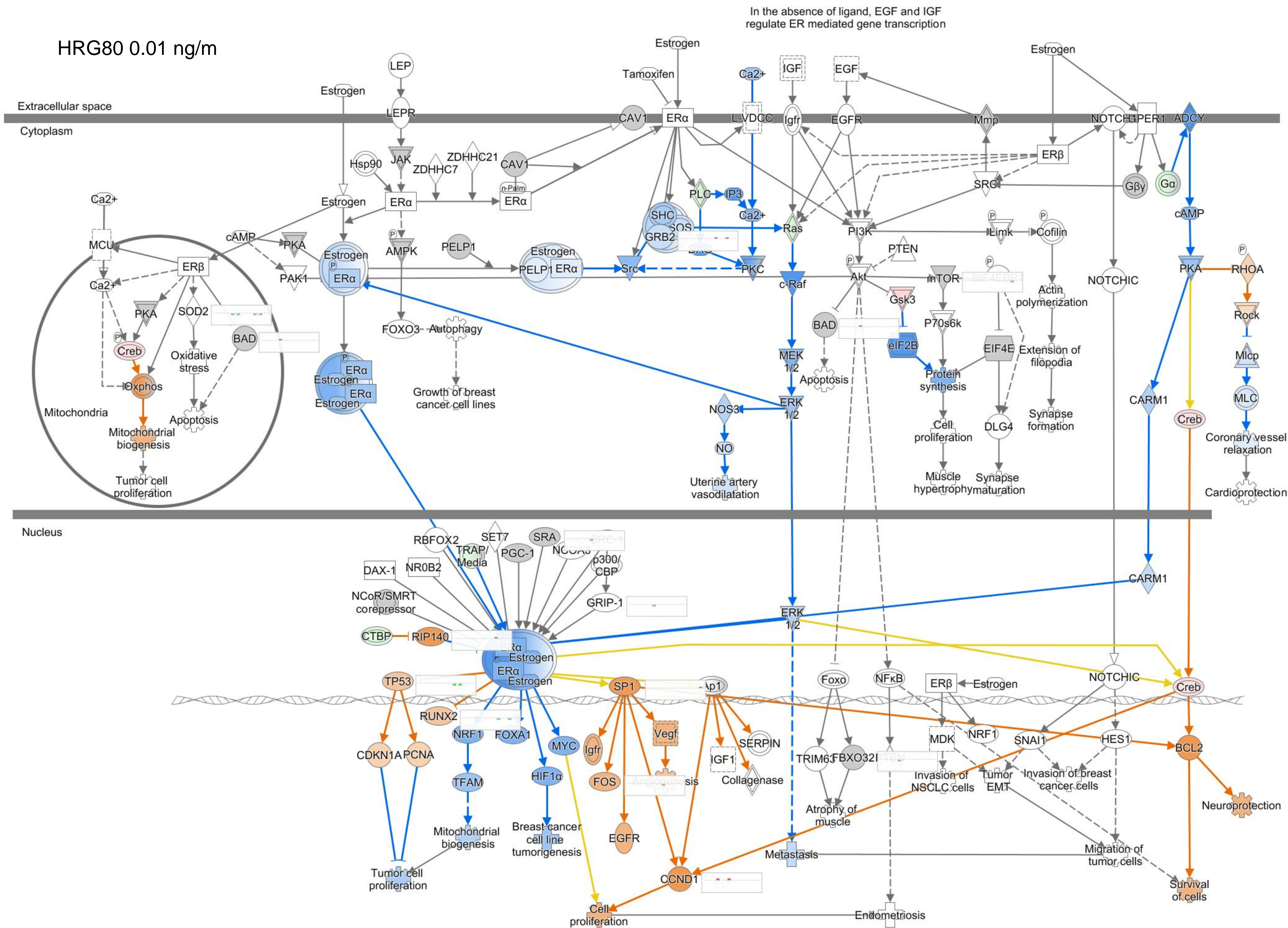

Rb1 100nM

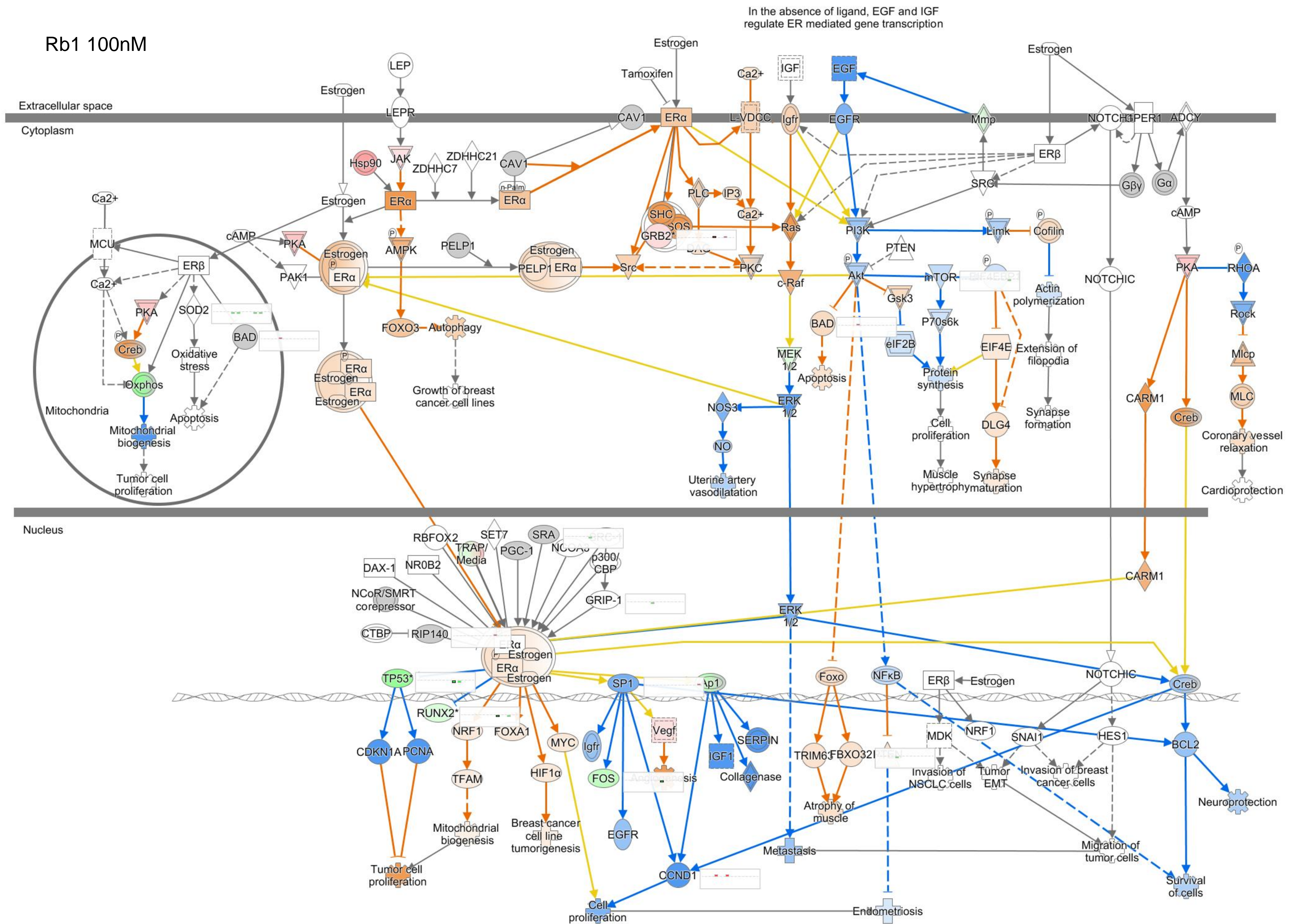



Rg5 100nM

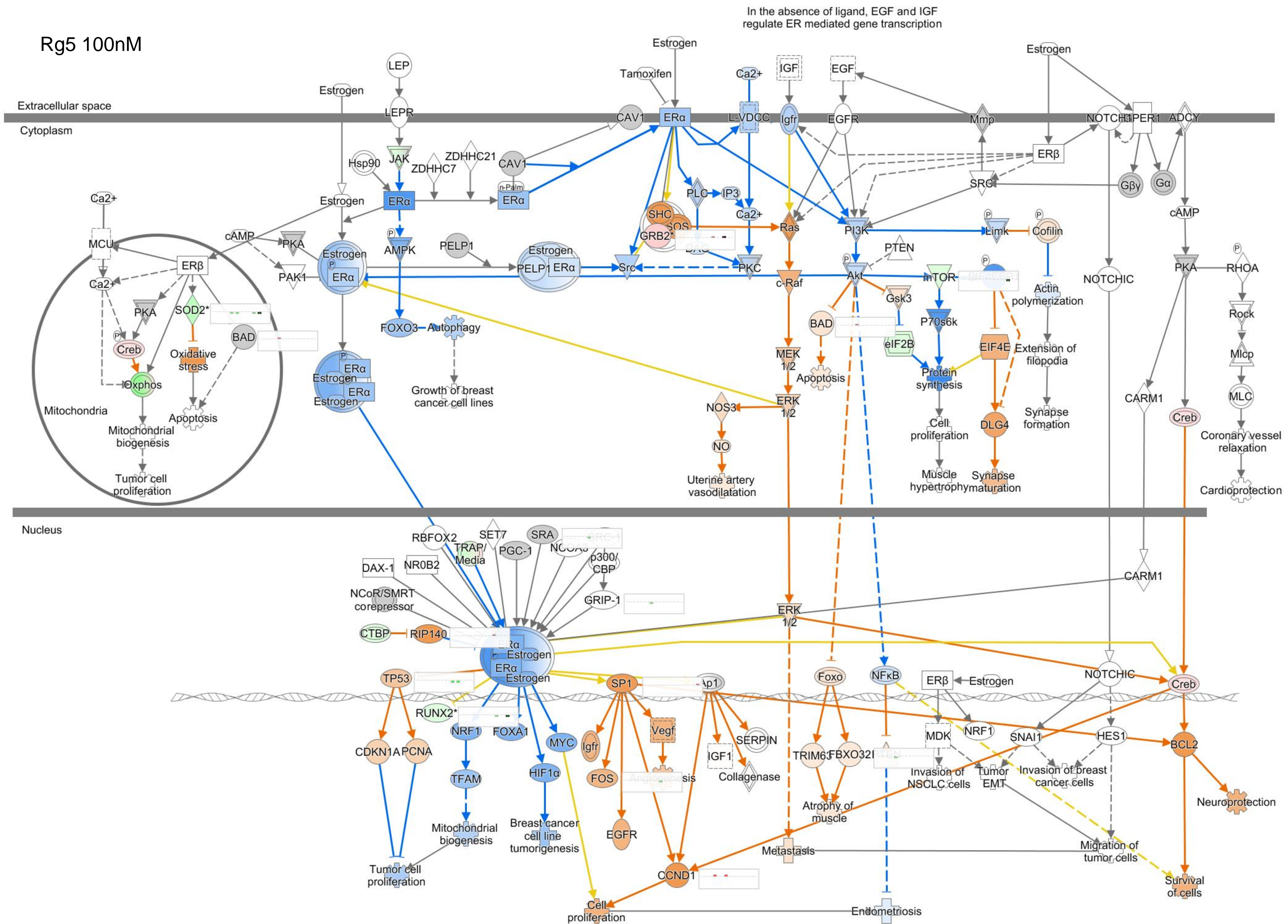

Rk1 100nM

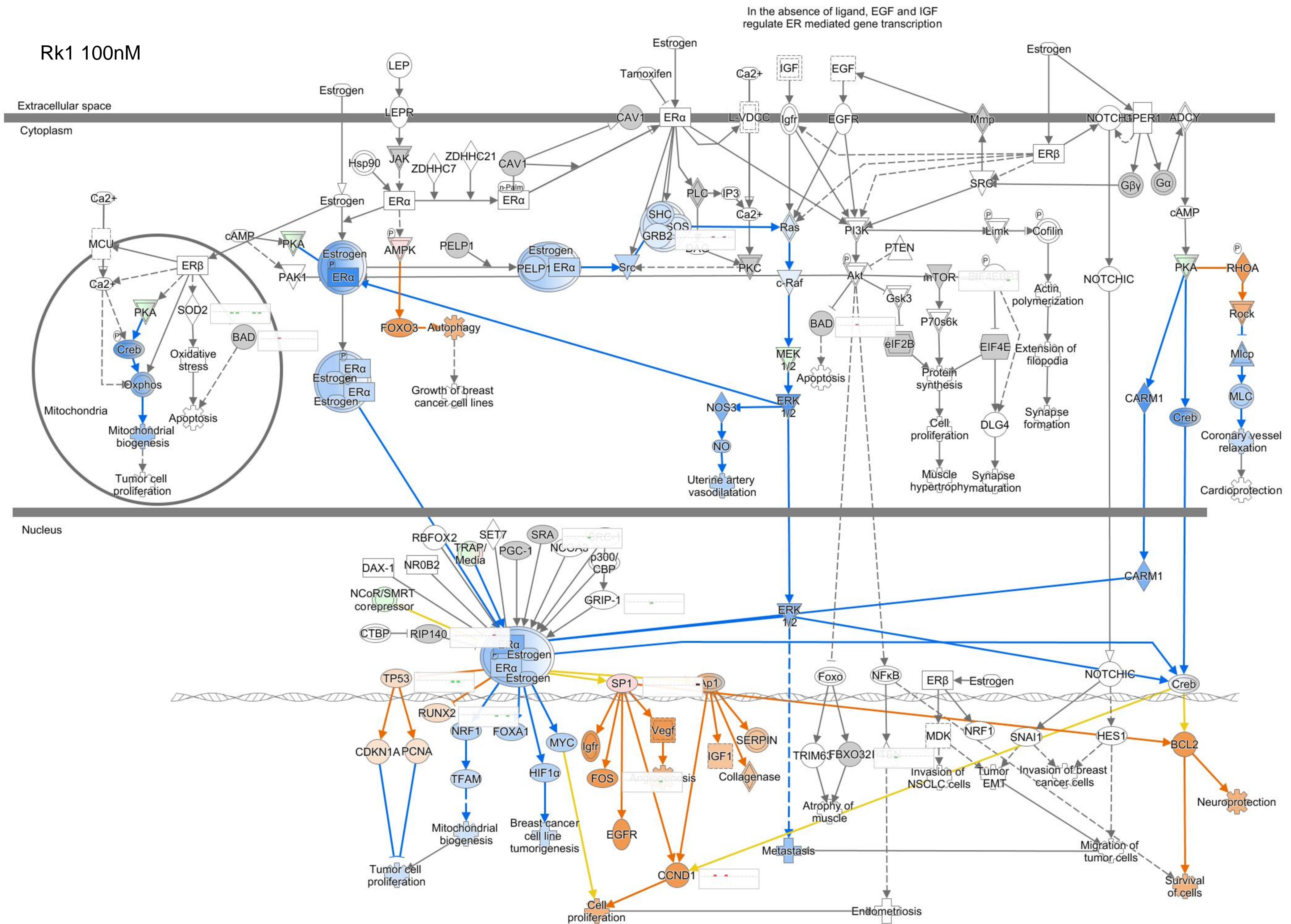

Supplement: Supplementary file 1 [file pharmaceuticals-14-01010-s001.zip › Supplement 7 Estrogen Receptors signaling.pdf]

WG 10000 ng/ml

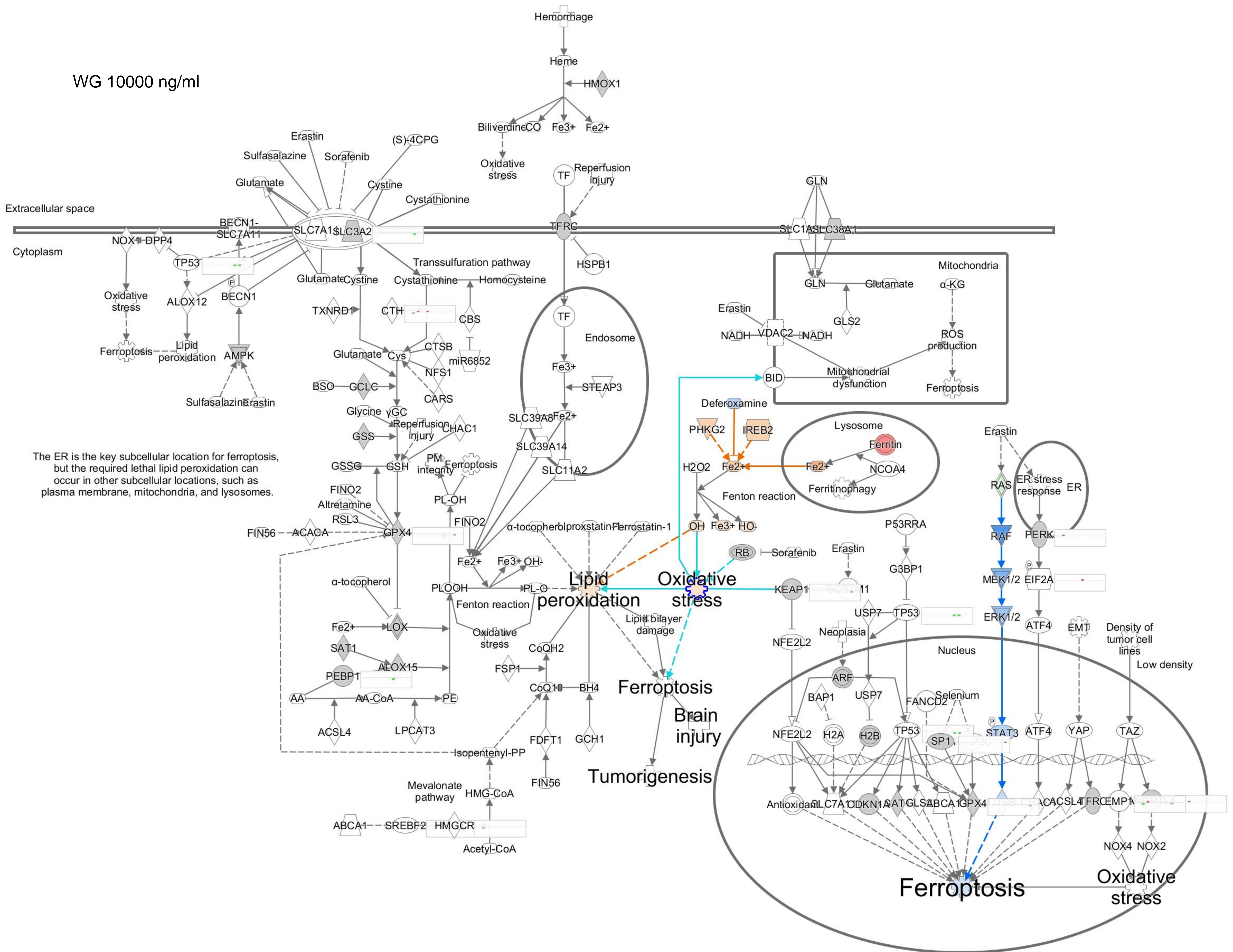

HRG80 10000 ng/ml

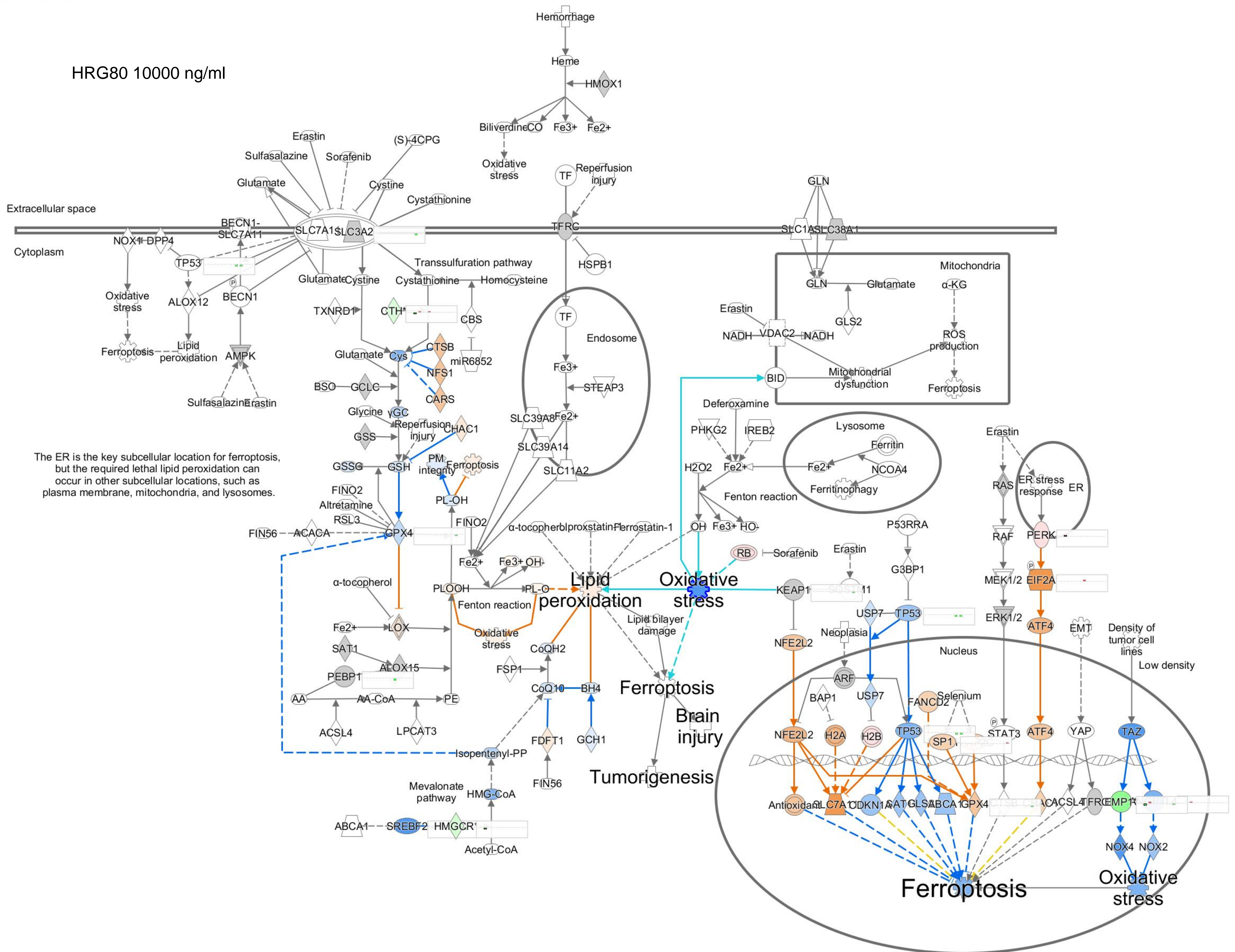

HRG80 1000 ng/ml

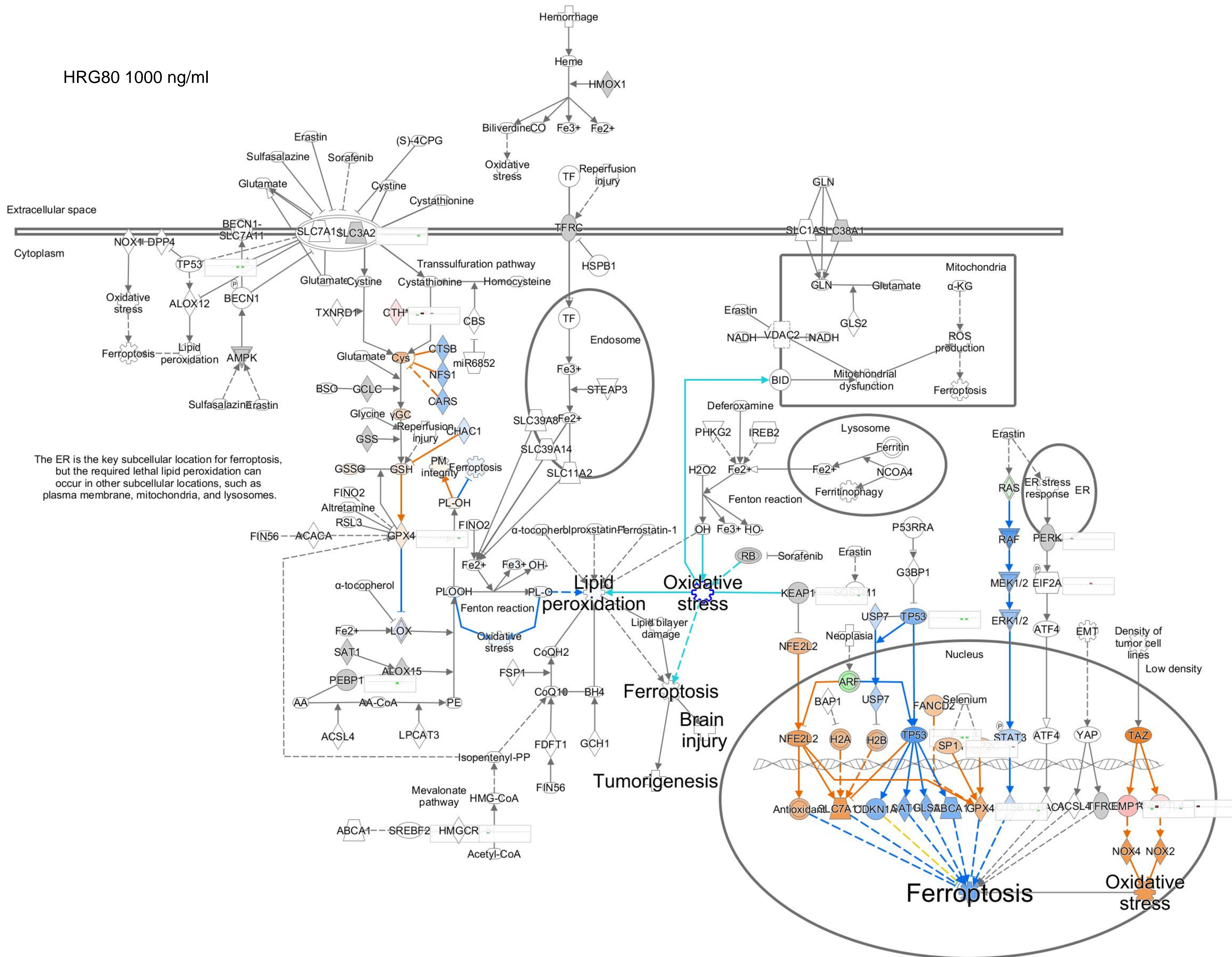

HRG80 100 ng/ml

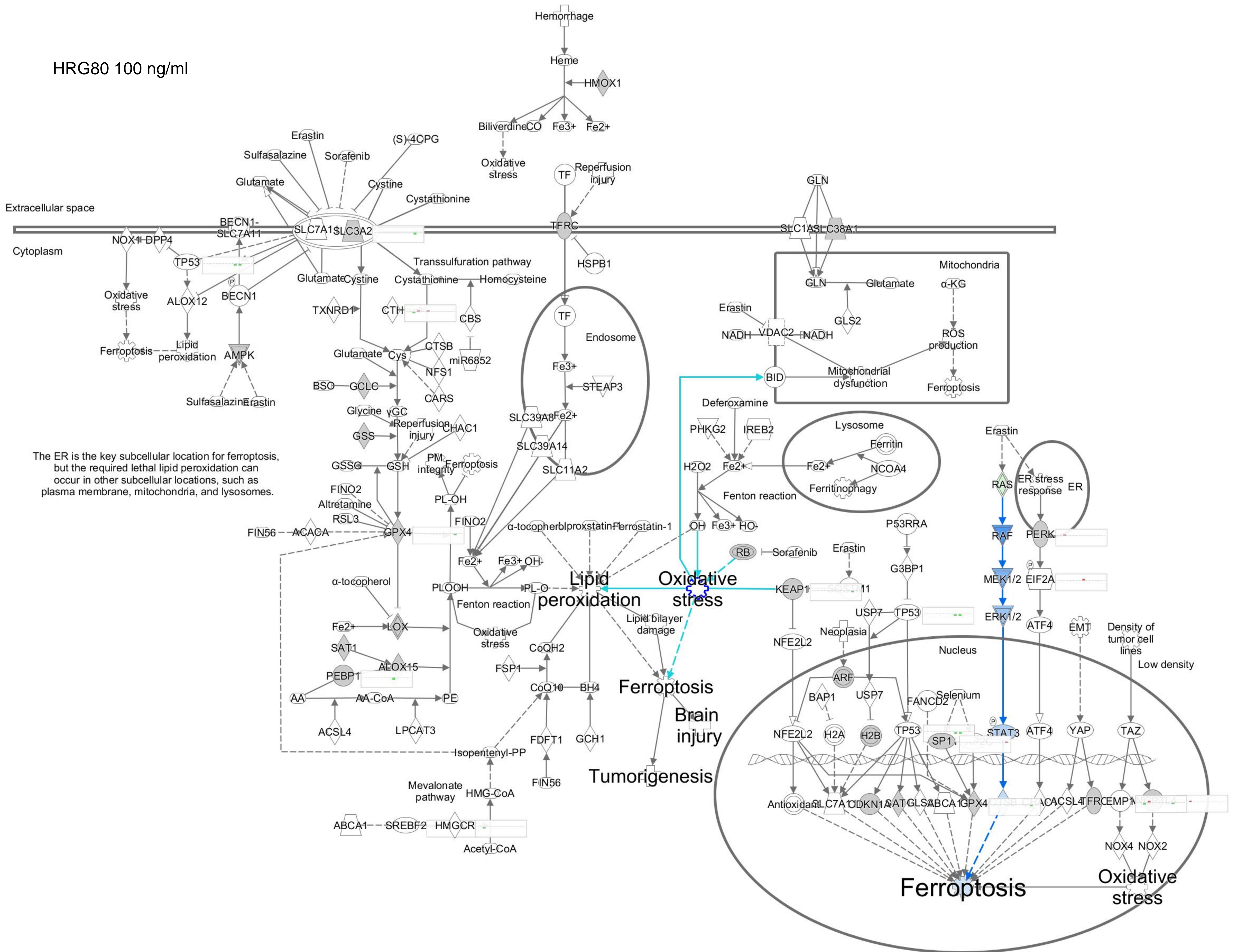

HRG80 10 ng/ml

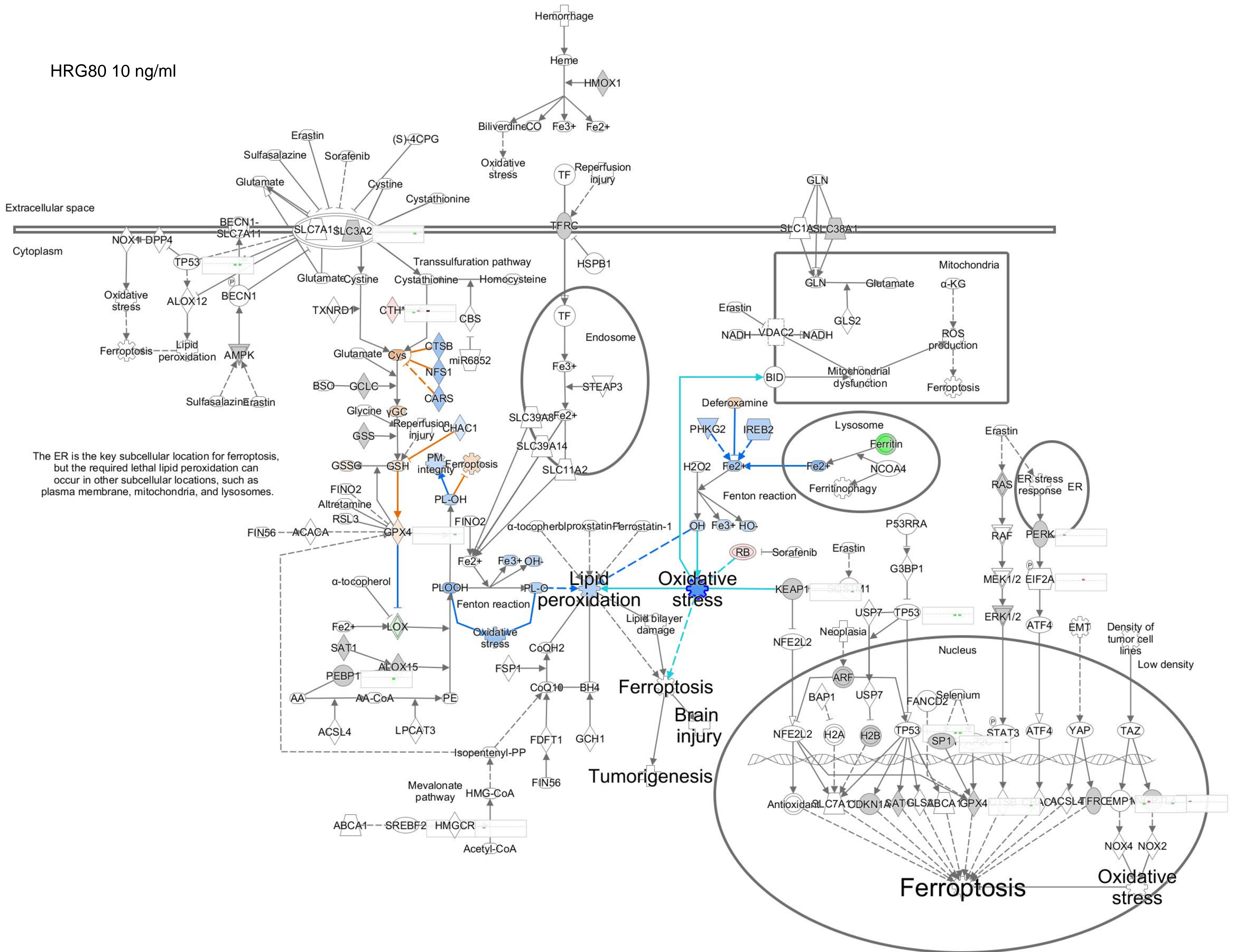

HRG80 0.01 ng/ml

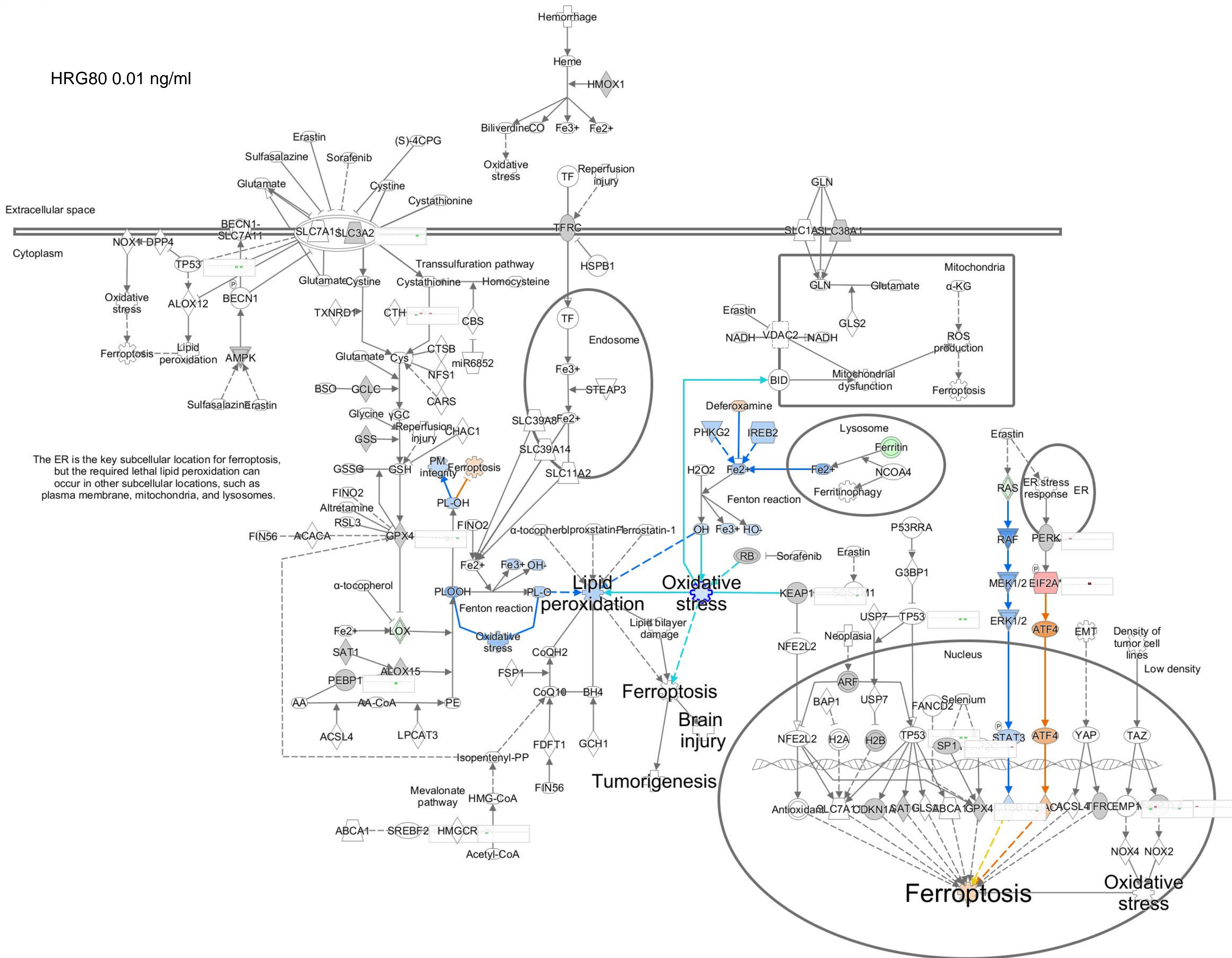

Rb1 100 nM

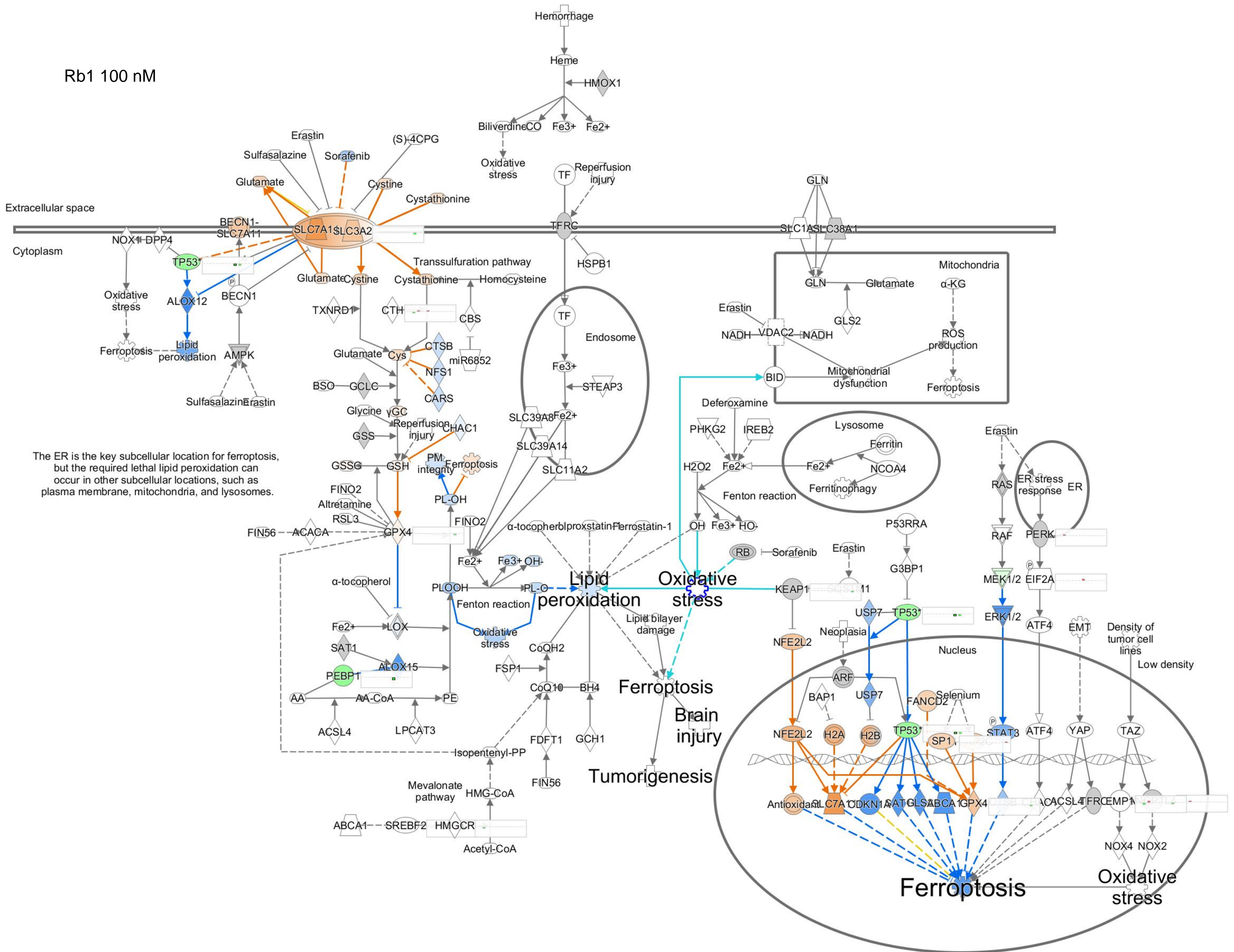

Rg3 100 nM

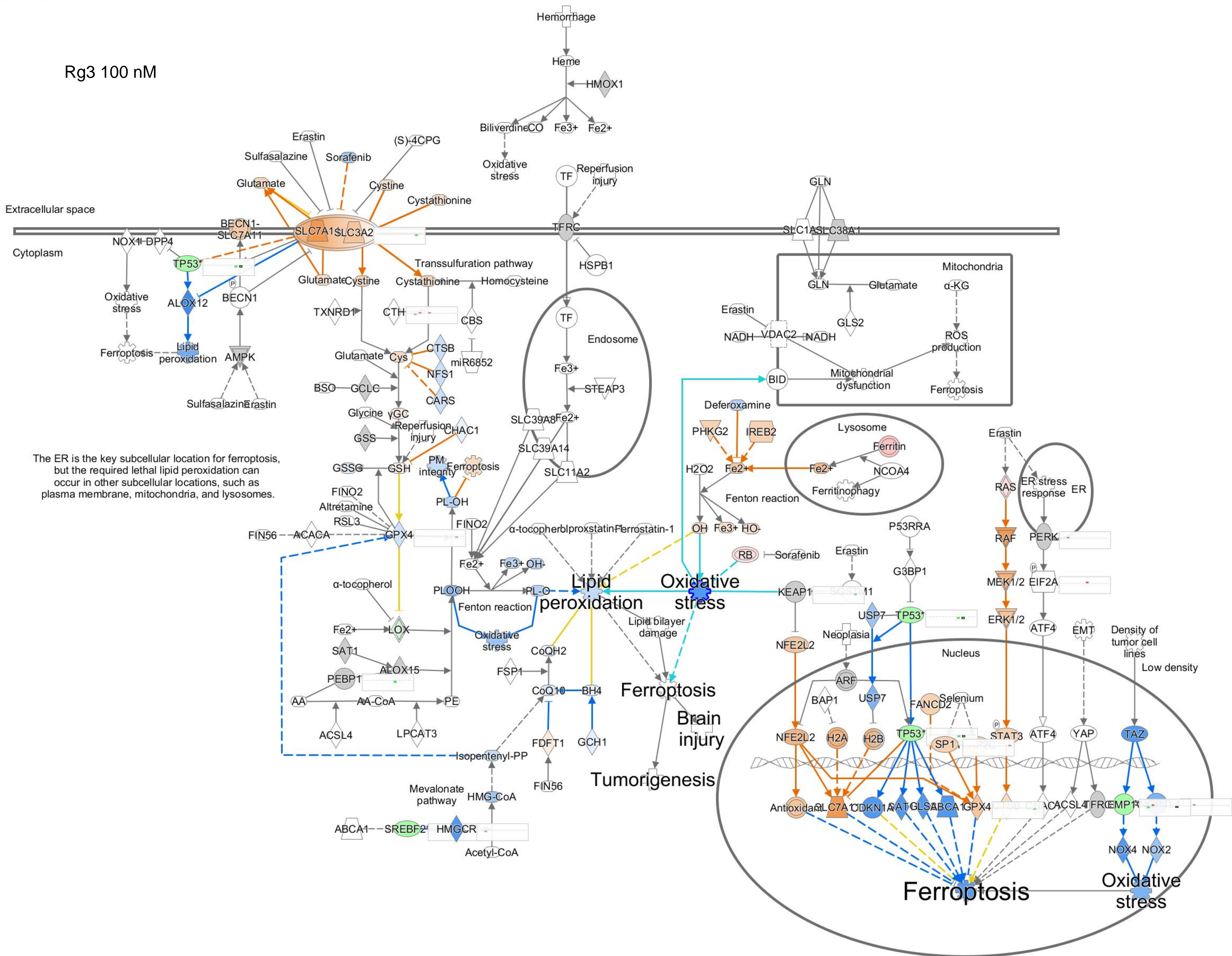

Rg5 100 nM

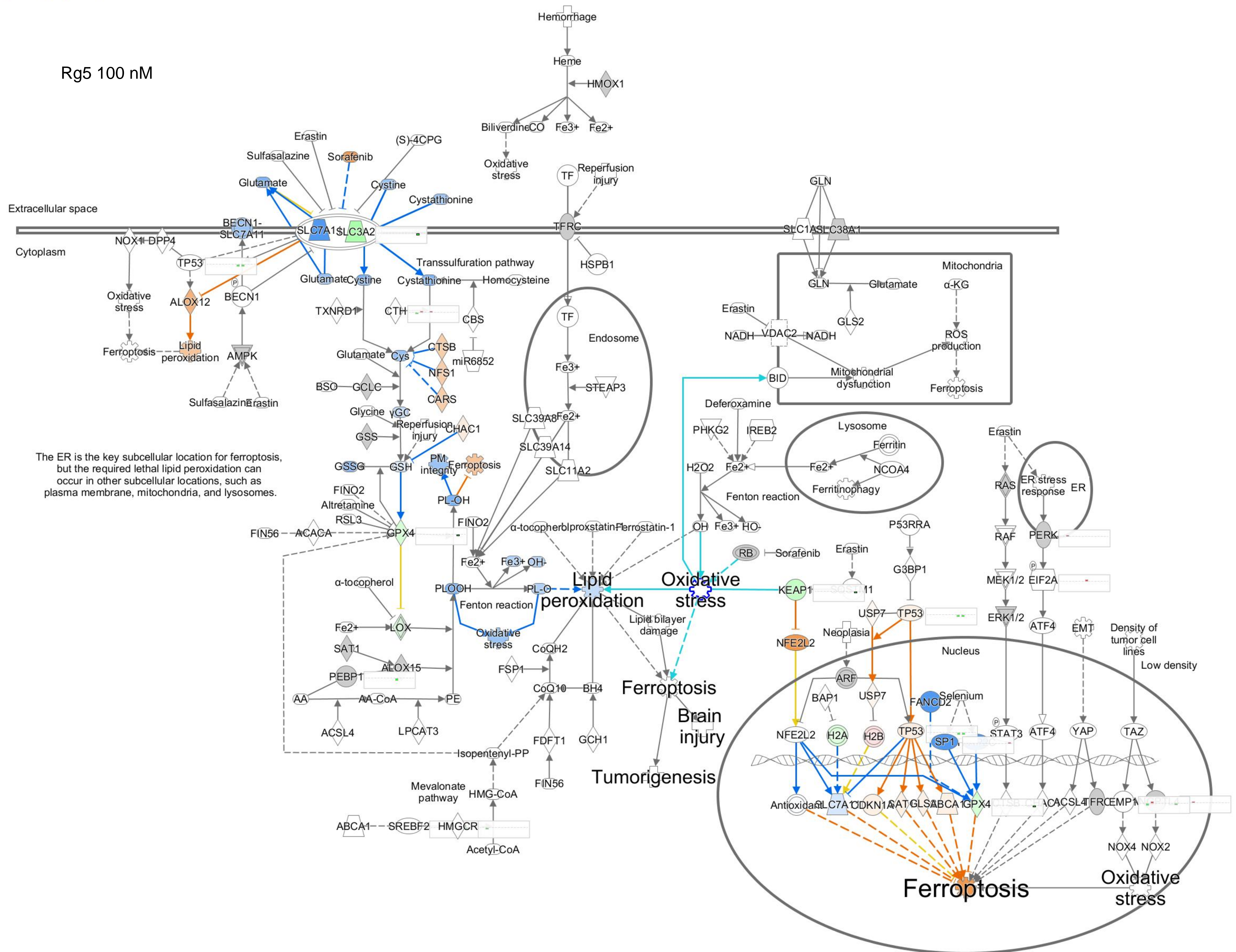

Rk1 100 nM

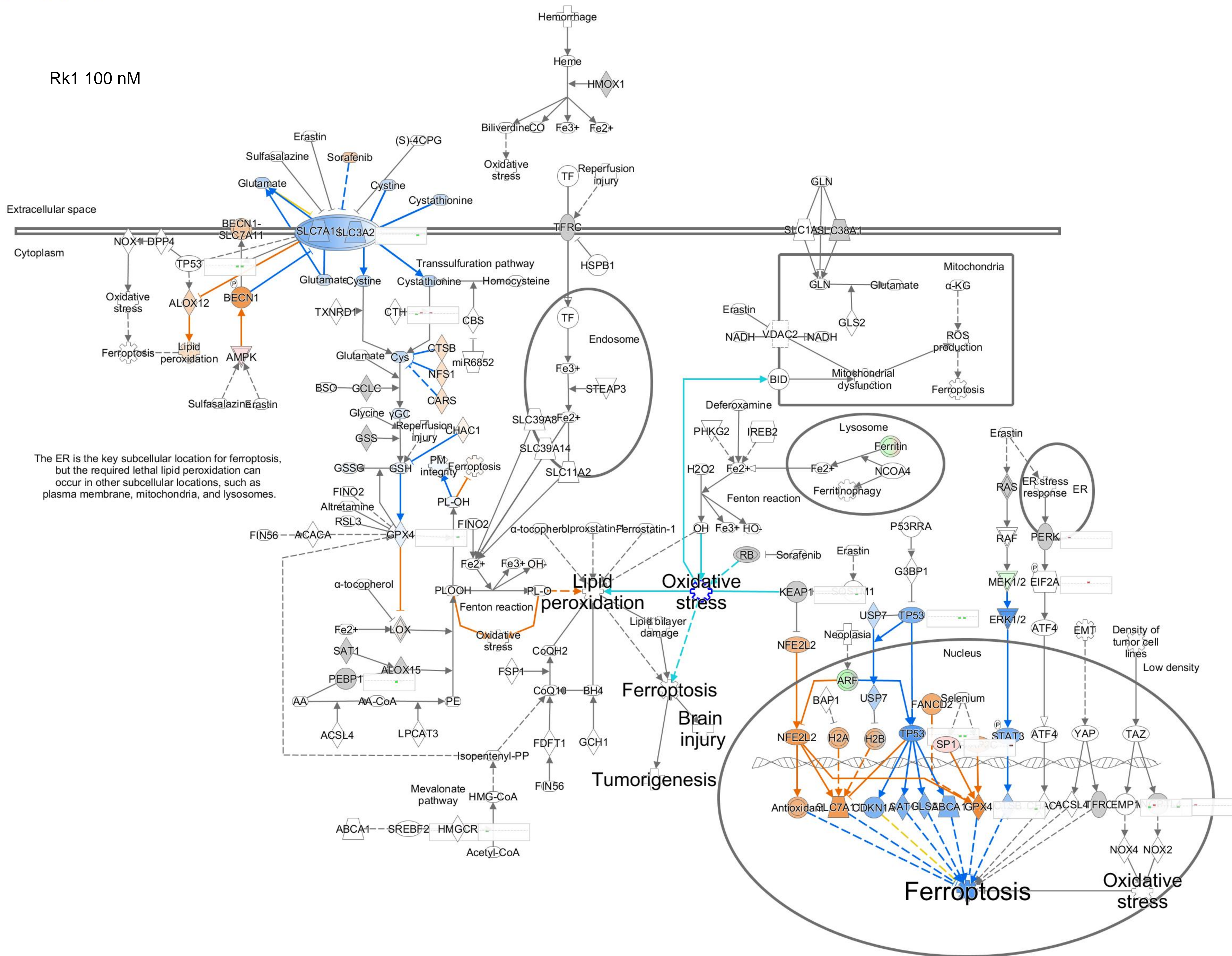

Supplement: Supplementary file 1 [file pharmaceuticals-14-01010-s001.zip › Supplement 8 Ferroptosis signaling.pdf]
